# Growth Mechanism of Micro/Nano Metal Dendrites and Cumulative Strategies for Countering Its Impacts in Metal Ion Batteries: A Review †

**DOI:** 10.3390/nano11102476

**Published:** 2021-09-22

**Authors:** Brindha Ramasubramanian, M. V. Reddy, Karim Zaghib, Michel Armand, Seeram Ramakrishna

**Affiliations:** 1 Centre for Nanoscience and Technology, Pondicherry University, Puducherry 605014, India; brindharamasubramanian@gmail.com; 2 Centre of Excellence in Transportation Electrification and Energy Storage (CETEES), Institute of Research Hydro-Québec, 1806, Lionel-Boulet Blvd., Varennes, QC J3X 1S1, Canada; 3 Department of Mining and Materials Engineering, McGill University, Wong Building, 3610 University Street, Montreal, QC H3A OC5, Canada; karim.zaghib@mcgill.ca; 4 Centre for Cooperative Research on Alternative Energies, Basque Research and Technology Alliance, Alava Technology Park, Albert Einstein 48, 01510 Vitoria-Gasteiz, Spain; marmand@cicenergigune.com; 5 Center for Nanofibers and Nanotechnology, Department of Mechanical Engineering, National University of Singapore, Singapore 117576, Singapore

**Keywords:** energy storage, dendrites, volume expansion, ion flux, 3D scaffolds, metal-ion batteries (Li/Na/K)

## Abstract

Metal-ion batteries are capable of delivering high energy density with a longer lifespan. However, they are subject to several issues limiting their utilization. One critical impediment is the budding and extension of solid protuberances on the anodic surface, which hinders the cell functionalities. These protuberances expand continuously during the cyclic processes, extending through the separator sheath and leading to electrical shorting. The progression of a protrusion relies on a number of in situ and ex situ factors that can be evaluated theoretically through modeling or via laboratory experimentation. However, it is essential to identify the dynamics and mechanism of protrusion outgrowth. This review article explores recent advances in alleviating metal dendrites in battery systems, specifically alkali metals. In detail, we address the challenges associated with battery breakdown, including the underlying mechanism of dendrite generation and swelling. We discuss the feasible solutions to mitigate the dendrites, as well as their pros and cons, highlighting future research directions. It is of great importance to analyze dendrite suppression within a pragmatic framework with synergy in order to discover a unique solution to ensure the viability of present (Li) and future-generation batteries (Na and K) for commercial use.

## 1. Introduction

The escalating demand for energy systems and transitory fossil fuel sources has driven the call for high energy storage devices [1,2,3,4,5]. Recently, batteries and fuel cell technologies have revolutionized diverse fields, from power tools to electric vehicles [6,7]. In particular, modern batteries with an extended lifespan, exceptional design, and cost-effectiveness remain ubiquitous in consumer electronics and industrial products. Furthermore, progressing demands for automobile feedstocks and alternate combustibles have satisfied the desire for multiple innovations and optimizations in battery chemistry [8,9,10,11,12,13,14,15,16,17,18,19]. The call for an efficient battery system with high storage capacity has resulted in the adoption of high-throughput electrode materials, which is a crucial determinant for future innovations [20,21,22,23,24]. Moreover, the anode material plays a vital and equal role in the comprehensive performance of high-energy-density battery systems [25,26,27]. It is possible that alkali metal anodes represent an option for such high-energy systems [28,29,30,31,32,33,34,35]. These metal batteries have shown strong potential in the energy market and are anticipated to be next-generation batteries [36]. Their high energy density and low-cost anode material make them superior to lead–acid batteries [37]. Alkali metals such as Li, Na, and K hold extraordinary reactivity and readily lose their valence electron (*ns*1), forming multiple cations. Due to the low ionization energy, these soft metals have low melting points (Li: 181 °C, Na: 98 °C, K: 63 °C) and are incredibly reactive [20,33,38,39]. Except for hydrogen, all the alkali metals are in solid form at room temperature [40]. Salt ions of these metals are comparatively larger than the other ions in the same period, which leads to a reduction in the charge density and is responsible for the separation of alkali ions from their counter anion while dissolving in aqueous electrolytes [41,42]. The complete structures and properties of the alkali metals, and other feasible metals employed in batteries, are presented in Table 1. 

**Figure 1:**
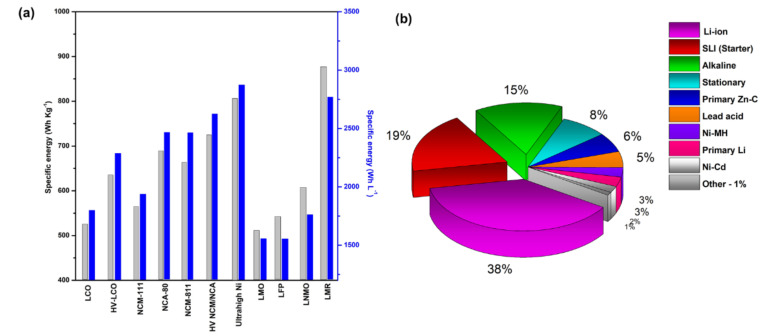
(**a**) Specific energy of commercial LIB electrode. (**b**) Current percentages of usage of different batteries.

**Figure 2:**
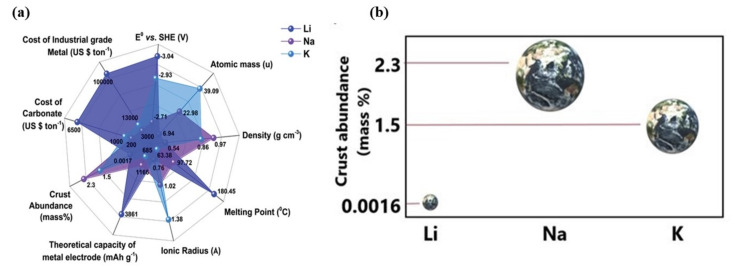
(**a**) Properties of alkali metals (adapted with permission from [51]. Adv Energy Mat., 2020). (**b**) Earth crust abundance comparison of Li, Na, and K.

Lithium-ion batteries (LIBs) are widely used in electric vehicles and consumer electronics, owing to their high energy density (265 Wh kg^−1^), longevity, high columbic efficiency, zero memory effect except for LiFePO_4_, low self-discharge rate (2% per month), and lower maintenance requirements [43,44,45,46,47,48,49]. Commercial Li batteries work with different electrode materials. Their specific energy and endurance over the years are shown in Figure 1a. At present, LIBs are becoming increasingly prevalent in the energy market, with a cumulative production rate higher than 100 GWh yr^−1^, surpassing lead–acid batteries. This production rate is expected to exceed thousands of GWh in the coming years [50]. Some other battery types and their percentages of usage are shown in Figure 1b. Among them, nickel, manganese, and iron-rich transition metal oxides, phosphates show higher commercial viability influenced by earth abundance and individual alkali metal properties (Figure 2a,b).

Consumer electronics predominantly utilize polymer electrolytes with a lithium cobalt oxide (LiCoO_2_) cathode and graphitic anode, yielding the optimum energy density [52]. Additionally, cathode materials such as lithium iron phosphate (LiFePO_4_), lithium–nickel manganese cobalt oxide (LiNixMnyCozO_2_) (x + y + z = 1), and lithium-rich or doped metal ion manganese oxide (LiMn_2_O_4_) structures offer better capacity and lifecycle [53,54,55,56,57,58,59,60,61,62]. These cathodes are extensively used in medical instruments and electric vehicles [63,64]. However, the periodic intercalation and de-intercalation mechanisms of commercial LIBs employing LiFePO_4_ and LiMn_2_O_4_ no longer support high-power applications with charge capacities greater than 250 mAh g^−1^ [65,66,67]. This shortcoming is mainly due to unstable SEI and dendritic structures, which affect the cyclic performance of batteries [68,69,70,71,72]. In recent years, researchers have focused on enhancing the electrode–electrolyte kinetics, lifetime, charging speed, specific energy, and safety of Li-ion batteries (LIBs) [18,22,73,74]. Several modifications and engineering methods for electrode design and electrolyte [75,76] interphase optimization provide the path towards future high-powered LIBs. However, Li reserves are limited; thereby, the cost of LIBs is extremely high [77,78]. The inadequacy of reserves and the expensiveness of LIBs have led to consideration of alternate metals such as Na and K-based batteries, which are believed to offer promise as future energy storage devices. Due to their abundance and low price, these alkali light metals (Na, K) hold promising positions as alternatives to Li-based batteries in the coming years [51,79,80]. The crust abundance and crucial properties for battery metals are shown in Figure 2a,b and Table 1.

Rechargeable batteries with Na and K metal anodes experience significant problems induced by dendrite outgrowth, reducing the active reaction mechanism of the battery [81,82,83,84,85]. Though the alkali metal anodes (Na and K) chemically react with almost all of the solid and organic electrolytes, the electrochemical reactions lead to elevated efficiency and enhanced operational extent only if a steady solid electrolyte interphase (SEI) layer is produced above the electrode surface to circumscribe unwanted surface reactions [86,87]. Furthermore, the electrode morphology and dendrite growth significantly influence the battery’s performance. During charge cycles, alkali metal ions accumulate erratically on the anode surface, developing intricate dendrite structures. These dendritic structures spread extensively, reaching the cathode and shorting the circuit, and they cause serious overheating in the battery components [88,89]. From further exploration, it has been observed that the alkali metal ions initially form clusters through nucleation. These cluster agglomerates create spiky structures that gradually evolve towards the cathode, comparable to stalagmite, an upward-growing mineral precipitated by water trickling in caves [90]. 

In general, the intercalation process in an alkali metal battery requires space for holding metal ions. However, in most cases, alkali metal anodes fail as a host and are unable to retain the multivalent ions, posing a risk to the electroneutrality. Host failure expedites the volume change, giving rise to the absolute depths of discharge profiles associated with unavoidable cracks at the SEI interface and electrode surface [91,92].

**Figure 3:**
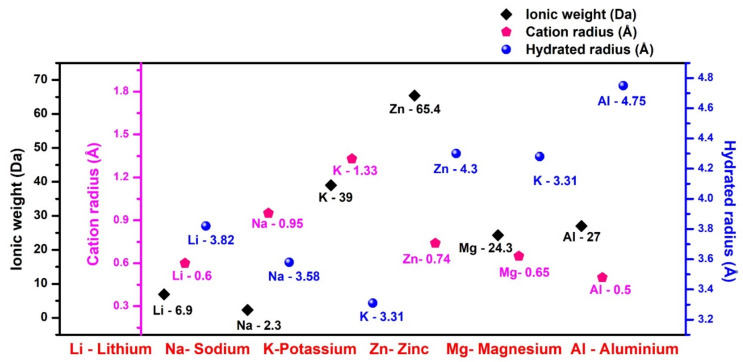
Comparison of ionic radius and weight among Li, Na, and K alkali metals.

As the SEI becomes unstable, extreme chemical reactions hamper the cyclic performance and result in the shortened lifetime of the anode, reducing its efficiency. In particular, Na- and K-based batteries carry more challenges than Li, related to inconsistent SEI and unstable passivation layers, as Na and K differ from Li in terms of electrochemical reactivity, mechanical characteristics, volume expansion, and decomposition product accumulation due to their larger ionic radius, as shown in Figure 3, and poor Lewis acidity [93]. The various challenges mentioned above, and the possible strategies to resolve them, are comprehensively discussed in this review. First, before discussing the main theme of the article, a few insights into batteries and their materials are provided.

## 2. Battery Insights—Material Perspectives

**Figure 4:**
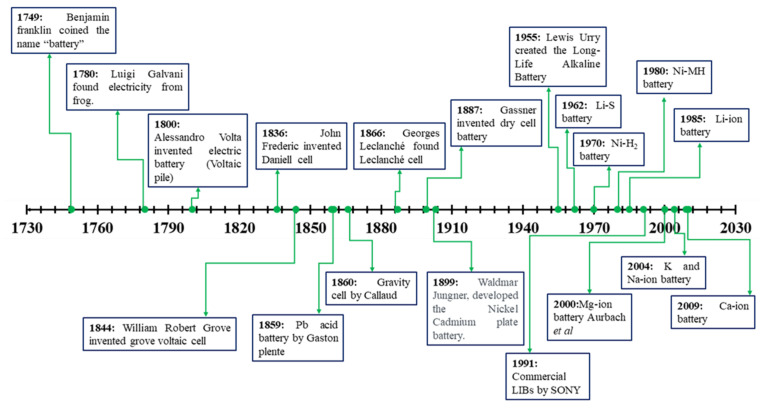
Timeline of battery development from 1730 to 2020.

The catchword “battery” was introduced by Benjamin Franklin in 1749 to describe coupled capacitors. Then, Luigi Galvani devised bioelectricity from dead specimens of a frog in 1782. Later, various scientists and chemists developed diverse batteries, as depicted in Figure 4. In the last decade, the Goodenough–Kanamori precepts for ascertaining the magnetic superexchange in Li-ion batteries and progress in machine dynamics (i.e., random-access memory) have led to more advanced technologies.

In the 1990s, the Li-ion battery provoked a revolution in consumer and portable electronics. In particular, the state-of-the-art LIBs with an average energy density of 250 Wh Kg^−1^ started a paradigm conversion towards gasoline alternatives [94]. However, its energy density is assuredly low for complete gasoline replacement. Consequently, widespread efforts are currently underway to produce high-energy batteries surpassing lithium-ion batteries [95]. Present-day commercial LIB technology mostly adopts insertion compounds and carbon-based electrolytes [96,97]. Graphite is conventionally used as an anode, with a working potential of ~0.1 V vs. Li/Li^+^, due to its low cost and high cycle life, but it has limitations in terms of its specific capacity (372 mAh g^−1^), along with the intercalation of one metal atom for six carbon atoms (LiC_6_) [98,99]. Instead, Li metal foil as an anode can potentially increase the specific capacity to 1150 Wh Kg^−1^, competing with gasoline [100,101,102,103].

The conventional lithium-ion battery operates via lithiation and de-lithiation mechanisms. The metal anodic terminal, which works as the battery’s negative electrode, is lithiated to a potential of ~0.09 V vs. Li/Li^+^ [104] in the presence of organic electrolytes such as ethylene carbonate, dimethyl carbonate, propylene carbonate, ethyl methyl carbonate, diethyl carbonate, etc. The anodic oxidation–reduction energy levels lie below the lowest unoccupied molecular orbital (LUMO) of the above-mentioned electrolytes, giving rise to electrode decomposition [105,106,107]. However, during the charging process, a stable passivation layer or SEI blocks the electrode–electrolyte decomposition. Furthermore, current collector additives are employed to inhibit anodic and cathodic corrosion. Cathode materials are optimized to lessen the loss of oxygen in order to prevent thermal runaway at elevated temperatures. Moreover, the metallic Li insertion–reinsertion process has drastic effects on volume expansion, impacting the battery kinetics. In order to address the problems associated with the volume change, decomposition of electrodes, and side reactions, a stable SEI layer must form on the electrode surface and remain intact after several battery cycles. Commonly used Li anodes are listed in Table 2 [108,109,110,111,112,113,114,115,116,117,118,119,120,121,122,123,124,125,126,127,128].

### 2.1. Materials for Li-Based Batteries

Among the Li-based intercalation electrodes, layered lithium cobalt oxide (LiCoO_2_) is the most successful cathode in Li-ion batteries commercialized by SONY electronics. Its early history regarding intercalated compounds has been discussed by Reddy et al. [95]. During the charge–discharge cycles, the Co and Li occupy octahedral sites, alternatively forming hexagonal symmetry. However, the Co and Li undergo several phase transitions during the lithiation and delithiation process [129]. Under extreme charging exceeding 4.5 V, LiCoO_2_ transforms from a hexagonal to a hybrid rhombohedral–hexagonal structure. Subsequently, these disorder transitions prohibit Li^+^’s diffusivity, generating significant mechanical stress and micro-cracks, leading to cyclic fading. In addition, increasing the cost of Co and reduced thermal steadiness due to the exothermic reaction of O_2_ and Li at high temperatures limit the use of LiCoO_2_ [129]. To extend the capacity of LiCoO_2_, various dopants (Mg, Al, Fe, Ni, Mn, Cr, and Fe) and nanophase surface coatings such as AlPO_4_, carbon, LiNbO_3_, ZrO_2_, TiO_2_, etc., by atomic layer deposition and solution coating have been performed [130,131,132,133]. These techniques have increased the operating voltage of LiCoO_2_-based batteries up to 4.35 V, with a maximum capacity of 165 mAh g^−1^ [134]. 

**Figure 5:**
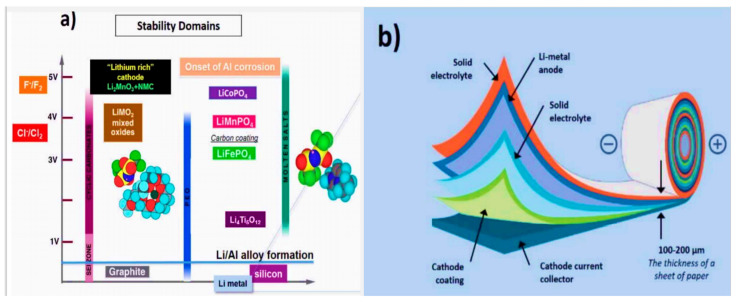
(**a**) Steadiness of diverse electrolytes and (**b**) solid-state lithium metal polymer battery architecture proposed by Michel Armand (adapted with permission from [95]. Materials, 2020).

Lithium nitrate (LiNO_2_) holds a non-experimental capacity of 270 mAh g^−1^. Further cathode materials include spinel structures (LiMn_2_O_4_ or LMO) and olivine structures (LiFePO_4_ (LFP)). The abundance and low cost of Mn and LMO-based batteries represent some advantages over other cathode materials. Nevertheless, LMO has structural instability (changes from spinel to layer), and Mn has a higher dissolution and leaching tendency, which leads to destabilization of the passivation layers [12,14,15,16,74,135]. Various surface coatings, such as AlPO_4_, ZrO_2_, LiNbO_3_, ZnO, and Mn-rich metal [135,136,137,138,139], stoichiometric modifications, and additives have been employed to rectify these issues. Later, polyanions were actively used, such as LiFePO_4_ and LiMnPO_4_. These materials possess enhanced thermal stability and cyclic capacity as Li^+^ ions occupy octahedral sites with phosphate in tetrahedral lattice sites, forming a hexagonal, closely packed oxygen array with high structural strength. However, these cathodes suffer from low electronic and ionic conductive formulations. Several other cathodes, such as TiS_2_, TiS_3_, WS_2_, MoS_2_, and NbSe_3_, were analyzed as intercalation hosts [58,140,141,142,143,144]. TiS_3_ showed multiphase transitions from trigonal to octahedral upon lithiation, similar to LiCoO_2_. Conversion cathodes such as FeF_2_ and CuF_2_, which structurally change with the breakage and formation of chemical bonds, have shown significant improvements. Fluorine- and chlorine-based metals (FeF_2_, NiCl_2_) have been utilized as cathodes. Though these metals have drawbacks, such as hysteresis losses, poor conductivity, and enormous side reactions, their open structure favors ionic conductance. The stability of common electrolytes in LIBs and the solid-state polymer battery architecture is shown in Figure 5a,b.

Recently, sulfur cathodes have been employed in Li batteries owing to their high theoretical capacity of 1675 mAh g^−1^. The high storage capacity of sulfur is due to the electrochemical cleft and reorganization of sulfur bonds in the cathode. One of the significant benefits of Li–sulfur batteries is their reduced weight compared to Li-ion batteries due to their moderate electrode loads. In general, sulfur is ample, environmentally benign, and readily available primarily from the Earth’s crust. Moreover, Li–S batteries are mostly resilient and are not easily damaged in harsh environments. Despite being superior, Li–sulfur batteries are associated with some complications and constraints. The major drawback with sulfur-based cathodes is their shuttle mechanism [145,146]. While aiming for high efficiency, the sulfur electrode must remain in close contact with a conductive layer (e.g., carbon) since sulfur is non-conductive. Moreover, there is concern regarding the safety of the Li electrode associated with passivation layers. Li–sulfur batteries’ periodic charge and discharge cycles can cause moss-grown spike-like deposits (dendrites) on the surface of the Li anode, which can severely degrade the battery’s overall performance. Additionally, spike layers can block the movement of Li ions responsible for the current flow. Sluggish conversion reactions of the sulfur electrode during charge–discharge cycles result in lower sulfur utilization and create a severe shuttling effect to disrupt the electrochemical kinetics [147]. Further, the dissolution of the electrode material in the electrolyte leads to a significant change in the structure and shape of the electrodes, reducing their cyclic stability. The self-discharge property and instability of Li-anode insoluble lithium polysulfide are other major drawbacks as the self-discharge products transmit back to the sulfur cathode, causing a re-oxidation reaction. Eventually, the battery capacity, lifetime, and columbic efficiency decline, affecting the overall efficiency of the battery [4,148,149]. 

**Figure 6:**
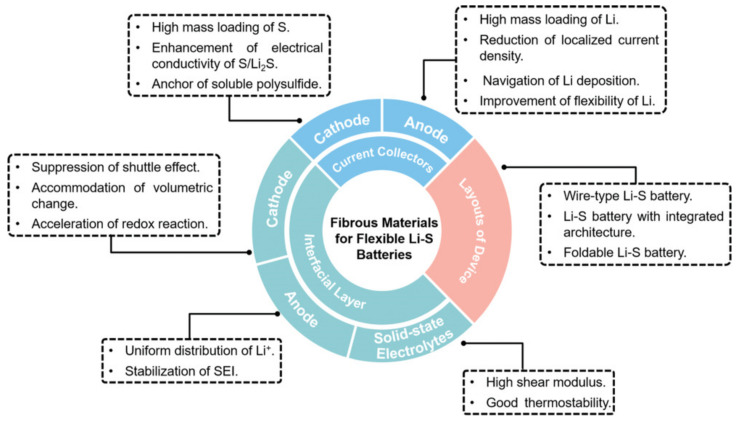
Advantages of fibrous framework in metal–sulfur batteries (adapted with permission from [157]. Adv. Ener. Mat., Wiley, 2020).

Many researchers have addressed the issues of Li–S batteries as they are expected to become a powerful, next-generation battery with high theoretical capacity and fewer toxins. Some of the recent advances in Li–S are briefly discussed below in order to provide a detailed understanding of the developments in metal–sulfur batteries. Xu et al. introduced the presence of halogen in transition metal cathodes using silver iodide as a host material in Li–S batteries [150]. The synergic effect of Li-iodide and silver during the electrochemical process enhances the ionic conductivity, inhibiting the polysulfide shuttle. Similarly, phosphide-based transition metals as host separators in Li–S batteries improve the operating performance due to the excellent catalytic activity and high conductivity of phosphides [151]. Carbon materials have strong potential to reduce the polysulfide shuttle effect as they adsorb firmly with Li-polysulfides and possess high conductivity [152,153]. A core–shell encapsulated sulfur cathode with nanoparticles reduces the polysulfide shuttle. Sulfur shells with MoS_2_, FeS_3_, MnO_2_, TiO_2_, SiO_2_, Ga, Ag, PANI, and PDA and polymer core nanoparticles are some of the advanced cathodes used in Li–S batteries, enabling more reliable electrochemical activity [154]. In addition to these efforts, the control of dendrites in the Li–S battery is a current research hotspot. Recently, a nitrogen-doped Ti_3_C_2_ Mxenes host, fabricated by 3D printing, has been proposed as a dendrite suppresser. As the porous nitrogen-rich Mxene structure provides more active sites for the lithiophilic–sulfiphilic process, dissipating the current, the deposition of Li becomes uniform over the anode, leading to a longer lifespan of 800 h at 5 mA cm^−2^ [155]. A graphitized 3D framework with high porosity offers tunnel confinement, allowing undisturbed ion transport and a dendrite-free anode in Li–sulfur batteries [156]. Fibrous materials have increased the surface to volume area, which provides high mass loading [157]. Insights into nanofibrous, porous frameworks and 2D and 3D structures may lead to dendrite and polysulfide shuttle control in sulfur batteries. Figure 6 presents the advantages of using fibrous materials as cell components in metal–sulfur batteries. Solid-state metal–sulfur batteries are another viable option to regulate dendrite growth. It is possible that the thickness of the electrolyte (ceramic, polymer, hybrid, gel), internal resistance, and temperature must be regulated according to the cell components used. In situ analytical tools enable us to observe and regulate the cell parameters during cell operation. However, the electromagnetic resistance of the electrodes and work safety are major concerns while using in situ analysis.

**Figure 7:**
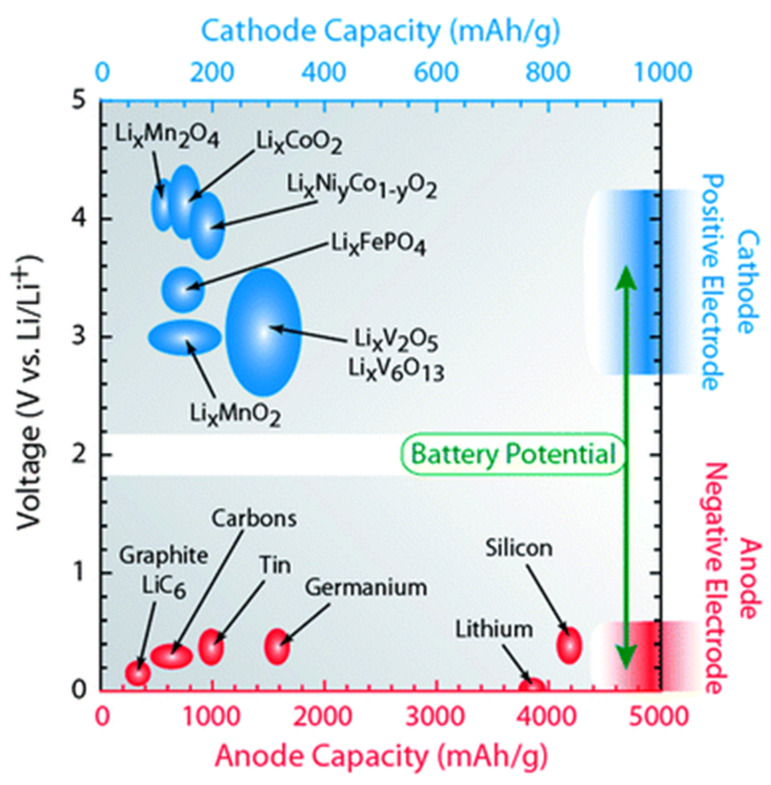
Specific capacities of common cathodes and anode materials in LIBs (adapted with permission from [158]. Energy Environ. Sci., 2009).

**Figure 8:**
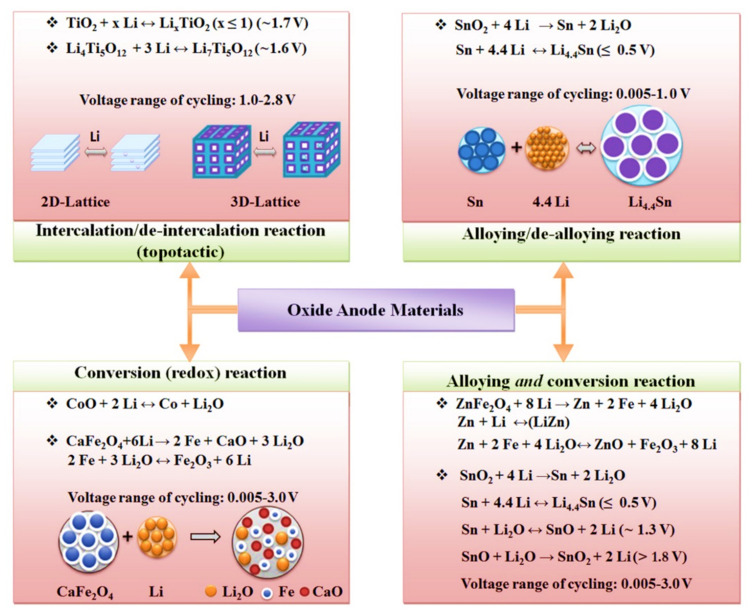
Reaction mechanisms of anode materials for Lithium batteries (adapted with permission from [133]. Chem Reviews, 2013).

**Figure 9:**
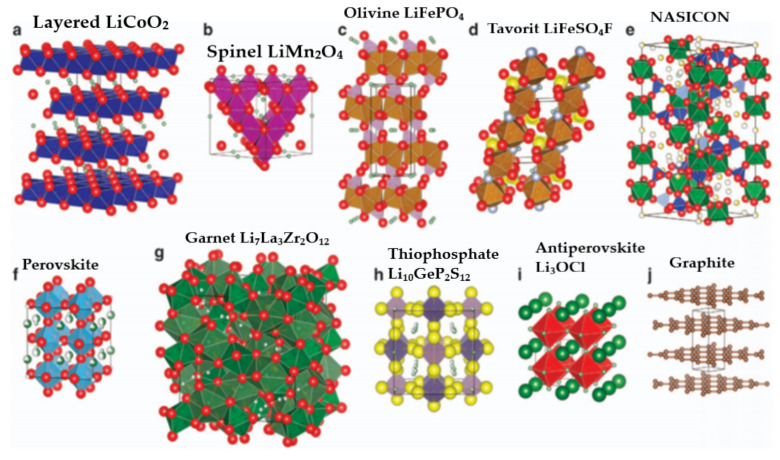
Different crystal structures of electrodes used in LIBs (adapted with permission from [159]. Nature, 2016).

As mentioned above, altering the electrode, electrolyte, and cell components influences the interfacial kinetics and the underlying electrochemical reactions, favoring or opposing dendrite growth. A comprehensive depiction of electrodes for LIBs with their specific capacity is shown in Figure 7, and possible reaction mechanisms are shown in Figure 8. These charts can aid in the selection of materials and facilitate an understanding of their possible mechanisms for the fabrication of LIBs. Moreover, the structure of electrode components is also important for envisaging the reaction kinetics and bonding of electrochemical species in a cell. Therefore, different types of electrode structures of LIBs are shown in Figure 9.

### 2.2. Materials for Na-Based Batteries

**Figure 10:**
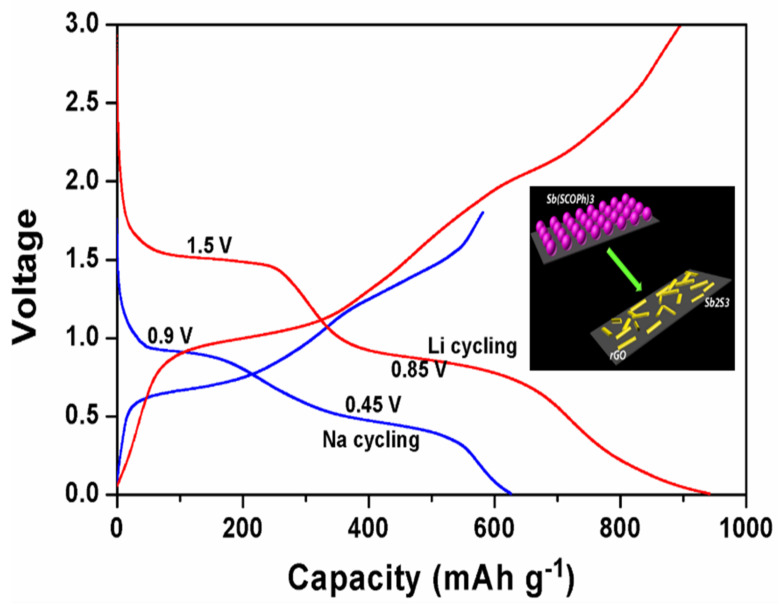
Charge–discharge studies of Rgo/Sb_2_S_3_ nanocomposites with lithium and sodium anodes (adapted with permission from [162]. ACS, 2016).

Na is widely available and has potential benefits for future grid storage. Similar to lithium-ion batteries, Na-ion batteries (NIBs) share the same guest–host intercalation mechanism using a hard carbon anode. NIBs deliver ~300 mAh g^−1^ with a potential above ~0.20 V Vs Na/Na^+^ [160]. Hard carbon, alloys, sulfides, oxides, and organic compounds have been examined as anode materials in Na-based batteries. Hard carbon treated at temperatures above 1500 °C manifests a specific capacity of 330 mAh g^−1^ at low operating potential (0–0.25 V). Higher operating potential or fast charging may result in Na deposition over the anode (hard carbon) surface, leading to blaze and a reduction in columbic efficiency. In comparison, graphite can be used as an anode for Na batteries in ether-based electrolytes through solvent intercalation. Alloys (Sb-C, Sn-Sb) offer high capacities ~600 mAh g^−1^, yet continuous cycles may cause a drastic volume change and electrode instability. Organic compounds such as Na_2_C_8_H_4_O_4_ as anodes are of low cost but exhibit poor electronic conductivity and cyclic stability. Sulfide compounds (TiS_2_ and MoS_2_) offer ~200 mAh g^−1^ but are sensitive to oxygen molecules [161]. The cyclic stability and charge–discharge performance of rGO/Sb_2_S_3_ was analyzed for Li- and Na-based electrodes [162]. The open-circuit voltage (OCV-3.5 V), cut-off voltage (COV-1.5 V), and discharge capacity (943 mAh g^−1^) of a Li-ion cell were higher than those of a Na-ion cell (OCV-1.8 V, COV-0.9 V, and discharge capacity (~620 mAh g^−1^)) as shown in Figure 10.

**Figure 11:**
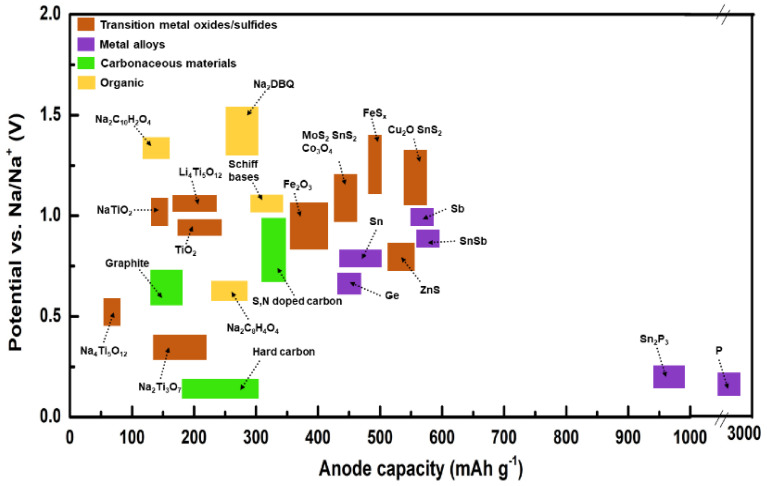
Specific capacities of anode materials used in Na-based batteries.

**Figure 12:**
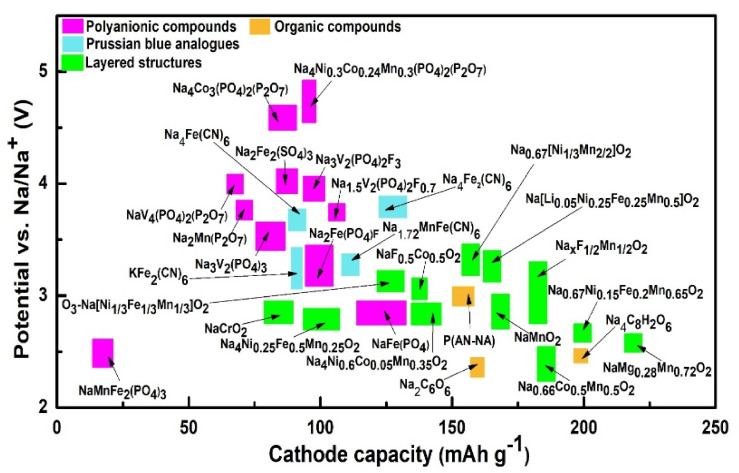
Specific capacities of cathode materials used in Na-based batteries.

Na_2_Ti_3_O_7_ and NaTiO_2_ were studied as electrode materials in Na-ion batteries, offering 100–180 mAh g^−1^ at low operating potentials. Substitution of Ti breaks up or fills the Na^+^ vacancy orders, resulting in better structural stability [163,164]. Recently, numerous materials have been adapted and studied as electrodes for Na-based batteries. Prussian blue undergoes two-electron reversible reactions during the insertion process due to its high specific capacity, and it has a highly cubic open framework, enhancing the ionic conductance, with minimal structural changes and tunable properties. However, these open structures release toxic cyanide during the electrochemical reaction and have low crystal density (1.96 g cm^−3^). Furthermore, water molecules and vacancy defects in Prussian blue trigger interstitial sites’ occupancy by zeolite water, thus reducing the diffusion of Na ions and the structural stability [165,166]. NASICON compounds (Na superionic 3D structures) have been primarily used with solid electrolytes [167,168]. Vanadium-based NASICON compounds have shown capacities of ~100 to 150 mAh g^−1^ under the operating potential of 2.5–4.1 V, yet these compounds have potential toxicity due to the release of fluorine gas [169]. A Na-O_2_ battery has better performance as Na^+^ ions have a large radius (greater than Li), leading to better interactions with O_2_^−^ ions. Similar to Li-based batteries, Na layers undergo repeated structural changes from octahedral to trigonal prismatic [81]. More research on Ti-based layered structures, sulfides, and NASICON compounds is required in order to achieve an excellent lifecycle and stability in Na-based batteries. Sodium battery electrodes, their specific capacity, and their operating potential are illustrated in Figure 11 and Figure 12.

### 2.3. Materials for K-Based Batteries

**Figure 13:**
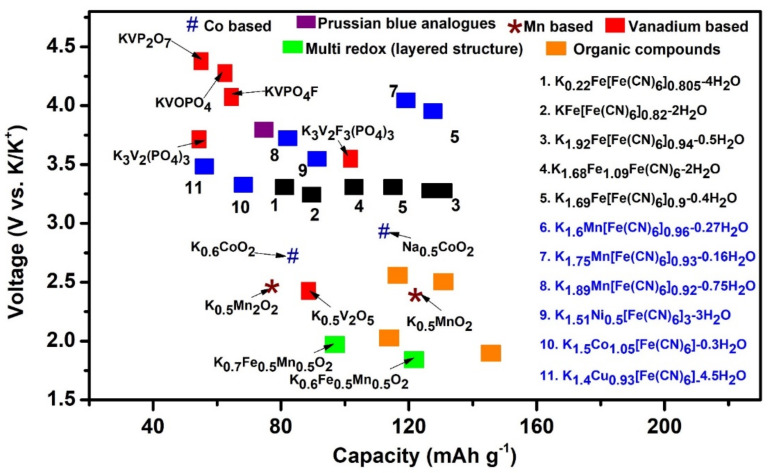
Specific capacities of electrode materials used in potassium-based batteries.

Potassium, a large-ion lithophile element, exhibits similar electrochemical properties, such as K^+^/K redox potentials, to lithium and is available at low cost [170]. The major challenge in K batteries is associated with framing ideal materials to insert large K^+^ ions. Prussian compounds, layered conversion metal oxides, and polyanions have been reported as electrode constituents for K-ion batteries [171]. Orthorhombic lepidocrocite structured minerals have been used in K-ion batteries. Diverse host materials, such as graphite, oxides, and sulfides (MoS_2_, SnS_2_), were identified as ideal insertion frameworks [172,173]. Recently, layered honeycomb structures (K_2_Ni_2_TeO_6_) have been found to exhibit high stability and ionic conductivity at an operating potential of 4V in potassium bis(trifluorosulfonly)imide, as shown in Figure 13 [174]. 

## 3. Mechanism of Dendrite Formation

**Figure 14:**
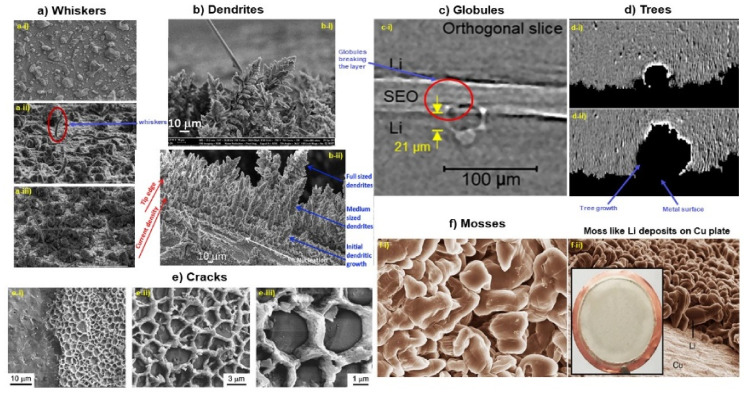
Different types of protrusions affecting battery performance: (**a**) whiskers (adapted with permission from [175]. J. Power Sources, Elsevier, 2011) (**b**) dendrites (adapted with permission from [176]. Acta Mater. 2017) (**c**) globules (adapted with permission from [177]. J. phys. Chem. C, 2018) (**d**) trees (adapted with permission from [178]. J. Power Sources, Elsevier, 2001) (**e**) cracks (adapted with permission from [179]. Electrochim Acta, Elsevier, 2017) (**f**) mosses (adapted with permission from [180]. Nat Commun, 2015).

It is widely known that the metal crystallization process (Ag, Fe, Cu) involves the formation of dendrites. Depending on the growth contingencies, dendritic structures differ in dimension, shape, and concentration. These dynamic conditions include the anodic surface morphology, depth of discharge, operating potential, concentration, diffusion kinetics of ions in the electrolyte, surface roughness, and current density. Various dendritic structures, such as moss, bush, tree, needle, and whiskers, have been reported in this context. The structure of these anodic protuberances is shown in Figure 14. Their dimensions and appearance are discussed in Table 3.

The mechanism and control of dendritic structures in aqueous electrolytes and a few non-aqueous systems are already known. However, the formation mechanism and control kinetics in various non-aqueous solvents are still under research. In general, three stages are involved in the mechanistic study of dendrites: (i) budding, (ii) nucleation, and (iii) growth [175,176,177,178,179,180]. Furthermore, to gain an in-depth understanding of control strategies, the dendrite growth mechanism and its related theory are discussed.

### 3.1. Dendrite Growth Theories

**Figure 15:**
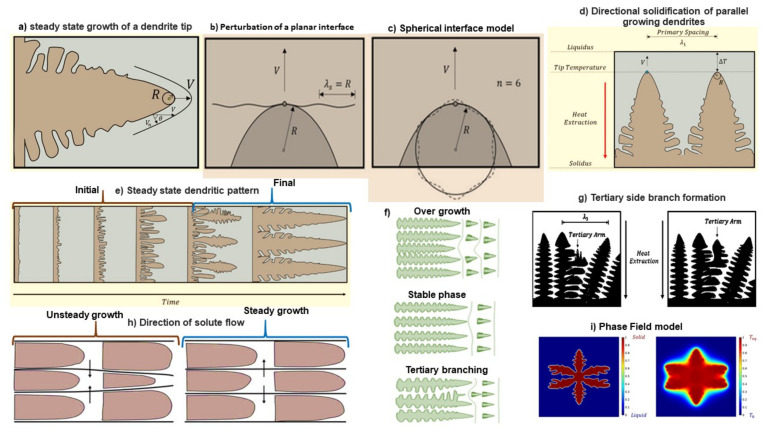
(**a**) Steady state growth mechanism of dendrite tip (**b**) Illustration of perturbation of planar surface (**c**) Depiction of dendrite growth according to (**d**) Directional solidification of dendrites (**e**) Steady state growth pattern (**f**) Stable, over growth dendrite depiction (**g**) Tertiary branching of dendrites (**h**) Solute flow direction for steady and unsteady growth (**i**) Phase field model simulation showing dendrite growth (adapted with permission from [181]. Crystals, MDPI, 2020).

The deposition characteristics and mechanism of alkali ions differ from the intercalation–extraction process in alkali-ion batteries. Dendritic formations are widely observed in the electrodeposition of metals (Zn, Ag, Cu, and Sn). Until now, several models have been developed describing the dendrite disposition and thickening mechanism. Dendrite structures constitute a significant threat to alkali metal batteries. Ultimately, in order to overcome this challenge, an in-depth understanding of dendrite growth is needed. In the subsequent section, a detailed analysis of the growth characteristics and mechanism of metallic dendrite formation is described in order to provide more clarity regarding the control strategies of dendrites in alkali batteries. Due to the complexities of analyzing aggregated dendrite structures, the steady-state growth model was initially centered on a single dendrite growth mechanism under the elimination of latent energy (i.e., constant-temperature system). A parabolic (V-shaped) edge interphase was observed with continuous concentration profiles, as shown in Figure 15a. The mathematical equations are reported for 2D and 3D cases of isoconcentrate dendrite interface structures.

2D case, Ω_C_ = √πP_c_ exp^Pc^ (1 − erf √P_c_) (1)

3D case, Ω_C_ = πP_c_ exp^Pc^ E_1_ (P_c_) (2)

where Pc is the Peclét number, V is the velocity of growth, R is the radius of the dendrite apex, D is the solute diffusive rate, E_1_(P_c_) is the exponential integral factor, and Ω_C_ is the under-cooling rate of the solute [181]. A lithium polymer cell (LiTFSI electrolyte) was observed during the galvanostatic charge process, accommodating assumptions from this model. The acceleration of dendrites towards the cathode was reduced by controlling the current density. Short-circuiting occurred at current densities above 75% [182]. In general, the slow diffusion of dendrites limits cell collapse. Ivantsov related the concepts of isotropic and anisotropic growth models. According to this model, a dendrite’s surface is considered dimensionless, and parabolic dendrites are considered elliptical. Under isotropic conditions, the dendrite tip was identified to be plane and globular in shape, as shown in Figure 15b,c. In anisotropic conditions, deformation of the dendrite tip along the path of anisotropy was observed [182]. The results from this model satisfy the equation

VR = constant(3)

The Lagrangian particle-based method in anisotropic electrolytes promotes the annihilation of dendrites in Li-ion batteries [183]. However, no unique solution can be reached using this model, leading to the development of diffusion and capillary models. In a lamellar transformation, the crystal grows in the shape of a needle, extending its length (Figure 15d,e). These unidirectional needle structures are complex spatial functions of temporal patterns with characteristic λ when observed regularly (1D) to the growth direction. The typical length λ varies with temperature gradient, growth speed, melt flow, and alloy composition. The capillary model was used in prototyping the diffusion and capillary pathways. According to this model, capillary inclusion provides the maximum V–R curve, and phase changes in structures occur at the highest growth rate, termed extreme conditions [184]. However, this model is not suitable for higher supersaturation conditions.

The space charge model has been widely used to analyze the nucleation and growth kinetics of alkali metal dendrites. This model unravels the formation of transport charges (ions) in a dilute electrolyte medium in association with diffusion and mobility parameters [185]. The model predicts the existence of a local space charge during the formation of dendrites. This space charge accumulates at the anodic surface due to the exhaustion of cations near the anodic surfaces at high current density, leading to wavering electroneutrality. Chazalviel affirms the absence of Li^+^ at high current densities on the electrode surface, allowing the unfettered formation of dendritic structures. He also expressed the initial time associated with dendrite progress as Sand’s time (τ) [186].

(4) $$\tau =\pi D\frac{eC_{0}{\left(\mu_{a}+\mu_{c}\right)}^{2}}{2J{\mu_{a}}^{2}}$$

where D is the coefficient of diffusion (ambipolar), e is the charge of the sub-atomic particle (electron), C_0_ is the original concentration of the electrolyte, J is the current density, and μ_a_, μ_c_ are anionic–cationic mobility. Thus, from the space charge model, it is clear that dendrite development could be repressed at low values of current densities and high ionic mobility. In addition, a higher Sand’s time delays the generation of dendrites and extends the time needed for consistent deposition [187].

On increasing the metal ion concentration in bulk electrolytes, uniform deposits accumulate on the anodic surface. Additionally, current density (J) must be minimal in order to achieve smooth SEI deposition. Increasing cationic transfer (μ_c_ and μ_a_) leads to ample Sand’s time, favoring uniform deposition and reducing the likelihood of dendrite formation. Ely and Garcia examined the early stages of dendrite formation from clusters. Using computational methods, the nucleation process of a dendrite was divided into the following phases: suppression phase, extensive incubation phase, rapid incubation phase, premature evolution phase, and development phase. The following predictions were made based on this model: (i) increased incubation time thermodynamically favors the nucleation and growth of embryos by dissolving it into the electrolyte medium; (ii) decreased incubation time enhances the growth and formation of stable alkali nuclei; (iii) regulation and control of nuclei embodiment can repress the growth of dendrites by forming planar structures rather than spikes [188]. For a better understanding, steadily grown dendrites, tertiary branching, and phase-field models are shown in Figure 15f–i. Dendrite growth from the initial unstable front to the final steady states is depicted depending on λ (Figure 15h). The phase-field model (Figure 15i) shows a complex interface and associated growth shapes at higher resolution with low anisotropic error. This model validates the microscopic solvability theory and is used in various applications [181]. According to mechanical stress and deposition models, dendrites are formed due to mechanical pressure and tension, which can be articulated in the form of the Laplace equation:

∆P = γ (1/R_1_ + 1/R_2_) (5)

Yamaki and his group demonstrated that sheets with rough surfaces (surface tension > 0.2 Nm^−1^) could be adapted as passivation layers above the alkali anode in order to inhibit dendrite formation. Another group related dendrite extension to residual stresses arising from alkali stripping. Chazalviel found that the momentum of the anion moving back from the SEI is proportional to the growth rate (G) of the dendrite in an applied field [188].

G = −μ_a_ E_bulk_ (6)

**Figure 16:**
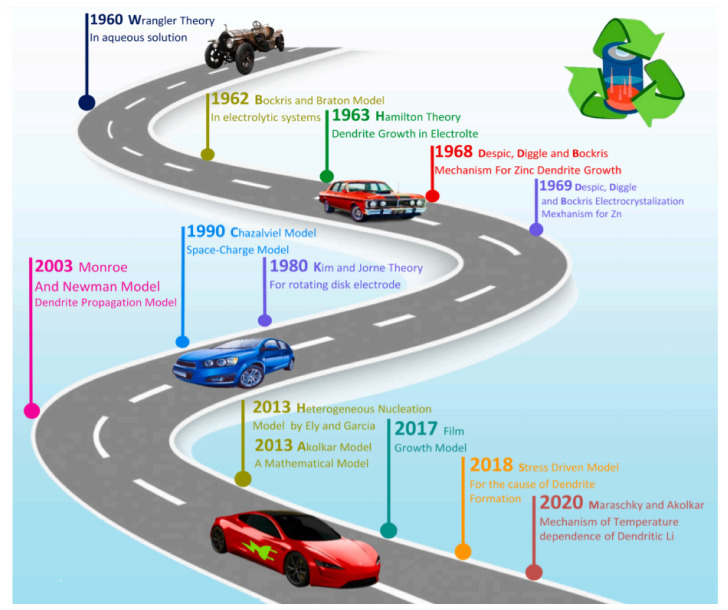
Roadmap of dendrite growth models, principles, and theories from 1960 to 2020 (adapted with permission from [192], Nano energy, Elsevier, 2021).

Newmann investigated protrusion development in constant current mode and inferred the dependence of molarity and voltage during charge–discharge cycles. The dendrite tip curvature (needles) radius is proportional to the growth velocity and non-proportional to the overpotential [186,188]. Density functional theory results reveal the predominant free energy changes between high- and low-dimensional Mg phases, which are higher than Li due to stronger bonding and lower diffusion barriers. Li is often prone to 1D dendrite growth, whereas dendrites on Mg preferably grow with high-dimensional morphologies. DFT theory revealed that Mg exhibits minimal diffusion barriers and higher interaction energy than Li and Na. This means that Mg ions are associated with higher power, creating diversified growth structures that favor high-dimensional anatomic dendrites. Self-diffusion is another crucial aspect in analyzing dendrite growth. According to DFT calculations, Li and Na have higher diffusion energies and are more prone to dendrite formation than Mg. However, in practice, in situ (electrolyte concentration, additives, anodic alloy) and ex situ (voltage, temperature, current density) factors contribute to dendrite growth. Instead of plain Na or Li, Na-Mg or Li-Mg alloy can be used to circumvent dendrites; in particular, Na-Mg alloy has excellent gravimetric (2210 mAh g^−1^) and volumetric (3830 mAh cm^−1^) capacities [189,190,191]. Various other models are available for predicting the dendrite growth mechanism in electrolytes. Wranglen, Kim, and Jorne’s model describes the current density factor and critical value above which the dendrite grows enormously. Barton and Bockris’s theory deals with Zn and Ag metal dendrite growth. However, they fail to consider effects on interfaces. Until now, researchers have suggested models based on mathematical and experimental assessments. The overall process of dendrite growth models in electrolytes is shown in Figure 16. The Akolkar model is the most recently introduced one, which mathematically and computational defines the dendrite growth velocity in relation to the surface and tip current densities [192]. This model was proposed based on galvanostatic observations of dendrite growth on alkali metals.

### 3.2. SEI Formation and Detrimental Effects of Dendrite Growth

**Figure 17:**
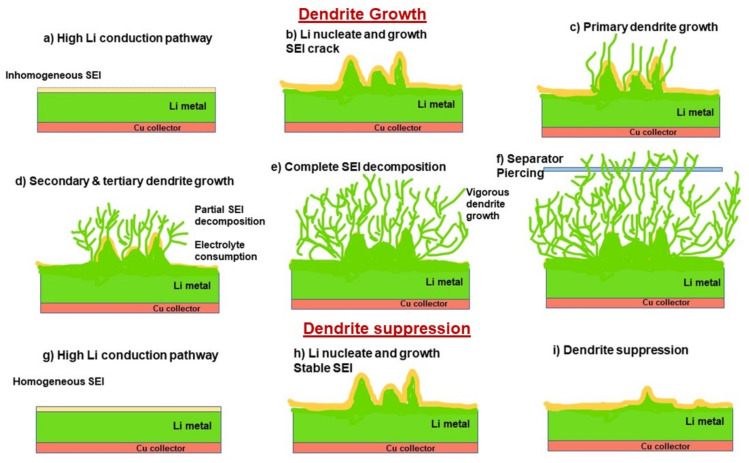
(**a**–**f**) Dendrite growth steps. (**g**–**i**) Suppression of dendrites due to stable SEI.

**Figure 18:**
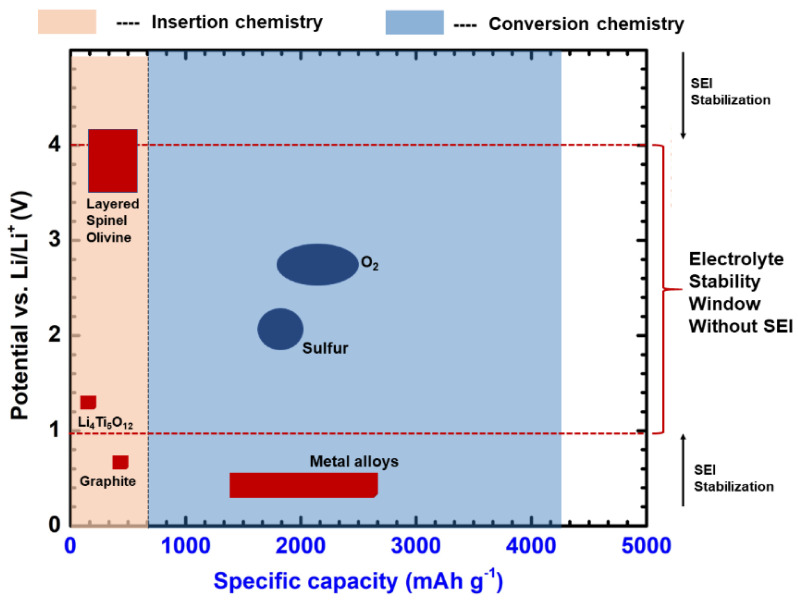
Specific capacity and SEI stability of Li cathodes.

Immersion of an alkali metal into an electrolyte medium with negative potential favors the reduction of salt ions in the electrolyte. These salt ions react with the alkali metal cations, forming an insoluble solid electrolyte interphase (SEI) and moderately soluble polymers and carbon compounds depending on the electrolyte composition [190]. The process of dendrite formation and stabilization is shown in Figure 17. The SEI formation and stability varies with the anode material, electrolyte species, current density, temperature, and sorting of cell components. Figure 18 shows the stability of SEI in relation to the electrochemical reactions and voltage window. Sulfur and O_2_ cathodes show superior specific capacities; however, steady SEI creation is mandatory. A perfect SEI must have high ionic conductance, regular thickness, high elastic modulus, and mechanical strength in order to prevent the further development of dendrites. In 1979, Peled identified an SEI and predicted its occurrence through surface reactions [193,194]. His observations are popularly known as the Peled model, in which the electrode–electrolyte interface reaction occurs gradually in steps through the reduction reaction of electrolyte species. The formed structure had several Schottky defects due to ion migration. Two conclusions can be drawn from the Peled model: (i) solvated cations cover the solvent species, resulting in internal Schottky defects; (ii) Li ions (cations) interact with the bulk of the SEI through the Schottky vacancies [194]. Later, mosaic-shaped structural deposits on the anode were observed, which laid the foundation for the mosaic model. The reduced species (similar to the Peled model) and insoluble multiphase deposits formed the SEI (shape of mosaic), permitting the cations (Li^+^) to drift through the polyphase deposits.

**Figure 19:**
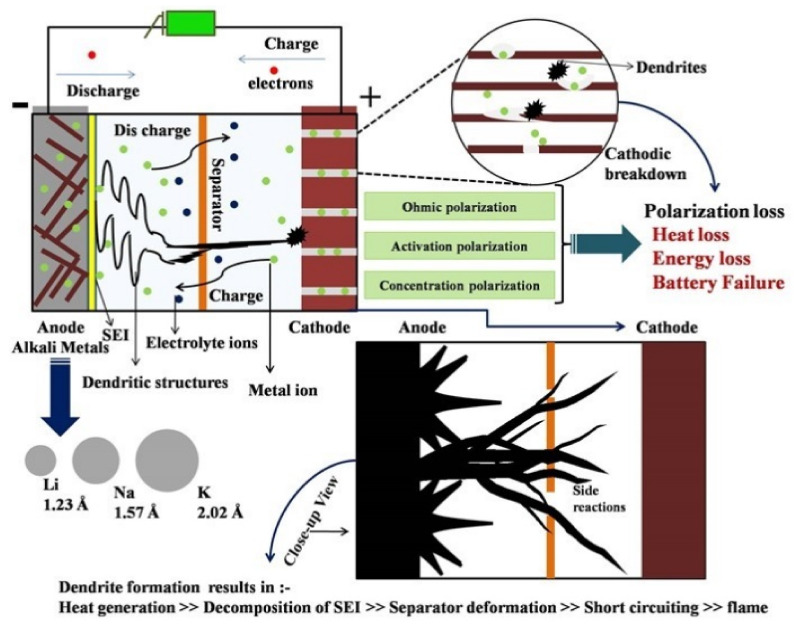
Vulnerable effects of dendrite growth in metal batteries.

Furthermore, coulombic charge interactions forming electric double layers have been reported based on other similar models, and these double-layer SEIs were relatively more rigid [194]. Additionally, the cation deposits must be in control, i.e., excessive cation deposition may cause the anode to become less involved in the redox reactions during the cycling process and increase the volume expansion. One of the major problems associated with battery breakdown is volume augmentation, which leads to deep cracks in the SEI, as shown in Figure 19 [195]. Volume expansion reduces the anodic surface energy barrier, intensifying non-uniform deposition. Furthermore, heterogeneous nucleation of lithium with increased nucleus curvature leads to high electric fields at the dendrite tip. This elevation attracts more cations, increasing dendrite growth and leading to battery collapse.

In LIBs, the electro-reduction potential of the electrolyte medium is below 1.0 V vs. Li/Li^+^. Even with the exposure of bare lithium to organic solvents, rapid interactions between Li^+^ and the electrolyte ions take place, forming insoluble by-products that are deposited onto the lithium surface. The components and the composition of the SEI layer vary with the electrolyte used and its concentration. The reaction rate occurring at the electrode–electrolyte interface depends on the velocity and ionic concentrations. Most of the inorganic decomposition products in Li-based batteries that deposit as the SEI are LiF, Li_2_O, Li_2_CO_3_, LiCl, LiOH, and organic groups such as POLi, PCOOLi, POCOLi, PCOO_2_Li, and POCO_2_Li; P = alkyl groups [196,197]. A mixture of decomposition products is formed because the electrolytic solution in alkali batteries contains a combination of chemicals to satisfy the following parameters: (i) high ionic conductance, (ii) low viscosity, (iii) high stability, and (iv) low flammability. As mentioned previously, depending upon the components of the electrolyte, the SEI composition varies. For example, while using LiPF_6_ salt with organic carbonate as the electrolytes, the majority of the SEI film contains LiF and PF_5_ [197]. While using non-combinational EC, C_2_H_5_COOLi and lithium carbonate (Li_2_CO_3_) were found to be the main components of SEI. The smoothness and uniform depositions of the SEI improve with EC/PC in dioxolane (DOL), especially with sulfur cathodes. Jingling Zhou and co-workers reported high coulombic efficiency without dendrites using LiFSI and dibutyl ether (DBE) in Li–sulfur batteries [198]. Dendrite growth can also be controlled by altering the external and internal cell parameters, such as temperature, pressure, surface energy, and morphology.

### 3.3. Influential Factors for Dendrite Growth

Temperature and current density play an essential role in dendrite growth and SEI formation. Ishikawa’s group revealed stable SEI formation with a graphite anode with high cyclic performance at −20 °C owing to the reduced insertion of solvents at the anode and meager dissolution of the SEI. At high temperatures, premature SEI can quickly decompose without complete formation; therefore, a moderate-temperature treatment (30–60 °C) is mandatory to ensure the adhesion of the SEI to the anode. After the regular formation of the SEI, subjecting the formed surface to high temperatures results in better efficiency without dendrites, lessening the polysulfide shuttle in sulfur batteries [199]. Dolle, at low current densities, observed organic and inorganic components in the SEI and only Li_2_CO_3_ (inorganic) at high current densities through SEM-EDS [200]. Similar to a Li-cell (space charge model) at low current density, adjusting the interfacial elastic strength and enhancing the Na^+^ mobility was found to control the growth of dendrites in Na-O_2_ batteries [201]. Various thermodynamic parameters have been investigated by researchers for detecting the dendritic growth mechanism. Classical film growth theory suggests that changes in the surface energy of the SEI in a homogeneous deposition process favor island-type dendrite growth. As the surface energy increases, the deposited film surface becomes rough, reducing the coulombic efficiency, which can be related to Young’s equation [202]. Increased potential can aggravate dendrite growth in organic electrolytes due to fast ion transfer and deposition. Field intensity (phase field) is an additional parameter influencing dendrite growth. Mathematical models, particularly finite element methods, reveal a diffusive solid–liquid interface with a specific diffusion coefficient, concentrations of salt, and conductivities [202]. Poisson equations have been employed to identify the theoretical viscosity, electrostatic potential, and phase field. Various linear and non-linear Butler–Volmer kinetic equations have been used to study the space charge interface accumulations [203,204]. Mathematical models reveal the proportionality of the tip radius of deposits to the square root of growth velocity. The contact angle and degree of adhesion of the SEI also play a significant role in dendritic suppression [205]. More detailed investigations in surface science and interfacial physics might generate a breakthrough solution for complete dendrite clearance in battery technology. Mu et al. reported the control of dendrites while increasing the voltage, as simulated through a phase-field model. The voltage influenced the overpotential, increasing the electrochemical reaction and affecting the dendrite growth rate [206].

An ideal SEI must prevent the interaction between electrons from the electrode and the electrolyte in order to avoid electrolyte reduction. There are three ways to achieve this prevention: (i) blocking the transport of electrons from the electrode (via leakage of other electrons or tunneling); (ii) blocking the electrolyte species or Li^+^ ions through the SEI; (iii) chemically and mechanically stabilizing the SEI. The mechanical strength of the SEI, such as elasticity, toughness, and adhesiveness, must be monitored in order to maintain the compactness of the SEI during the lithiation–delithiation process [207]. The layer must undergo elastic rather than plastic (permanent, irreversible) deformation. These characteristics depend upon the components of the SEI. The stiffness of various compounds was predicted using computational simulations and DFT. LiF is comparatively stiffer than Li_2_CO_3_, Li_2_EDC, LiMC, and PEO. The stiffness decreased from inorganic to organic (polymer), and the (001) planes of Li_2_CO_3_ adhere intact to the graphite electrode, with a force of adhesion of 1.86 J/m^2^ [208]. Other DFT simulations showed that oligomers attach steadily to the Li_3_Si_4_ (010) surface [207]. Furthermore, the irreversible capacity loss (capacity fading during the initial charge cycle) must be lower in order to achieve stiffer adhesion. The chemistry behind such bonding may lead to an explicit understanding of this phenomenon. The irreversible capacity loss is directly proportional to the explicit surface area of the anode in the formation of Li_2_CO_3_ films [208]. 

High conductance of cations with sufficient electrical resistance accompanied by firm SEI requirements, such as mild thickness (~nm), mechanical toughness (i.e., strength + ductility), high resistance to volume variations, electrolyte insolubility, and thermal stability, favors improved battery performance. Compared to Li^+^ ions, Na and K ions develop improper interface layers due to their instant reactivity. Na and K have weaker Lewis acidity and larger ion sizes, which leads to the formation of the SEI at open-circuit potentials with adverse reactions [209]. In comparison, Si- or carbon-based anodes do not form a passivation film even at open-circuit voltage (OCV) and undergo lithiation below 1.0 V. The severe reactions in Na or K metals result in improper deposition at the interfacial layer. In addition, metal anodes are generally more reactive than cathodes (graphite or carbon) and are susceptible to major volume transformations [210].

**Figure 20:**
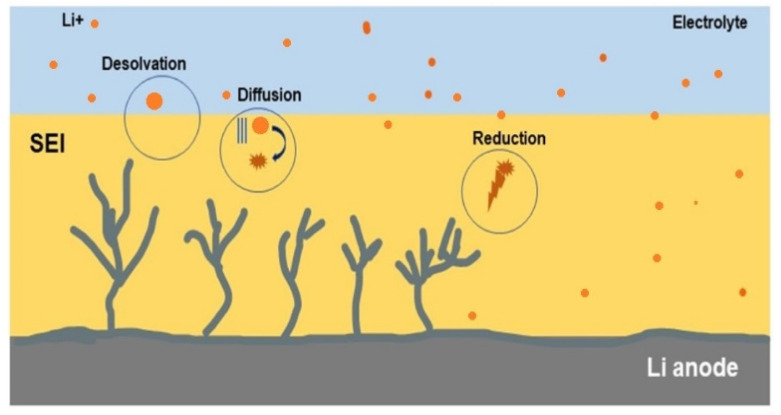
Schematic representation of dendrite growth reactions.

During the first cycle, the new solution upon interaction with the anode undergoes a reduction process at low selectivity. However, once the reacted species form precipitated structures over the surface, anodic contact is blocked. Further reduction of electrolytic species after precipitation occurs at high selectivity, degrading the well-built passivation layer with recurring precipitates. This recurring precipitate contains insoluble and half-soluble reduction products of electrolytic components with the host metal. The solvated metal ions form an electric double layer with high content of reduced species from the electrolyte in the inner layer. The internal layer content becomes richer in metal ions upon a saturation voltage point (~2 V in LIB) than reduced electrolyte species, forming a thin passivation layer [211]. Therefore, the SEI is not a single layer but rather a multilayered structure with an inner metal-oxide layer, intermediate reactive layer (reactive species F^−^), and outer organic layer for electrolyte interaction. If the electrolyte is highly concentrated with salt ions, then vigorous chemical reactions occur in the cell to reach the optimum voltage point. The solvated ions dissolve, diffuse, and reduce the species in the electrolyte, as shown in Figure 20.

**Figure 21:**
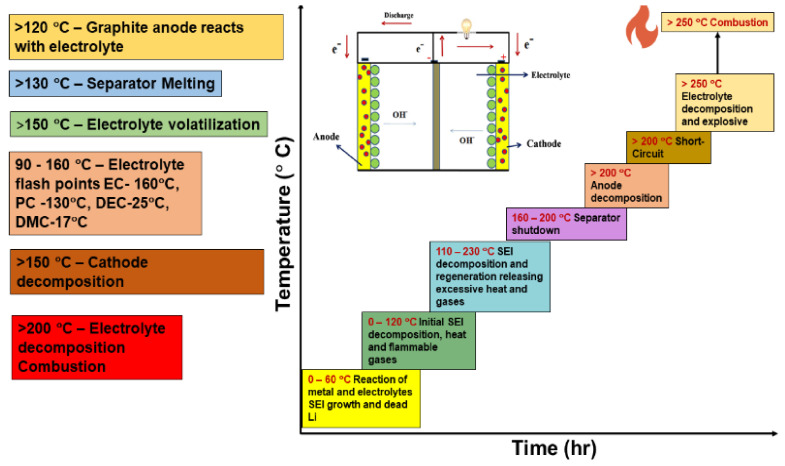
Hazards related to thermal instability due to dendrite growth.

After the saturated voltage point, even a single pulse with high current density may heat the multilayered structures (dendrites) and cause them to self-diffuse, reducing the branch formation. Self-diffusion causes the dendrite to flatten upon optimum heat generation without melting [212]. At high overpotentials, excessive heating of dendrites creates a localized temperature increase and triggers excessive ion diffusion. Reducing the inter-electrode spacing up to a specific range (based on the morphology and thickness) increases ion transport and the reaction rate. Sometimes, bringing the electrodes too close together may increase the concentration polarization and cause rapid flammability. The appropriate combination of electrolyte properties and field strength between the electrodes influences the ion transport concerning the voltage applied. Formed interphase structures greatly influence the anodic surface energy and polarization. The growth of the SEI depends on secondary side reactions with the electrolyte, hydration of surface molecules, diffusion of water (wettability), dissolution of compounds, reduction of electrolyte species, and the generated heat [212,213]. The hazards and effects of dendrite growth relative to thermal instability are pictorially represented in Figure 21.

### 3.4. Influence of Interfacial Viscosity and Crystal Solidification

In the last few decades, various developments have been made to examine the physical phenomena behind dendrite formation. We investigated different adsorption kinetics and heterogeneous surface science concepts in order to predict dendrite growth. The velocity of ions influences the reaction rate and dendrite outburst. At low speeds, the reaction rate will be minimal; thereby, the battery delivers less energy. On the other hand, high velocities result in a greater possibility of forming protrusions, disrupting the SEI. The surface science and kinetics behind the reactions must be adjusted to match the circumstances, especially in the material design aspect, in order to prevent battery failure [92,214]. According to the capillary hypothesis, maximum velocity exists at the curved tip of the dendrite due to the high surface energy and conduction at the end. As the dendrites grow at the tip with increased ion velocity, the nucleation increases, forming new branches upon solidification [92]. Combining transport theory and surface morphological firmness seems to predict the features of dendrite growth. Other factors, such as interfacial stability, entropy (minimal), marginal steadiness (assessing the dendrite tip radius), the viscosity of the electrolyte, and interfacial energy, must be taken into account [214]. Thus far, the causes, growth mechanism, and detrimental effects of dendrite growth have been discussed. Next, it is important to consider the tracking and monitoring of dendrite growth in order to devise plausible solutions based on their growth parameters. The forthcoming section deals with the most commonly employed characterization tools for the analysis and monitoring of dendrite growth.

## 4. Analytical Tools for SEI and Dendrite Assessment

**Figure 22:**
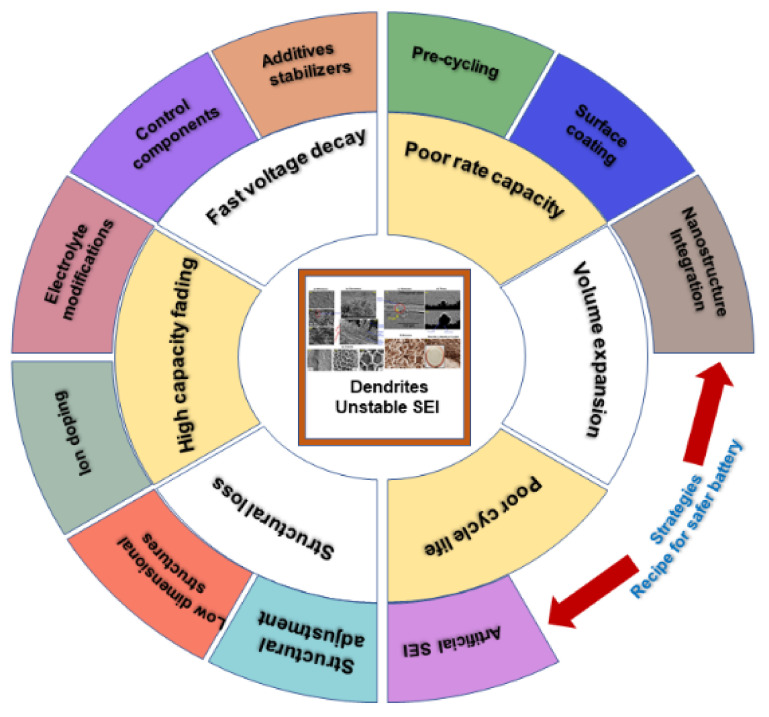
Challenges and strategies for dendrite elimination and growth regulation.

**Figure 23:**
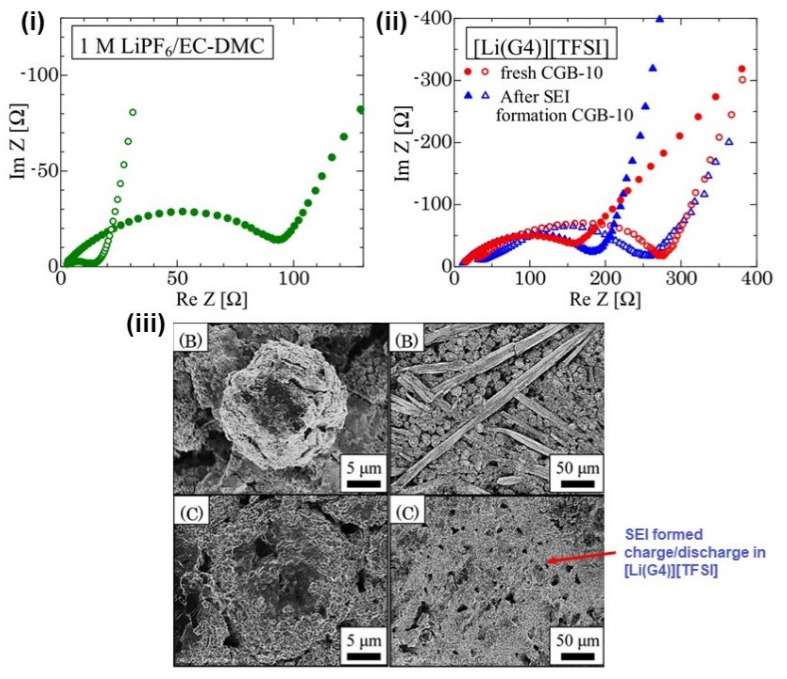
(**i**,**ii**) Impedance spectra of Li anode immersed in LiPF_6_ and LiTFSI electrolytes. (**iii**) Microscopic images of formed SEI (B) Graphite modified [Li(G4)][TFSI] surface after charge-discharge cycles (C) Formed SEI on [Li(G4)][TFSI] (adapted with permission from [216]. J. Electrochem Soc., 2018).

The SEI is the key parameter in a battery system that determines the safety, cycling capabilities, durability, and energy density. Some of the approaches to control the impairment caused by the dendrites, as discussed in Section 3, are shown in Figure 22. SEI formation begins with polarization at the cathode during the first few charging cycles, leading to the intercalation of Li^+^ solvated ions onto the anode. During this process, two main problems occur: (i) polarization losses and (ii) local expansion of the anode lattice, related to its crystallographic axes. Regarding this concern, analytical tools such as microscopes and electrochemical tools are useful for investigating the formation of the SEI. An example of an SEM image of solvated ions in graphite forming the SEI is shown in Figure 23iii. It can be inferred from Figure 23i,ii that the charge transfer resistance (Rct) of the anode is related to two activation processes: (i) desolvation of Li^+^ ions and (ii) its transition through the SEI layer. The cation transport number (total electrons carried in an electrolyte by cations) must approach one in order to eliminate the effects of the polarization concentration [215,216]. In alkali metal batteries, the equivalent volume of SEI material should be higher than the metal anode’s volume in order to achieve better protection [217]. Furthermore, corrosion may occur if the SEI layer has more pores and surplus O_2_^−^ ions (metal–O_2_ battery). Ultimately, the SEI must satisfy the following criteria: (i) high electron resistance and cation selectivity, (ii) cation permeability, (iii) strength and tolerance to stresses, (iv) insolubility in medium, (v) voltage stability. The SEI formation voltage depends on the anode structure, catalytic property, crystal orientation, temperature, concentration, level of impurities, and current density. 

The reduction potentials of Li, Na, and K are negative compared to the first free electron (solvated electron) in ammonia-based solutions [218]. The solvated electron is free (smallest anion) formed while dissolving ammonia, resulting in a blue tint. These solvated electrons diminish the solvent molecules if the SEI layer is not stable, creating self-discharge. For optimal results, the lifetime of the solvated ions must be lower [19,218]. Prolonged dissolution of ions results in battery breakdown, leading to uneven cracks and stress on the SEI. Quick breaks allow the electrolyte to move into the gap more quickly. Thus, new films (~1 μm) form on the anode surface due to the electrolyte interaction with the electrode surface, diminishing the corrosion and degradation of the anode. In the case of slow-spreading cracks, the electrolyte motion is restricted. Thus, no passivation layers are formed on the anode surface, resulting in more electrolytic degradation, which frequently occurs with Si anodes. The high speed of the formation of the deformed SEI increases the speed of the healing process, thus reducing the risk of battery failure. 

**Figure 24:**
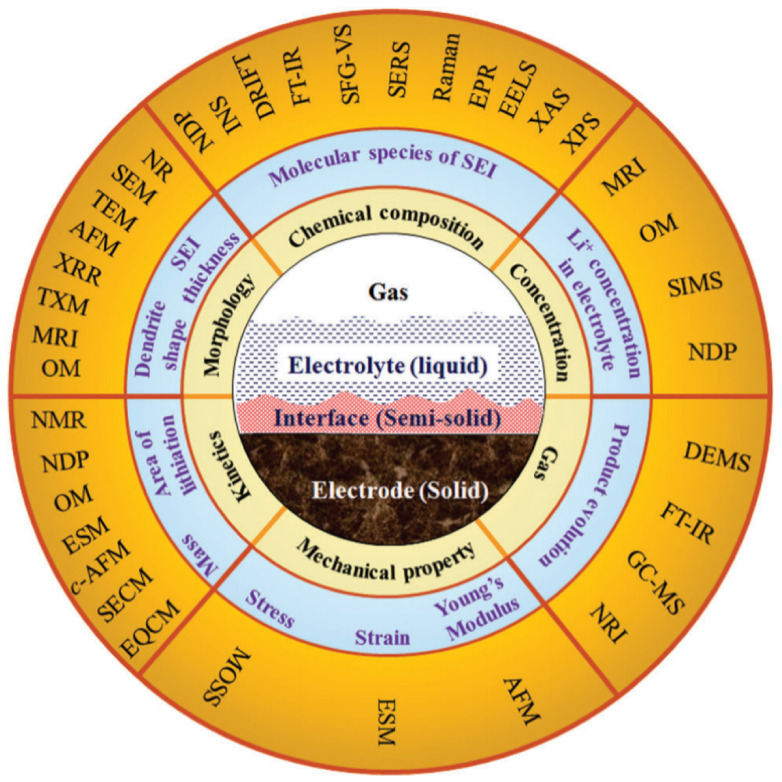
Analytical tools for analyzing electrode–electrolyte interface properties (adapted with permission from [219]. Chem Soc Rev, RSC, 2018).

The dendrite growth and SEI were analyzed using various electromagnetic rays outside the cell. These ex situ analyses do not provide real-time data for mathematical modeling. Besides ex situ analysis, in situ observations of dendrite growth characteristics are becoming vital for understanding the real-time, fast tracking of dendrite growth. In this context, scattering X-rays and electrons in operando contributes to identifying electronic, chemical, and dimensional changes in the electro substrate. The intercalation–deintercalation process results in alterations in lattice sites and, at times, leads to phase changes. X-ray-based tools are non-destructive and can detect the lattice parameters in both crystalline and amorphous SEI layers. Some of the pre-checks for analyzing a battery for in situ analysis are (i) proper space between electrodes, (ii) electrolyte concentration and quantity, (iii) pressure maintenance, (iv) proper cell sealing, (v) X-ray penetrative material or X-ray transport membrane, and (vi) anti-corrosive electrolyte. The primary concern with in situ X-ray analyses is the absorption and scattering of X-rays by non-targeted elements such as a separator, electrolyte, case, collector, etc., reducing the precision and data clarity [219]. Another drawback is the need for knowledge about the SEI’s organic material and its lattice parameters, which may not be available in the standard X-ray data cards. High-energy X-ray absorption and reflectometry can be used if the electrode material is undamaged by the intensity of X-rays. Electron spectroscopy for chemical analysis or X-ray photoelectron spectroscopy (XPS) have been used to understand the SEI kinetics in solid electrolytes by applying electrical forces [220]. In operando techniques are believed to bridge the gap between experimental and computational simulations by providing electrode–electrolyte interfacial information. Here, the design of cells appropriate for in operando assessment plays a significant role in increasing accuracy. Pouch cell designs are less able to withstand the high accumulation of pressure; as a result, the contact between cell components becomes poor and the cell’s internal resistance increases. The temperature stability of pouch cell materials such as polyethylene and polyester is also limited and they fail to provide support for more than one week. Aluminum-coated bags and a swage-lock setup can withstand high temperature and pressure, but, as they are non-transparent, the penetration of rays is a challenge. Designing a swage-cell structure with materials possessing high transparency and temperature–pressure stability may increase the reliability of in situ techniques. Hence, precise cell design and analytical tool selection are of high significance in the electrochemical interface research domain. One recent example is the investigation of the growth of dendrites via in situ scanning electron microscopy coupled with EDS, performed by the group of Karim [221]. The in situ study unveiled the dendrites’ hollow morphology and carbide content, with applied pressure suppressing the dendritic growth. Table 4 and Figure 24 show some of the in situ techniques reported in earlier studies.

## 5. Design Strategies Based on Growth Mechanism and Theoretical Models

### 5.1. Control of Pressure and Temperature

Cylindrical button cells are popular battery pack designs that suffer from excessive compressive pressure due to the steel casing and soft packing. External factors such as battery packing pressure and temperature play a role in improving the battery’s performance. The phase-field model shows potential for analyzing the influence of external pressure on dendrite deposition. Based on the phase-field model, Zhang et al. simulated the force and pressure impact on Li-Cu cells [238]. They inferred that metal deposition induces intrinsic strain and stress, which is altered by mechanical forces. The strain and mechanical forces result in the rearrangement of ions. It has been proposed that an increase in outside pressure leads to flat dendrites with minimal branches. Due to the high compression state of stress, the localized hydrostatic pressure in the dendrites shifts to a positive value and blocks dendrite branching and abnormal growth [238]. However, a force beyond a critical material load-bearing state has the possibility of causing terminal breakdown. Von Mises criteria of yield stress can be used to determine the fracture and deformation stress. Applying pressure to the correct area externally over the battery pack is vital for minimizing dendrite growth [238].

At times, concentrated pressure on one side of the cell may lead to damage to specific components. The optimum external pressure is 6 Mpa for a cell material with a Young’s modulus between 0.6 and 2 GPA [239]. Regulating dendrites through pressure control works well in electrodes when the elastic modulus of the electrolyte is minimal. Chan et al. used pressure to control the dimensions and shape of dendrites [239]. Pressure of 10 Mpa caused the reshaping of dendrites with smoother and flat edges, reducing the residual pores in a precycled lithium anode. However, external force factors vary for each material, and an extensive understanding of the mechanical parameters of cell components is required. Precycled lithium with dendrites has been tested chiefly with external pressure under working conditions [240].

Altering the temperature has a significant effect on the growth of dendrites. The coarse-grained Monte Carlo model proposes that, at low temperatures, the development of dendrites becomes severe. This vast growth occurs because more grains with a lower nucleation radius result in a densely packed nucleus [241]. Moreover, at low temperatures, the SEI comprises an amorphous structure, especially in polymer electrolytes, that can easily dissolve in liquid electrolytes upon cell cycling. As the SEI dissolves, the consumption of active electrodes occurs with undesired side reactions, leading to less anodic utilization. Arrhenius’s hypothesis also supports the notion that a high temperature (50–60 °C) favors a larger nucleus radius with low overpotential [242].

### 5.2. Current Density

The classical growth model coincides with the experimental results obtained in the nucleation and Li growth process proposed by Yi Cui et al. [243]. The results indicate that the overpotential, current density, and size of the nucleus influence each other. In order to verify the effect of current density on dendrite growth, in operando SEM images were taken, which revealed the proportionality between dendrite growth and current density. Recently, the current density at the electrodes was measured, setting the equilibrium potential, called the exchange current density, which was also proportional to dendrite growth [244]. Multiple parameters must be considered when formulating mathematical models to achieve smooth dendrite deposition. For instance, variable current density, temperature, and pressure can be assessed by in situ analytical tools during precycling and regulated accordingly, which will improve the model’s real-time performance [242].

### 5.3. Electrolyte Design

With an electric field, if applied uniformly, the dendrite growth can be controlled by regularizing the morphology. As the application of a homogeneous field accelerates ion diffusion and mechanical force against dendrite growth, the sharp tip of the dendrites becomes bent and round. A multi-component or multi-content electrolyte has greater potential for the synergic control of dendrites due to its mechanical and electrical durability against them. The concentration of each chemical in the electrolyte and the additive amount also influences the dendrite growth [245,246]. For instance, halogen and carbon-matrix additives act as a lithophilic buffer, accelerating the ion flux with the rigid framework of the SEI, sealing the dendrites when used at an optimum concentration [245]. Another example is trimethyl phosphate solvent, which generates an extremely sturdy SEI when used at a 5 M concentration with LiFSI salt. Here, at the particular 5 M concentration, the solvation structure and inter-reaction with the electrolyte and Li ions improve the framing of the tightly packed, solid SEI film structure. Similarly, Na dendrites are restricted by the use of NaTFSI in 2 M polyethylene carbonate/fluoroethylene carbonate [247].

The type of electrolyte, either liquid, polymer, or ceramic, also influences the dendrite growth morphology based on Barton and Bockris’s speculations since the SEI’s ion diffusion and mechanical stability differ under each electrolyte type [248]. It has been proposed that needles or whiskers develop predominantly in liquid electrolytes at low current density and low elastic modulus [248]. Mossy structures are observed in coin cells at the current density normalized to 10^−3^. The simultaneous increase in current density generates hybrid mossy–whisker structures that exist together, leading to more prolific dendrite growth [212]. Molten lithium was fused into an electrospun polymer matrix sealed by an outer zinc oxide layer, which exhibited porous, stable deposition of Li during the cyclic process, hindering dendrite outgrowth [249]. A composite of nickel and Li led to a dimensional alteration of the electrode, offering protection from dendrites [250].

### 5.4. Electrode and Interphase Modification

**Figure 25:**
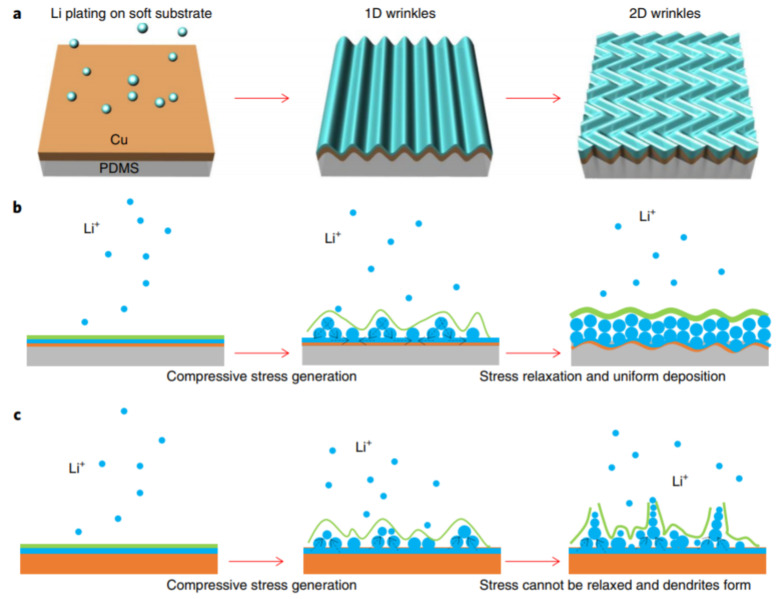
(**a**) Schematic view of wrinkle formation based on substrate types (**b**) Generation and relaxation of compressive stress in Li-Cu soft substrates (**c**) Formation of dendrites and relaxation of compressive stress in Li-Cu hard substrate (adapted with permission from [251]. Nat. Energy, Springer Nature, 2018).

Reinforcement of stress brings mechanical and microstructural changes to control dendrite growth, in line with the stress-driven dendrite growth model. One proposed example is the growth of whiskers as diminutive thin layers under compressive strain relaxation. Zeng et al. revealed the use of a thin-film Cu collector bolstered by soft substrates for relaxing the localized stress through wrinkling [251]. The smooth 3D substrates help to lessen the compressive pressure at the Li surface and prevent dendrite formation by eliminating further sharp deposition, resulting in 1D dendrite growth. Figure 25a,b show the stress application and relaxation in soft substrates developing 1D to 2D wrinkles with dendrite inhibition, whereas, in a hard substrate, as shown in Figure 25c, the compressive stress becomes concentrated and cannot be relaxed, leading to dendrite deposition. Thus, relaxing the stress changes the dimensionality and mitigates dendrite growth, which is in line with the stress-driven dendrite growth model [251]. A Li-Cu electrode in LiFePO_4_ exhibited high coulombic efficiency of 98%. Increasing the Sand’s time should mitigate dendrite growth according to space charge and heterogeneous deposition models [204], unless the thermodynamics and external parameters do not affect the system. Relieving the stress and mechanically driven control of dendrites have become important research pursuits.

## 6. Dendrite Control Strategies

### 6.1. Strategies for LIB Design and Development with Dendrite-Free Structures

#### 6.1.1. Electrolyte Solvent Modifications

The electrolyte is a requisite medium for ion dissolution and conduction, creating a solid–liquid or solid–solid interface in a battery system. Compared to a liquid electrolyte, a solid electrolyte hinders dendrite formation and improves safety. However, high interface impedance and high cost make solid electrolytes unreliable. In general, electrolytes incorporating lithium salt allow ion transfer to prevent electron conduction, acting as a bridge between the anode and cathode. In Li-ion batteries, Li salts (LiPF_6_, LiClO_4_, LiAsF_6_) are dissolved in organic solvents based on dipolar aprotic features (ethylene carbonate, diethyl carbonate, dimethoxymethane) to make up the electrolyte [252]. Salt of Li must contain certain features for building an extremely stable SEI, namely (i) good desolvation in solvents, (ii) formation of stable anions, (iii) thermal stability and insusceptibility to hydrolysis, (iv) high ion transport property, and (v) non-toxicity and abundance [253]. Linear-structured carbonic acids and cyclic-structured dialkyl and alkene carbonates and organic esters are used in LIBs. In commercial batteries, carbonate-based electrolytes such as dimethyl carbonate (DMC) (linear structure) and ethylene carbonate (EC) have been employed. The lithium salt electrolyte solution must display at least 0.001 to 0.005 S cm^−1^ of ionic conductivity at 20 °C. Lithium ethylene dicarbonate involves actively reducing Li^+^ ions in a single electron pathway, forming an SEI with the suggested properties [254]. EC has the major contribution in SEI formation and is linked to the limit of Li ion solvation by the molecules of EC. In contrast, linear carbonates (DMC) have a poor ability to solvate Li^+^ ions due to their linear structure. The drawback of PC is that they solvate ions with a reduction potential of ~0.8 V. This causes them to form an inhomogeneous and low-quality SEI. Moreover, the dielectric constant and polymer chain length are comparatively lower for PC. In particular, the corrosive effects damage the Al current collectors as Al is subjected to anodic polarization (>4 V) [255]. Moreover, the collector is extremely slim, meaning that corrosion may cause it to break down into scraps. To avoid this issue, effective passivation over the Al collector through surface coatings or retaining the formed Al_2_O_3_ layers has been adopted. Lithium propylene dicarbonate (LPDC) is similar to LEDC but cannot generate closely packed SEI structures because of the side methyl group. Li salts are responsible for electrolyte heating and inflammation. The nature of the anion (salt) governs the heat or combustive products produced in the electrolyte. For instance, linear carbonates are prone to a higher burning rate than cyclic structures. LiClO_4_ has good conductivity and passivates the Al collector. However, the oxidation state of chlorine is higher, making the cell prone to explosion at high temperatures [256,257]. LiAsF_6_ is thermally stable, with enhanced electrochemical stability compared to LiClO_4_. However, the toxicity of arsenic makes it unsuitable for widespread usage [257]. LiBF_4_ has low thermal stability, making it less suitable for battery applications. LiPF_6_ and LiCF_3_SO_3_ show high thermal and hydrolytic stability and form an intact SEI [256]. However, the selection of solvents makes the process complicated, as these salts exhibit low ionic conductance in carbonate solvents. The stability of common lithium salts is in the following order: LiFSI–LiBOB < LiPF_6_ < LiFSI–LiDFOB < LiTFSI–LiDFOB < LiTFSI–LiBOB [257]. These salts and solvents have been used since the 1990s, but new combinations of salt solvents providing higher energy density and capacity are urgently needed in order to satisfy current demands.

**Figure 26:**
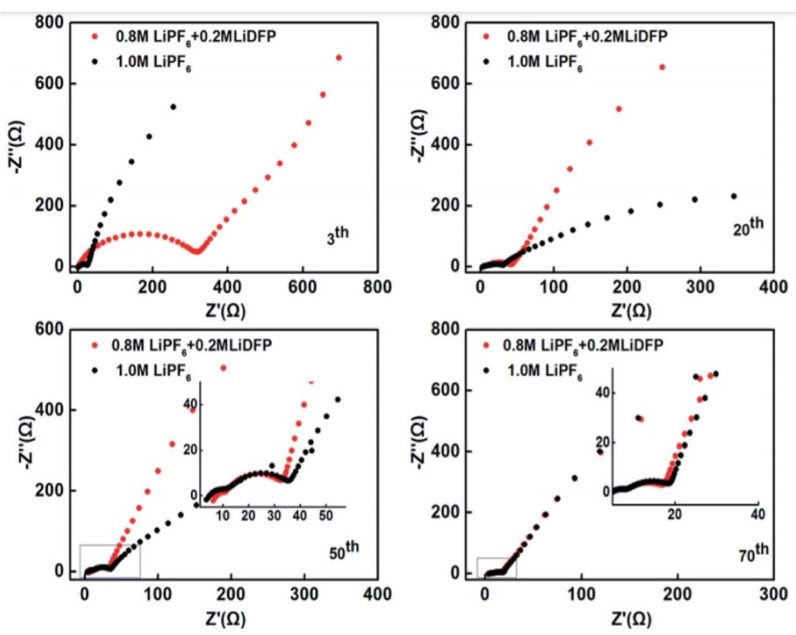
Electrochemical impedance analysis of Li anode LiDFP/LiPF_6_ dual salt electrolyte (adapted with permission from [258] RSC Adv., 2020).

Recently, Liu and co-workers designed a combinational electrolyte (LiDFP/LiPF_6_) dissolved in dimethoxy methane. LiPF_6_ solely decomposes at the potential of 4.24 V, and the addition of lithium fluorophosphate (LiDFP) raises the decomposition potential to ~4.5 V, revealing the enlarged electrochemical window of the dual salt electrolytic system [258]. Their cyclic study on half-cells with dissimilar molar ratios of LiDFP revealed a discharge capacity of 198.5 mAh g^−1^ after 120 cycles, with a capacity hold of 92%. Increased Li^+^ diffusion kinetics, stable cathode electrolyte interface (CEI) formation, and suppression of side reactions, enhancing the battery capacity and lifetime, were observed [258]. The high-frequency plot in Figure 26 demonstrates the interface impedance, which drastically raises after three cycles in both electrolytes (LiDFP/LiPF_6_) due to the existence of inorganic compounds in the passivation film, formed from the decomposition of electrolytic components. The mid-frequency charge transfer resistance plot shows the decrease in Li^+^ diffusion resistance after 20 and 40 cycles, demonstrating the augmented diffusion kinetics of Li ions, validated by the low-frequency impedance plot. Beyond 70 cycles, both the electrolytes present a similar decline in the curve, revealing a stable CEI film that actively lessens side reactions. Yifan Wu et al. analyzed the stable SEI formed over a Li_2_TiSiO_5_ anode during the first discharge process in a LiPF_6_ electrolyte. The formed SEI layer comprised mainly Li_2_CO_3_ and ROCOOLi, with trace amounts of LiF. The studies suggest that L_i2_TiSiO_5_ can be modified through surface coating to induce catalytic activity and stable SEI creation. Another group assessed the performance of 1.2 M LiPF_6_ in ethylene carbonate under various voltage conditions and found that the LiPF_6_ electrolyte decayed above 4.9 V [259]. 

Boron-based salts manifest high thermal stability, moderate conductance, and compatibility. Initially, lithium bis-(oxalato)borate (LiBOB) was found the possess the advantage of forming a stable SEI in PC with the standard reduction potential of 1.6 V vs. Li/Li^+^. Nonetheless, its poor performance at potentials above 4.1 V, poor solubility (<0.8 M), low ionic conductance, and high interfacial impedance with the anode restrict its usage. Lithium difluorooxalatoborate (LiDFOB) counters the disadvantages of LiBOB, with good thermal stability (~270 °C) and decomposition potential up to 4.3 V (0.5 V higher than LiPF_6_). LiDFOB improves the electrochemical performance of LiCoPO_4_ cathodes by fitting unimpaired SEI layers, suppressing dendritic growth at low voltages. However, at high voltages, the notion fails. Optimizing electrolyte formation and the use of anodic protective coatings may improve the electrochemical performance at high voltages, inhibiting the growth of dendrites [260]. Another exciting option is dilithium dodeco-flurododecarborate (Li_2_DFB). This salt is thermally stable up to 400 °C and its high ionic radius reduces the ion-pairing energy, resulting in high solubility [261]. Li_2_B_12_F_12_ has shown stable electrochemical performance up to 4.5 V and generates redox shuttles, preventing the Li overcharge potentials. A Li_2_B_12_F_12-x_H_x_-based electrolyte with appropriate additives was found to form a stable SEI over a graphite anode at 60 °C. The SEI remained 70% undamaged after 1200 cycles [262]. Xu et al. demonstrated the strategy of involving additives for enhanced SEI protection. They used tris(hexafluoro-iso-propyl)phosphate (HiFP) as a caping agent in EC and a spinel LiNi_0_._5_Mn_1.5_O_4_ anode. This agent promoted the stability of the electrolyte and SEI. Another study with a Li-rich manganese-coated LiNi_0.5_Co_0.2_Mn_0.3_O_2_ cathode in LiPF_6_ revealed high Li diffusion rates, excellent cyclic performance, and good SEI protection [263]. 

**Figure 27:**
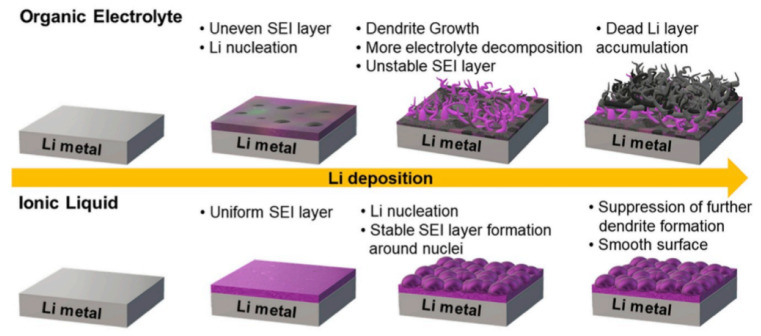
Comparative illustration of Li dendrite deposition in organic and ionic electrolyte (adapted with permission from [267]. Elsevier, 2020).

Lithium bi(fluorosulfonylimide) has gained attention due to its outstanding properties, such as high solubility and ionic conductance compared than LiPF_6_. However, LIFSI corrodes the Al collector at high voltages (>3.3 V vs. Li/Li^+^), forming Al(SFI)_3_ and diminishing the cycle life. With LiDFOB, the collector corrosion may be prohibited as it creates a passivation layer comprising Al-F and borate groups. Furthermore, substituting the one F group with the nano-fluoro butane group may suppress the side reactions at the anode. Lithium (fluorosulfonyl)(nonafluorobutanesulfonyl)imide (LiFNFSI) has high thermal stability, does not corrode Al within ~4.5 V vs. Li/Li^+^, and does not decompose after two weeks of storage at 80 °C. The formed SEI layers are dominated by reductive species of FNFSI- anions. Long Chen’s group recently demonstrated a unique water-in-salt electrolyte strategy (Me_3_EtN-TFSI) based on ammonia salt. They observed an increase in the solubility of LiTFSI with a potential of 3.25 V and 145 Wh kg^−1^ (energy density) above 130 cycles at high retention rates. This increase occurred because the water-in-salt electrolyte exhibited extraordinary ionic conductance (0.90 mS cm^−1^), meager viscosity (407 mPa s), and a broad electrochemical window (1.7–4.9 V vs. Li/Li^+^). Additionally, the inert cation (ammonia salt) in the electrolyte doubled the solubility of LiTFSI, increasing the saline molarity. This extreme concentration of salt generated modifications in the solvation framework of Li^+^, diminishing the H_2_O content and endorsing the accumulation of ions. Though a high salt concentration was used, the electrolyte remained stable (optimum viscosity and conductivity). The water-in-salt electrolyte partially counterbalanced the anodic problems with concentration modality, which forced the anions into the Helmholtz layer, utilizing interphase SEI kinetics to repress dendrite formation [264,265]. Li coated with covalently assembled organic moieties promotes fast ion migration in the electrolyte, generating a Li^+^ transference value of 0.8 and high ion conductance. Furthermore, the rapid transport of Li hinders dendrite formation in a LiCoO_2_ cathode even at high voltages [266]. Similarly, ionic electrolyte 1-ethyl-3-methylimidazolium bis(fluorosulfonyl)amide has been proposed in order to achieve a high operational temperature window in Li batteries along with dendrite-free anodes. Ionic liquids show better dendrite suppression than organic electrolytes as they induce less polarization and prevent dead Li accumulation [267]. Figure 27 clearly depicts the growth morphologies and suppression in organic and ionic electrolytes. For comparative analysis and consistency, recent electrolytes, cathodes, and their characteristic performance in Li-ion batteries are listed in Table 5.

#### 6.1.2. Electrolyte Additives

Additives play a vital role in improving the electrochemical stability and lifecycle of batteries. The additives must have a lower LUMO level than the solvents in order to undergo a reduction process and form a stable passivation layer [292]. There are two types of additives: reduction-type and reaction-type. Reduction-type additives include carbon–carbon bonds, sulfur compounds, nitrite-based, halogenated lactones, and carbonates. Reaction-type additives include aromatic compounds, carboxyl phenols, esters, anhydride, and succinimide. Wan et al. developed a new dithiol-based electrolyte additive, dicyano-1,3-dithiol-2-one (DTO), which increased the cyclic potential and capacity of a Li-ion cell. The discharge capacity was retained up to 75% beyond 210 cycles with 0.1 wt% of DTO. This capacity retention was due to the ability of DTO to hinder carbonate and nickel decomposition in the cell, forming a full-bodied cathode interfacial layer. Furthermore, CEI reduces the polarization and internal resistance of the cell [293]. Mccloskey and co-workers included various additives (<5% volume) in a LiFePO_4_/graphite cell. They found that 15-crown-5 ether exhibited better solvability, diffusion, and increased conductivity [294]. Crown ethers in the battery improve the solubility and transport of Li^+^ ions due to their selective lithium dissociation. Their strong cation-binding ring structures and oxygen interlocking makes them preferable. However, more research on crown ethers applied in various electrolytic media is required. Further, 18-crown-6 ethers with a space group of *S*6 form excellent bonding with metal cations, especially potassium (binding constant 106 M^−1^ in methanol). Future investigations on crown ethers may achieve superior and more credible results. Vinylene carbonate, ethylene sulfite, and fluoroethylene carbonate are also employed as additives in Li-based batteries in order to enhance their capacity and suppress dendrite growth [12,15,295]. 

Another group reported a fluorinated inflammable phosphate electrolyte (thermally stable) that delivered a wide electrochemical window above 4.3 V. The phosphate-based electrolyte, LiPF_6_ with tris(trifluoroethyl) phosphate (TFEP), vinylene carbonate additives, and fluoroethylene carbonate, demonstrated excellent redox stability over a Si-Si-C anode and Li-Ni-MnO cathode [296]. The TFEP electrolyte exhibited finer capacity preservation (72%) beyond 100 cycles and coulombic efficiency of 99.7% with meager dendrites. This high capacity was due to the increased electronegativity and lower polarizability of fluorine in phosphate electrolytes (TFEP). Furthermore, vinylene carbonate enhances the formation of a sturdy CEI–SEI, reducing dendrite formation. Some of the most frequently used additives are listed in Table 6 [297,298,299,300,301,302,303,304,305,306,307,308,309].

Jilin Hu’s group demonstrated Li_3_AlF_6_ derived from the cryolite phase as an electrolyte additive to augment Li dendrite suppression [310]. The Li_3_AlF_6_ additive enhanced the cyclic stability of Li/Li^+^ for more than 100 cycles at 3 mAh cm^−2^. This enhancement occurred because Li_3_AlF_6_ exhibits excellent ionic conductance (∼10^−5^ S cm^−1^) at ambient temperature and better morphological characteristics, simulating Li-ion transport and homogeneous current distribution across the SEI. The authors confirmed that the dendrite suppression occurred due to the integral coating of a thin layer over the nanoparticles. Fluorine-based salt additives in non-aqueous electrolytes form an ample LiF interfacial layer and CEI accompanied by parallel dendrite growth, which is more favorable for a high battery capacity than vertical growth. The LUMO of fluorine salts was lower than that of the electrolyte, indicating that the reduction of fluorine salts occurred at higher potentials than the electrolyte (LiTFSI) [311]. 

One recent research analysis revealed the addition of Li_7_La_3_Zr_2_O_12_ fillers in a polymer electrolyte matrix, which reduced the polymer crystallinity with an increase in ionic conductivity and mechanical strength. It is possible that the improved strength mitigated the further growth of dendrites [312]. Biyi Xu and co-workers employed a Li_3_PO_4_ additive in Li_6.5_La_3_Zr_1.5_Ta_0.5_O_12_ (LLZTO) to augment the ionic conductance of the electrolytic medium. Li_3_PO_4_ developed an interfacial (in situ) interaction with the Li plate, forming Li_3_P during the charging process, stabilizing the interfacial layer, suppressing dendrite progression, and resulting in a high charge capacity [313]. Nano-dimensional additives have been increasingly investigated because of their large surface-to-volume ratio and desired properties at a low scale. Chunhui Gao and co-workers reported the alleviation of dendrites and volume effects by adding aluminum nitride flakes and a 3D specialized carbon current collector [314]. The control and regulation of dendrites were attributed to a strong binding force between the Li anode and Al-N nanoflakes. A Li fluorophosphate (LDFP) additive was reported to support the emergence of a stable SEI after several charge–discharge cycles, reducing the overall internal resistance of the cell. The coulombic efficiency increased by ~10% (from 84–95%) with the appropriate addition of LDFP. The low impedance value and reduced overpotential account for the stabilization of the SEI with LDFP [315]. Thiourea as an electrolyte additive in LiTFSI/TEGDME prolonged the cyclic mechanism at a current density of 2.5 mA cm^−2^ (areal capacity 4.9 mAh cm^−2^), with increased internal resistance and degree of polarization. A cell with 1 M Thiourea was found to last for up to 5345 h (1336 cycles), which is much higher than a cell without Thiourea, which can last for up to 1550 h (388 cycles) [316]. Without the addition of Thiourea at 2.5 mA cm^−2^ (current density), there was higher polarization between the third and fifth cycles. In contrast, the cell operated with the addition of Thiourea was steady over 20 cycles at the same current density. 

Salts of other metals have been employed as electrolyte additives in lithium-based batteries to suppress dendrite growth. Soluble Mg(TFSI)_2_ was added as an electrolyte additive in a Li-Cu-based ether electrolyte system. The Mg atoms exhibited a low-diffusion energy barrier, modulating the lithiophilic sites to soften the morphology at a high current density. The additive salt of Mg created an in situ plating accompanied by the reduction of Li atoms. The authors noted a stable lifecycle over 230 cycles at ~5 mA cm^−2^ current density with mild voltage hysteresis (60 mV) [317].

#### 6.1.3. Design of Stable Electrode–Electrolyte Structures

The cell components’ structural and surface changes facilitate the tuning of electrode–electrolyte interfaces, leading to smooth Li stripping without solid whiskers or dendrites. During the last decade, Min-Sik Park developed a solid-state layered electrode, Li_X_MO_2_ (M = Ni, Co, Fe), and Li_2_MnO_3_ as a high-voltage oxidizable cathode owing to its high capacity. However, the oxidizing property led to safety concerns, which were then regulated using Al_2_O_3_ and AlPO_4_ surface modifiers [318]. Doping of composite and highly uniform structures on the cathode contributes to high cyclic efficiency and dendrite suppression. Fenghua Zheng demonstrated the high electrochemical stability of Li batteries by coating gadolinium-doped ceria onto the cathode surface. The dopant coating repressed oxygen loss during the initial charging process and facilitated stable passivation (CEI), reducing dendrite development [319]. In another study, coating of Li_2_SiO_3_ over a Li_0.2_Mn_0.5_6Ni_0.17_Co_0.07_O_2_ cathode accelerated the surface adhesion and Li^+^ diffusion through the modification of surface structures. Before coating, the XRD results exhibited parallel lattice fringes with 0.47 nm of d-spacing accredited to the (003) plane. After coating a uniform layer of Li_2_SiO_3_ (~5 nm), the new d-spacing (0.33 nm) correlated to the (111) plane, which favored surface smoothness without altering the inherent bulk structures [320]. Nitrogen and fluorine dopant oxide-rich cathodes improve the electrochemical window and alleviate homogeneous SEI formation. A Li_1.2_Mn_0.54_Ni_0.13_Co_0.13_O_2_ electrode modified with fluorine offers a high capacity of ~290 mAh g^−1^ at 0.1 C, charge preservation up to 88.5% at 5 C, and high rate capability of 263 mAh g^−1^ at 0.5 C after 500 cycles [321]. 

Considering the concepts of molecular physics, we recognize that particles orient together towards one direction through a physical force. This coalescence may be scientifically termed adhesion–cohesion interactive forces. Firm adhesion of SEI layers to the anodic surface and CEI to the cathodic surface considerably reduces the possibility of the wearing off of the passivation layer. Several researchers have investigated molecular modulations for the optimization of adhesion towards the passivation layer and electrode interface. Recently, Yanyan Wang introduced silane coupling agents to accelerate Li-O-Si adhesion at the interface. The coupling agents actively modified the host structure to a densely packed uniform surface with a well-built grip (bonding), reducing the corrosive effects and dendrites [322]. Different electrolytes account for the formation of diverse solid electrolyte interphases in Li batteries. Some of the most commonly used electrolytes and the possible contents of their SEI layers are listed in Table 7.

Silane coupling agents have been used to boost the electrochemical performance of a Co_3_O_4_ anode in LIBs [322]. Co-polymerized methyl propylene trimethoxy silane (MPTS) and vinylene carbonate (VC) yield a rigid 3D framework coupling agent, which has been used as a super-ionic surface modifier to control excessive dendrite growth in Li-based batteries. The functional groups present in the MPTS favor the regulation of strong coordination and interaction with Li^+^ ions, preventing unwanted diffusion and side reactions. The polymer further slows down dendrite proliferation through mechanical rigidity and electrode blocking [323]. 

Solid-state electrolyte batteries are a promising alternative to liquid electrolytes, with strong potential to meet the high-energy needs of the future. Solid-state batteries have ease of usage in portable electronics, grid storage, and electric vehicles. However, the rapid SEI degradation and high impedance for Li^+^ diffusion reduce the battery performance. Inorganic compounds in the SEI are frequently reported to have less intimate contact with the electrode, whereas organic moieties with lower contact angles strongly reinforce the electrode, creating a stable interphase layer [324]. Ionic liquids have facilitated enhanced electrochemical performance through increased cell impedance and revised surface structures with a durable SEI.

Solid-state batteries (SSB) are believed to be at the forefront of next-generation automobile batteries. Highly safe and promising batteries can be developed with the proper selection of solid electrolytes. Enhancing the interfacial electrochemistry with reduced internal resistance will improve the battery performance, reducing energy and heat losses. Present-day SSBs encounter difficulties related to ionic conductivity, interface resistance, mechanical rigidity, and the electrochemical window [325]. The most common SSB electrolytes fall into three categories: (i) ceramic oxide and sulfide electrolytes, (ii) polymer electrolytes, and (iii) hybrid electrolytes. Though ceramic electrolytes such as LISICON and LiPON are affordable, with superior mechanical strength, they suffer from fluctuating interfacial resistance and poor electrochemical stability [326]. Weak contact at the interface leads to severe polarization with increased impedance and formation of protrusions, reducing the coulombic efficiency, cycle stability, and conversion efficiency. Li_3_N-type electrolytes possess a small electrochemical window, whereas argyrodite, antiperovskite-type, and sulfide types are not stable at ambient temperatures [326]. In general, inorganic solid electrolytes remain challenging to process due to their mechanical brittleness. Garnet-type and NASICON-type LISICON electrolytes have relatively stabler performance characteristics than other ceramic electrolytes [327,328]. More extensive research efforts in this area may lead to the development of a superior ceramic-based SSB.

Since the dendrite deposition is relatively higher in liquid electrolytes due to liquid–solid interactions, switching to solid-state devices has been suggested as a viable option. Solid polymer electrolytes (SPE) are resilient, with high plasticity, safety, and wettability and minimal interface resistance. Before using SPE, it is necessary to understand the polymer solvation chemistry with metal ions and the decoupling mechanism. Increasing the decoupling resistance renders the dissociation of lithium and polymer chains more difficult when the working temperature is lower than the glass transition temperature of the polymer used [327]. The solvation of Li^+^ ions in the polymer matrix improves when lowering the lattice constant and increasing the dielectric constant of the metal salt compared to the polymer [329]. Reducing interfacial defects with suitable metal salt and polymer matrix selection has been proposed to enhance SPE activity. Hybrid polymer–ceramic solid electrolytes suffer from variable ionic conductivity and material compatibility. Hybrid SSE is an important research domain, as several parameters, such as combined dispersion conformity, device compatibility, filler size in the polymer matrix, the electrolyte’s thickness, and the electrolyte’s weight, play a significant role in the SSB operation. As the particle size becomes small, the interaction region between the ceramic filler and matrix increases, reducing ion migration. The concentration of filler and polymer matrix, morphology, and dispersibility also influence ion movement [330,331].

Nevertheless, in recent years, dendrite growth has been observed in SPE. Engineering of the polymer matrix, enhancement of the mechanical stability, and the utilization of nano-additives may resolve the formation of dendrites. Tian et al. congregated and adapted an elastic MOF sheath prepared by the vacuum-assisted method. The self-sustaining MOF sheath was relatively sturdy, able to block dendrite perforation [332]. Tang et al. incorporated 2D clay additives into a PEO solid electrolyte and observed the suppression of dendrites, with improved ionic conductance and thermal resistance. The additive materials acted as a filler with a high Young’s modulus of 175 Gpa, controlling dendrite growth through mechanical resistance [333]. Chen et al. reported the use of a lithiophilic fluorinated yttrium anode in a quasi-solid electrolyte, which greatly inhibited dendrite growth through the formation of a LiF-rich, stable SEI layer [334].

The surface roughness of Li metal induces severe volume expansion and disorder deposition of electrolyte species, generating uneven dendrite growth. However, only a few articles are available concerning the removal of uneven surface glitches of native anodic layers. Elimination of the jagged surface troughs by scraping with sharp objects is a commonly used method. However, the sharp edges may generate damage and permanent breaks on the surface. Another solution is to wash, clean, and polish the material using chemicals. However, chemicals are unreliable as they can lead to unwanted reactions, changing the electrode components. Recently, a cost-effective, straightforward approach was developed with significantly lower material wastage using a mechanical system. Uniaxial stress (pressure) was applied on thin electrodes employing a benchtop two roll-press, which was then moved gradually, reducing the distance between the rolls. This mechanism was able to remove a uniform thin layer from the surface, resulting in smoothness and a defect-free electrode surface. The uniform surface favored the homogeneous deposition of ions during lithiation, improving the efficiency and mitigating dendrites [335]. 

#### 6.1.4. Interfacial Formulations with Artificial Films

Spontaneously generated natural SEI layers are fragile in most cases. Either additional components or electrolyte modifications must be used to accommodate Li stripping and suppress dendrite growth. However, this additional process requires extra effort and often results in only mild improvements. Instead, artificial SEI layers prefabricated into the anode surface offer better kinetics and predicted behavior while requiring less effort. An artificial SEI aims to offer enhanced mechanical and thermal stability, preventing the unwanted deposition of protrusions. A commonly used technique for preconditioning the anodic surfaces is the use of surface coatings. Various oxides such as Al_2_O_3_, SiO_2_, ZrO_2_, TiO_2_, and ZnO have been employed as layer coatings on the anode. Moreover, 2D and 3D reinforced anodes have been adopted recently, improving battery performance by adapting homogeneous SEI layers. A 2D structured covalent organic framework comprising trimethoxy benzaldehyde and terephthaldehyde derivatives was uniformly deposited as an artificial SEI thin film (~10 nm) over a Li anode. This thin coating led to the redistribution of Li-ion flux, resulting inhomogeneous stripping behavior during the cyclic process.

Furthermore, the mechanical properties of the deposited film sustain the stress due to its high elastic modulus (6.8 Gpa) and resistance to dendrite growth, diminishing the likelihood of short-circuiting. An outcome of 400 h stable cyclic performance at 1 mA cm^−2^ was recorded in Li–sulfur batteries [336]. Moreover, 3D scaffolds such as a Li_2_S layer were coated onto a Cu current collector surface to accelerate uniform metal stripping and reduce dendrite growth. The Li_2_S protective layer passivated the Cu collector and counterbalanced the ion–electron transport rate, resulting in agglomerated structures in the worn-out spaces of the SEI. As a result, stable cycling (500 cycles) at 1 mA cm^−2^ was observed [337]. Kuriong Deng and co-workers designed a 3D cross-linked single ion transportable SEI layer over the anode surface. The 3D polymer was formed through the reaction of pentaerythritol tetrathioglycolate and lithium bis(allylmalonato)borate. The covalently bonded bis(allylmalonato)borate exhibited movement confinement, allowing only single-ion transport (Li^+^) through the polymer matrix. This single-ion transport promoted the uniform deposition of ions and improved ionic conductance due to the weak electrostatic interactions among the sp^3^ hybridized boron and Li ions [338]. 

The Li metal, organic moieties, solvents, and salts react internally, forming protective SEI layers. The selected chemicals respond before the cell loading and form a uniform SEI in an ex situ approach. Various organic frameworks have been utilized through both in situ and ex situ techniques. Zn-PVA-based metal–organic frameworks (MOF) have been employed as artificial SEI with the “cement-glue concept”. In other words, PVA acts as a glue, attaching the Zn-MOF to the Li surface. The combination of Zn-MOF generates uniform Li-ion flux, which inhibits dendrite growth and suppresses the volume effects. The mechanical strength and wettability of Zn-MOF and highly viscous PVA (non-toxic and eco-friendly) improved the coulombic efficiency ~97.7% after 250 cycles at 3 mA cm^−2^ with the capacity of 135 mAh g^−1^ after 100 cycles at 1 C [339]. Li-Al-Ge-based phosphate (LAGP) with polyethylene oxide (PEO) as glue was deposited on metallic Li. The LAGP improved the interphase compatibility, owing to longer charge retention and reduced polarization [340]. Amorphous carbon-based organic hollow structures with high conductance (~7.5 S m^−1^) were employed as a layered coating over a Li anode. The layer substantially prevented electrolyte leakage and dendrite progression and resulted in high coulombic efficiency (~99.5%). Graphene, hexagonal boron nitride, and sulfur additives were also utilized for the formation of uniform SEI. Si-based nanoparticles were employed as prelithiation agents to form SEI layers comprising LiF and lithium alkyl carbonate similar to native SEI. Here, the Si-Li nanoparticles were reduced using 1-fluorodecane to create a dense and continuous coating on the anode, resulting in a high cyclic capacity (1600 mAh g^−1^). Similarly, tin, silicon, and graphite were developed as a coating (artificial SEI), which improved the cell performance by suppressing dendrites [341]. For instance, reduced graphene oxide (r-GO) and nanofibers were coated on metallic lithium. The rGO increased the surface area and porosity and minimized the volume effects and interphase fluctuations. Thus, a highly stable SEI with sufficient mechanical strength, wettability, and adhesiveness led to improves cell capacity, reducing dendrites [342]. 

The coating of metal-based phosphorus layers has significantly improved cell performance. Lithium phosphorus oxynitride (LPON) (~100–300 nm) was coated over Si anodes using magnetron sputtering. Various ex situ analyses showed that the LPON coating dramatically reduced the volume expansion and parasitic electrolyte decomposition, improving the cyclic stability due to increased ionic conductivity, namely 9 × 10^−7^ S cm^−1^ at ~18 °C. Furthermore, the electrode structure was integrated because the artificial coating led to a continuous SEI layer during the cyclic process [343]. Another research group formulated titanium oxide and lithium n-butoxide hybrid layers as artificial SEI. An asymmetric battery with the fabricated SEI layer demonstrated a capacity of 140 mAh g^−1^ at 0.5 C, accommodating ~100% charge retention beyond 600 h. The charge transfer resistance (134 ῼ) and Li-ion transfer number (0.42) of Li cells with artificial SEI were sufficiently greater than a bare Li cell’s transfer resistance (295 ῼ) and Li-ion transference number (0.36). Moreover, the hysteresis of the cell fabricated with the artificial SEI decreased gradually, enhancing the cyclic stability [344]. 

**Figure 28:**
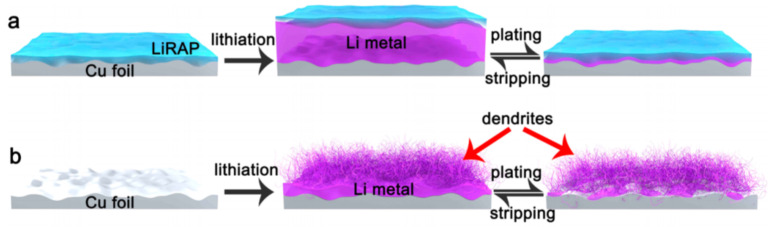
(**a**) Dendrite growth suppression in LiRAP/Cu substrate (**b**) Growth of dendrites without LiRAP in Cu-Li substrate (adapted with permission from [350] Nano Lett, ACS, 2020).

Polymers endure the lightweight and elastic nature of the formed SEI owing to their high energy densities. In recent years, polymers have been widely used to create stable SEI. Yicheng Zhong’s group developed an alginate-based Li artificial SEI, which was chemically stable and permitted faster Li-ion transport. Li cells with this artificial SEI in a LiPF_6_–ethylene carbonate mixture and fluoroethylene additive delivered a high cycle life of 850 h (~99.6% coulombic efficiency). It was observed that the native SEI underwent cyclic rupture and became worn out. However, the artificial SEI self-adapted and continued as a stable layer during the cyclic process [345]. A cross-linked [LiNBH] polymeric layer coated on the Li surface resulted in the uniform distribution of Li-ion flux by forming Li-N bonds with high ionic conductance and negligible electron conductance (insulator). The ionic conductance accommodated faster Li^+^ diffusion due to a regular cycle of up to 800 h at 3 mA cm^−2^ [346]. Inherent polymerization of ethyl cyanoarylate with LiNO_3_ additive was used to form a stable artificial SEI design. The NO_3_- and CN-group in the polymer additive reacted with the anode, forming a durable nitrogenous layer, which accelerated ion transport, hindering unfavorable side reactions. The charge retention of 92% after 500 cycles at 2 C was appreciable. This high-capacity retention occurred due to the dramatic improvement in SEI atoms’ mechanical properties and uniform distribution, inhibiting dendrite growth [347]. In another study, carboxylic benzene diazonium salt was applied on a graphitic anode using various drafting methods (in situ, electrochemical, spontaneous). The entire artificially grafted anode promoted high loading with regulated, uniform SEI, fast Li intercalation, and prevention of graphite exfoliation and dendrite growth [348]. A Nafion membrane and Li-Si-based sulfur membrane forming a double-layer coating were employed in Li–sulfur batteries. The flexibility of the Nafion membrane helped to maintain structural integrity and hindered side reactions. The Li-Si layer improved the diffusion of Li^+^ ions, inhibiting dendrites. The fabricated cell could withstand up to 1400 h at 1 mA cm^−2^, eliminating dendrite evolution and exhibiting good capacity (~783 mAh g^−1^) [349]. Later, anti-perovskites became a popular class of anode materials due to their high conductivity and dendrite-suppressing mechanism. Han et al. reported that the huge difference between the Li-rich antiperovskite and artificial SEI layer promoted the suppression of dendrites in a Cu-Li substrate, also enabling optimal performance for more than 100 cycles. Figure 28 shows the Li-rich antiperovskite (LiRAP) self-regulating the deposited dendrites through its upright coverage [350].

#### 6.1.5. Other Novel Methodologies

The current density distribution in a Li cell tends to be non-uniform because of a local difference in structures, inducing needle protrusions. The in situ approach, as discussed earlier, facilitates the highly ordered deposition of a protective layer over the electrode surface. In this regard, a polyisoindigo derivative (Piso) prepared by Yamamoto cross-coupling was able to efficiently reduce Li-ion flux, generating highly stable Si-Piso structures with a capacity of 1400 mAh g^−1^ over 5000 cycles [351]. Unique Li-spread fibrous Li_7_B_6_ matrix layers are promising in situ anodes for suppressing dendrite growth. Additionally, single-ion transfer polymers and solid electrolytes are valuable approaches in reducing dendrite growth. Atomic layer depositions and the anchoring of metal oxides generate novel structures exhibiting extraordinary electrochemical performance. A Li_4_Ti_5_O_12_ seed layer was grown on a graphite anode through atomic deposition, forming peculiar structures that improved the cell performance [352]. Multiple salt electrolytes and molten salts led to improved thermal stability by reducing dendrite growth and effectively crossing the electrode structures.

Separator modification is another important means of improving cell performance. In particular, wettability accounts for the uniform distribution of Li^+^ ionic flux. This ionic flux is responsible for uniform SEI deposition and dendrite inhibition. Hao Zheng and co-workers used polyether compounds as surfactants to reduce the surface tension and improve the liquid–solid interphase wettability (separator wettability). The contact angle measurements showed a high angle (80.5°) without polyethers, which indicates poor water absorption behavior.

In contrast, the addition of polyethers enhanced the water absorption property, reducing the contact angle to 64.3° [353]. In Li–sulfur batteries, in order to effectively trap polysulfides and prevent dendrites, a 2D scalable step-by-step grown self-assembled MoS_2_-polyacrylic acid double-sided layer on the separator was adopted. The self-assembled layer acts as a physical barrier, preventing the thermal decomposition of the separator, and passivates from the growing dendrite structure. Moreover, the layer prohibits polysulfide shuttle and recombination, improving battery efficiency [354]. Composite separators coated with polyvinylidene fluoride/Li_6.4_La_3_Zr_1.4_Ta_0.6_O_12_ (LLZTO) on one side of polypropylene offer a 3D channel for Li distribution along the PVDF-LLZTO interfaces, resulting in fast Li^+^ diffusion and uniform anodic deposits. The synergetic effect of anode control and Li distribution enhances the coulombic efficiency [355]. Nanoscale coatings on conventional polypropylene separators help in the formation of a protective SEI layer. The systematic coating of Si nanoparticles and polyacrylic acid (PAA) over the separator surface yields a stable, highly conductive passivation layer comprising Li-Si alloy and LiPAA [356]. Various functional modifications and catalyst additions to the separator reinforce the stability of the separator–electrolyte species interface, contributing to higher efficiency and reduced dendrite evolution [357,358]. The self-healing approach intrinsically modulates the protective layers to repair itself, owing to the material’s significant endurance. Zhang and his group demonstrated an electrostatic shield mechanism at the dendrite tip as a self-healing approach employing Cs^+^ additives in the organic electrolyte, eliminating anisotropic dendrites. Furthermore, symmetrically grown compact Li dendrites were observed after using Cs^+^ and Na^+^ additives. Ximing Cu et al. reported a polydimethylsiloxane (PDMS) network linked through imide bonding as a self-healing integrated protective layer that regenerated itself to accommodate volume effects and destroy protrusions. This strategy, with firm adherence to the SEI, results in 99% capacity retention with coulombic efficiency (~99.7%) after 300 cycles [359]. Recently, biomimetic strategic designs have opened up a new route for battery development, offering solutions to the current challenges. In this regard, Nan Chen et al. employed a biomimetic gel electrolyte confined in a 3D SiO_2_ scaffold resembling the nest configuration of ants, which boosted the ionic conductivity and controlled dendrite succession through the spontaneous creation of a dense passivation layer. The as-prepared cell offered cyclic stability up to 3000 cycles with 99.8% coulombic efficiency [360]. 

Novel rotatable, flexible, and foldable electrodes have captured researchers’ attention. Flexible and foldable electrodes possess the merits of durability, trouble-free tilt adjustments, special dimensional coatings, reduced occupancy, effortless surface modifications, and compatibility. Researchers demonstrated a flexible and rotatable Sb_2_S_3_/TiO_2_/C fibrous anode for Li-ion batteries [361]. In addition to this, we demonstrated the use of 1D electrospun TiO_2_ nanofibers with superior characteristics for energy storage applications [362,363,364]. Strong NiFe_2_O_4_ nanofibers, prepared by electrospinning, were used as a Li-ion battery anode with excellent capacity of 1000 mAh g^−1^ with ~100% coulombic efficiency [365].

Birnessite-type sodium molybdate prepared by the simple addition of water molecules has been used as both the electrode (anode and cathode) material as a dual-ion material. The structural arrangement of atoms in this material creates adequate space for volume occupancy, restraining pulverization and increasing ion diffusion [366].

### 6.2. Dendrite Control in Na-Based Batteries

#### 6.2.1. Electrolyte Design and Optimization

Though Na and Li-ions are alike in structure, the electrolytes and their chemistry differ significantly. Some of the salts used in Na-ion batteries are sodium hexafluorophosphate (NaPF_6_), sodium perchlorate (NaClO_4_), sodium trifluoromethanesulfonimide (NaTFSI), and NaFTFSI. Compared to Li, Na has less destructive oxidation–reduction potential (~0.28 V), less Lewis acidity, high solubility, low desolvation energy (<30%), large ionic radius (>20%), high reactivity, and low equivalent volume [367]. Therefore, a new paradigm of Na battery electrolytes is need to provide a bridge between the anodic and cathodic reactions and battery chemistry in order to ensure viability. The first electrolyte for Na batteries was reported in the 1980s as NaI-PC, NaPF_6_-PC, and Na-TiS_2_. 

Lin Zhou and co-workers tuned the electrolyte composition in order to regulate the alloying anodic performance (Sn, Bi). They designed a battery with a capacity of 650 mAh g^−1^ after 200 cycles and coulombic efficiency of ~99% using electrolyte engineering. Utilizing micro-sized Sn at a current density of 500 mA g^−1^ in 1 M NaPF_6_–dimethoxyethane (DME), the electrolyte delivered a capacity of 700 mAh g^−1^. They observed pulverization, which could be controlled through anode–electrolyte optimization. Another study using NaClO_4_, fluoroethylene carbonate and an ethyl methanesulfonate revealed the temperature resistance and improved electrochemical performance of a [Ni_0.25_Fe_0.5_Mn_0.25_]O_2_/C–Fe_3_O_4_ cell with controlled dendrites [368].

Kaikai Li et al. examined a Na-ion battery with a TiO_2_ anatase anode in a diglyme-based electrolyte (NaCF_3_SO_3_ in diglyme). The fabricated cell manifested a reversible capacity of 257.9 mAh g^−1^ at 100 mA g^−1^. The perceived voltage profiles were linear and entirely different from those of Li-ion batteries, indicating that other mechanisms (single-phase Na intercalation) were involved in the TiO_2_ sodiation process. Furthermore, the operating potentials were ~0.8 V lower than Li intercalation (~1.7 vs. Li/Li^+^). The enhanced electrochemical capacity could be attributed to the uniform SEI layer, examined using XPS [369]. The presence of C, O, Na, and F was confirmed using XPS. The quantitative analysis showed that the SEI contained organic groups, which were distributed over the exterior surface, and non-organic groups were present at the interior surface of the SEI. This distinction was made based on the notion that the atomic fraction of C lessens with increasing etching depth (~28% at surface and ~5% at depths). On the contrary, the atomic fraction of Na and O increased with the etching depth.

Besides varying the electrolyte compositions and materials, it is necessary to study the electrolyte interaction and variations with different anode materials. Ponrouch et al. investigated the electrolyte–electrode feasibility with multiple salts and solvent mixtures. The investigation results showed that NaPF_6_, NaClO_4_, and NaTFSI have similar conductivities (~6.3 to 7.3 S cm^−1^) [370]. These similar conductivities highlight that the anion has a trivial influence on conductivity. In comparison, significant variations in conductivity were observed during the change of the solvents. For example, in 1 M of NaClO_4_, the conductivity level of EC: DME was more significant than EC: DMC and EC: triglyme. Moreover, the choice of anion did not exhibit an influence on the electrochemical window. However, the solvents had a significant impact on the electrochemical window. Overall, PC, DEC, EC, and DMC mixtures showed more expansive electrochemical windows and stability. We can infer from this that proper solvent selection can promote ionic conductivity, increase salt dissociation, reduce viscosity, and improve electrochemical stability. However, based on the individual electrodes, the thermodynamic and catalytic activity may vary, contributing to electrolytic decomposition and side reactions [371]. The same research group analyzed the effects of dimethyl ether and dichloromethane as a co-solvent in NaPF_6_-EC. This co-solvent reduced the solution viscosity, developed a stable SEI, and enhanced the ionic conductivity. Later, DME was used with a Na_3_V_2_(PO_4_)_2_F_3_ cathode, revealing secondary polarization losses, capacity retention (98%), good coulombic efficiency (>98.5%), and adequate electrochemical stability. Interestingly, increasing the concentration of electrolyte contents generating a highly saturated electrolyte increases the Na^+^ ion flux, thus increasing the cycle life and reducing dendrite deposition over a Na anode. Here, Wang et al. revealed the use of a highly concentrated electrolyte, 0.5 M of sodium trifluomethanesulfonate in ether, which increased the oxidation resistance of Na against O_2_, promoting homogeneous Na^+^ flux with a reduction in dendrite growth in a Na-O_2_ battery [372].

Solid electrolytes in Na batteries lead to superior thermal stability, conductivity, and enhanced electrochemical properties. Furthermore, a solid electrolyte may provide high flexibility with simpler production, reducing the costs. Inorganic solid electrolytes consist of symmetrical structures as moveable ions, which dislocate from one site to another, creating vacancies (Frenkel and Schottky defects). Crosslinking of compounds and ion migration plays an imperative role in inorganic electrolytic conduction [373,374]. Therefore, the available hopping sites or vacancies have to be optimized for the efficient functioning of the battery. Accordingly, Na_3_PS_4_ structures with 3D pathways along Na_1_ and Na_2_ sites have high conductivity (~10^−4^ S cm^−1^) and are used as solid electrolytes in Na batteries. Low activation energy for hopping (~25 KJ mol^−1^) was observed because of the intimate connection and densely packed grains, attributed to the elimination of dendrites and stable interfacial layer deposits [373]. Integration of Na_4_SiS_4_ into Na_3_PS_4_ increases the ionic conductivity to ~7.4 × 10^−4^ S cm^−1^. Tetragonal-structured Na_3_SbS_4_ also has high room-temperature superionic conduction. Oxide-based β-alumina electrolytes are still commercially employed in batteries, notably in Na-Cl and Na-S batteries. However, the poor chemical stability and unsatisfactory ionic conduction restrict their widespread application.

**Figure 29:**
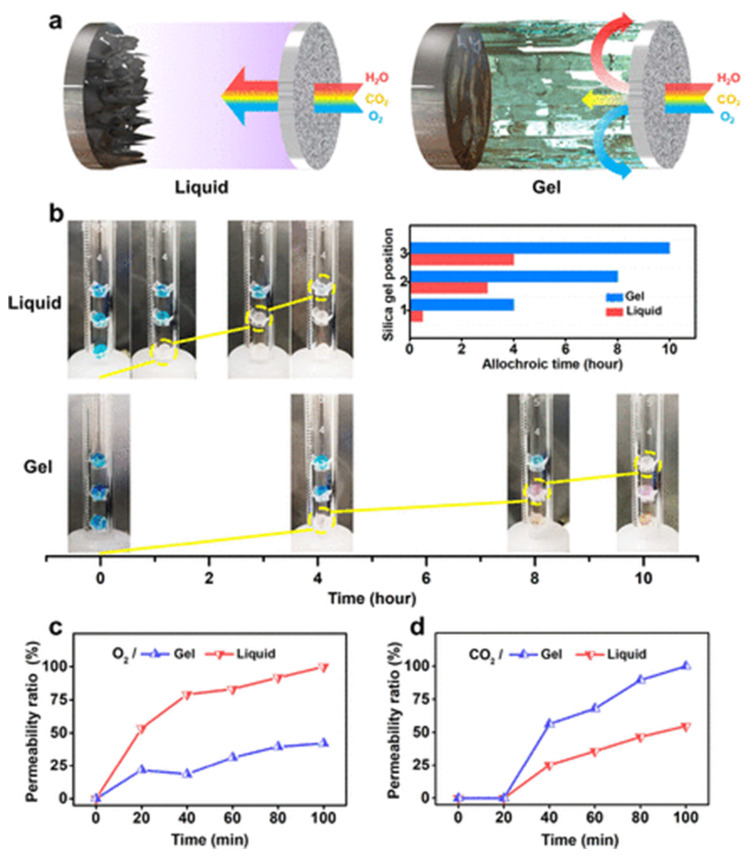
(**a**) Schematic representation of H_2_O and air permeability in gel and liquid electrolyte. (**b**) Snapshots of the anti-water vapor permeability test. (**c**,**d**) GC analysis of corresponding contents (adapted with permission from [374] ACS Cent. Sci., 2021).

Recently, a Li@Na anode was prepared by cross-linking lithium ethylenediamine at the anodic surface and tetraethylene glycol dimethyl ether from the electrolyte, forming a stable gel electrolyte [374]. This new type of gel electrolyte effectively inhibited Na dendrites by the electrostatic shield effect mechanism, controlling the migration of surface charges. Additionally, the gel electrolyte prevented the cross-reaction of water and air, thereby delaying the anodic corrosion reactions, as shown in the Figure 29. From the analytical characterizations, the amount of O_2_ crossover was 40%, with no CO_2_ crossover observation for the initial 20 min; later, 55% CO_2_ crossover was noted due to the absorption of CO_2_ by ethylenediamine. Notably, no H_2_O and O_2_ crossover was observed, which accounted for the stability of the Na anode [374].

#### 6.2.2. Binders and Additives for Electrolyte

Similar to Li batteries, the inclusion of additives in electrolytes leads to variations in the electrochemical performance and SEI formation in Na batteries. Several species, such as fluoroethylene carbonate (FEC), vinylene carbonate, and ethyl sulfonate, were used to increase the performance of carbon: NaNi-Mn-O_2_. Nevertheless, the presence of FEC influenced the cyclic performance of both electrodes. FEC increased the electrodeposition rate, accompanied by stable SEI formation. Another research group reported an excellent cycle life (1250 cycles) and rate capability of 10 C while using a 1 M NaClO_4_ TEGDME ether-based electrolyte with a P_2_-NaCoO_2_ layered cathode and a graphite anode [375]. The graphite anode material was found to be suitable, yet the intercalated solvent molecules in the layered structures were depleted, reducing the energy density of the system.

Ether-based electrolytes are fascinating because of their stable cyclic structures. A diglyme electrolyte with NaPF_6_ exhibited an excellent electrochemical stability window with limited oxidative decomposition in the voltage range 0–4.4 V vs. Na/Na^+^. This excellent electrochemical stability was due to the nonexistence of side reactions and stable SEI layers, preventing dendrite extension. For further support, DFT calculations revealed the matching redox potentials for several complexes of diglyme (Na^+^ and PF_6_^−^). An increase in glyme molecular weight enhanced the intercalation potential. However, there was a decrease in rate capability. Yuqi Li et al. revealed that sodium batteries could work under ultra-low concentrations (0.3 M) of electrolytes. While using low concentrations of electrolytes, the researchers could achieve the following advantages: (i) cost reduction, (ii) broader working temperature range (−30 to 55 °C), low viscosity, minor corrosion, high coulombic efficiency, and stable SEI/CEI interphases, prohibiting dendrite growth [376]. 

The SEI layers on carbon-based anodes in Na-ion cells imbibe partial Na from the cathode, limiting the energy density and cyclic stability. Yu-Jie Guo and co-workers demonstrated this by modifying a cathode with an additive slurry deposition comprising Na_2_O_2_ (sodium peroxide). The modified cathode showed high storage capacity, better charge retention, and high rate capability due to the formation of a stable SEI layer on the anode [377]. NaPF_6_ salt with the addition of fluoroethylene carbonate displayed excellent electrochemical performance due to the creation of sturdy passivation films composed of sodium ethylene dicarbonate and NaF compared to DMFC additive in NaTFSI [377]. Polymer additives are also employed in Na-ion batteries. In a NiS-based anode, a polymer additive (amino-ended hyperbranched polyamide (HP–NH_2_).HP-NH_2_) was used to improve the cyclability, preventing particle aggregation.

Furthermore, the needle structures of the NiS additive successfully initiate Na^+^ diffusion. The fabricated battery delivers 590 mAh g^−1^ over 1000 cycles [378,379]. Ethylenediamine additives have recently gained attention, especially in ether-based electrolytes. The specific amount of ethylenediamine addition forms a Na_2_S_2_O_4_ self-healing SEI layer with high discharge capacity, reduced polarization, and negligible corrosion effects [380]. As discussed earlier, Rb^+^ and Cs^+^ have also been studied as good additives for carbon-based electrodes in addition to FEC. Succinic anhydride was observed to perform better in hard carbon-based anodes than FEC at 60 °C. The current densities decrease with succinic anhydride, denoting the lack of charge transport at the electrode–electrolyte interphase (EEI) or the generation of a high-resistance SEI layer, preventing dendrite formation [381]. As with Li-ion batteries, lithium difluoro(oxalate)borate salts (LiDFOB) are used as additives in Na-ion batteries to improve the electrochemical stability of the SEI [382]. Carbon black serves as a good additive in Li and Na batteries because it is easy to prepare and abundant. The reversible capacities of batteries utilizing carbon black additive were reported to drastically increase from 213 to 564 mAh g^−1^ for Li and 92 to 209 mAh g^−1^ for Na, respectively. Salt-based diethylenetriamine penta acetic acid (DETAP) acts as a sacrificial Na delivery tool for unifying the sodiumless Na-MnO_2_ cathode. The DETAP salt consists of five Na ions, which increases the reversible capacity and offers presodiation modifications in the cathode by reducing oxidative and dendrite effects. By adding DETAP, the charge capacity could be increased from ~60 to ~130 mAh g^−1^ [383]. 

#### 6.2.3. Modifying Other Components

Separators perform a pivotal function in dendrite suppression, depending on their porosity and wettability. An Al_2_O_3_-deposited polyethylene oxide (PEO) membrane with thiol cross-linker prevents dendrite growth in redox flow batteries. In high-energy-density storage devices (batteries), the separator must hinder the cross-over of redox molecules from catholyte to anolyte or vice versa. Polymer separators with modified mechanical, thermal, and electrochemical properties through cross-linking or coating are extensively used in Na batteries to hinder dendrite spread out to the cathode. PEO prepared by step polymerization of thiol group precursors with a suitable initiator, catalyst, and cross-linking agent generates a resilient, firm membrane [384]. A defect- and bead-free polyvinylidene fluoride (PVDF) separator synthesized by facile electrospinning technology exhibited excellent flexibility and dendrite blockage, with an overall Na^+^ transfer rate of 98.3% and ionic conductivity of 7.38 × 10^−4^ S cm^−1^. Upon the implementation of electrospun PVDF in a Na electrochemical cell with a Na_0.66_Fe_0.5_Mn_0.5_O_2_ cathode, coulombic efficiency of 92% was attained [385]. Nylon-11 polymer fibers implemented using novel, tailored co-solvent polymerization with the complete elimination of a phase with a pseudo-hexagonal structure led to good piezoelectric effects, ionic conductivity, and inhibited electron cross-over due to its good suppleness and wettability [386]. Upright growth of silica aerogel over a polyacrylonitrile (PAN) membrane separator results in finer thermal stability (~280 °C) at low contact angles, enabling optimum cell performance in Li and Na metal anodes [387]. Water-soluble cellulose separators modified with carboxyl methyl and hydroxy groups are used in solid-state batteries. However, thermal instability and safety concerns during thermal runway limit their application [388]. Glucose biopolymers such as dextran acted as a cross-point, bonding a reduced graphene oxide (r-GO) nano sheath and 2D layered transition metal oxide cathode. This surface modification of the cathode with r-GO casing shielded the interior surface from degradation and promoted high ionic diffusion due to the improved surface area [389]. However, biopolymers experience temperature constraints. Thermally stable metal–organic frameworks or the infusion of high-temperature ceramic materials such as Nafion into bio-derived separators (cellulose) may improve their usability. Commercial Nafion (C_7_HF_13_O_5_SC_2_F_4_) mixed with EC and PC reveals high ionic conductivity at room temperature—3.52 × 10^−4^ S cm^−1^ and 1.52 × 10^−3^ S cm^−1^ at 70 °C—and is therefore employed as a separator and as an electrolyte in Na-ion batteries [390]. Ceramic state Na_3_Zr_2_Si_2_PO_12_ (NASICON membrane), which possesses high Na^+^ ion conductivity (2 × 10^−3^ S cm^−1^), was utilized as a separator in a Na-O_2_ battery. With proper optimization of electrolyte concentration, membrane thickness, airflow, catalyst, and cathode material, the performance characteristics of NASICON can be strengthened [391]. Dual polymers (PVDF-PP) sandwiched between layers of titanium oxide nanoparticles as separators contribute to better electrochemical operations. It is widely known that TiO_2_ has the capacity for Na^+^ capture, fostering a stable NaTiO_2_ layer at the anodic surface under appropriate conditions. Adopting the same strategy, tailoring TiO_2_ as a membrane separator will effectively react and block the Na dendrites [392]. 

Surface modification of electrodes may augment the adhesion and regularities, leading to sites for solid SEI disposition. The O_3_ class of layered oxides is increasingly utilized in alkali metal batteries. However, their poor rate capability, dangerous cycling, and inconsistent potential due to structural mismatch and poor diffusion prevent their practical usage. Zn and its derivates have shown promising improvements while supplanted into O_3_^−^ type oxides. Higher concentrations of Mn and Fe are unfavorable in certain circumstances (depending on the electrodes and electrolytes). Substitution of Zn^2+^ to Mn^3+^ or Fe^3+^ may lead to structural stability, building a stable oxide passivation layer. Significantly, Zn ions elevate oxide-phosphate reversible phase transformation, facilitating easier Na^+^ insertion and exertion [393].

Similarly, a multilayer coating on a Na anode with metal oxide (SnO_2_) as an intermediate and carbon cloth as the top layer (buffer layer) improves the storage capacity (~60%). SnO_2_ is widely used as an anode material in Na-ion batteries because of its high theoretical capacity (1378 mAh g^−1^) and electrochemical feasibility. Nevertheless, bare Na-SnO_2_ suffers from uncontrollable volume occupancy and deprived kinetics. Spongy carbon cloth can accommodate the volume discrepancy in proximity with sodiation, yielding better cyclic operation and capacity. In the same way, Al_2_O_3_–carbon cloth on a Na anode may lead to a significant capacity amplification [394]. 

Layered oxides with multi-metals (Na_2_Ti_3_O_7_) are considered supreme electrode materials for high-storage NIBs because of their good potential ranges (~0.3 V vs. Na/Na^+^), excellent cyclability, and low cost. Nevertheless, poor conductivity and substandard Na^+^ distribution confine the usage of Na_2_Ti_3_O_7_. Alteration of the Na_2_Ti_3_O_7_ surface with N-doped graphene quantum structures (0D) narrows down the diffusion path, enabling faster ion kinetics. Moreover, the large surface area and ultra-smooth surface favor the dense packing of interphase deposits, leading to firmly anchored SEI formation over the anodic facet. Integration of quantum structures, 0D nanodots, 1D nanowires, 2D nanosheets, and 3D microclusters into the anodic surface improves the Na^+^ reaction kinetics [395]. Core–shell and encapsulated structures offer relatively high diffusion kinetics due to their structural consistency. However, simulation results show the development of tensile radial stress at core–shell interfaces during the intercalation process, which may annihilate the structure if the thickness of the outer layer is thin, accommodating a heavier core. Thus, a thicker shell with a lightweight, compact core exhibits excellent electrochemical performance due to its harmless structural reinforcement and interfaces [396]. 

#### 6.2.4. Interfacial Layer Alteration

Highly durable SEI layers can facilitate the execution of high-energy-density batteries. However, interfacial layers at both anodic and cathodic ends suffer from cracks and substandard chemical compatibility. In-situ-generated MoS_2_-Na hybrid artificial SEI layers redistribute the products (Na_2_S) as homogeneous deposits on the anodic surface, and the leftover MoS_2_ nanostructures act as a 3D host to confine anode, accommodating volume variations. Varying the cathodic interphase layers supports the fastest ion transport, neglecting dendrite formation. In this regard, lithium difluoro(oxalate)borate salt was integrated between a Ni-based cathode and EC/DEC/DMC-polydioxolane (PDXL)-LiPF_6_ (solid/liquid hybrid electrolyte) to form a highly amorphous and stable artificial cathodic interfacial layer [397]. NaF and Na_2_CO_3_ were pre-induced into the electrolyte to include homogeneous interfaces with the Na_0.67_Fe_0.5_Mn_0.5_O_2_ cathode. The formed CEI comprising NaF and Na_2_CO_3_ prevented side reactions, forming artificial thick cathodic interphases and reducing volume expansion [398]. 

Fe_7_S_8_ core–shell nanostructures (~10 nm) are the most recently developed anode material in Na–sulfur batteries. These quantum structures can resolve the sustained thermodynamic and kinetic challenges in conversion reactions. An additional carbon buffer (artificial SEI) layer over the quantum electrode structures prevents drastic volume expansion and controls dendrite intensification, resulting in excellent capacity (550 mAh g^−1^) and 71% charge retention after 1000 cycles [399]. 

Metals such as tin, antimony, and selenium have been embedded as nucleation layers in Na-based batteries [400]. Nitrogen-doped antimony with a highly porous polymer or carbon composite is used in Na batteries. The porous structure enables the trouble-free diffusion of ions and offers additional void sites to compensate for the significant volume expansion. Further embedded core–shell structures or spherically wrapped anodic alloys with a carbon-based buffer layer protect the inner or core particles from corrosion and dendrite accumulation. Tianjing Wu’s group reported using an Sb-C nanocomposite alloy anode with a high storage capacity of 500 mAh g^−1^ due to the structural integrity of the negative electrode and the electrical resistance of the formed SEI [401]. A Na alloy with embedded Sn nanoparticles can cross the nucleation blockade as a result of in-situ-developed sodiophilic structures. In addition, carbon structures can be reinforced into the Na-Sn alloy matrix for improved stiffness and uniform nucleation sites. Moreover, 2D and 1D designs with electron confinement and tunneling effects contribute to the streamlined ejection of ions, forming regular deposits on the electrode surfaces [402]. Tin and carbon nanostructures for optimizing nucleation sites and buffer protection, respectively, were successfully employed in Na batteries with enhanced outcomes [135,403,404]. 

Recent material research on artificial SEI for Na-based batteries depends on oxides, polyanionic compounds, insertion materials (phosphates, phosphides, nitrides), metal selenides, metal oxides as conversion compounds, metal sulfides, carbon materials, and p block elements (Sb/Sn/phosphides, alloys). Pb-Na_15_Pb_4_, Bi-Na_3_Bi, Sn-Ge-Sb, and Sb-Cu_2_Sb are some of the alloys and embedded materials used for modulating the anodic structure and stability. Collective electrode materials of Na-based batteries and their performance characteristics are listed in Table 8 [405,406,407,408,409,410,411,412,413,414,415,416,417,418,419,420,421,422,423,424,425,426,427].

#### 6.2.5. Recent Approaches to Na Dendrite Suppression

Dendritic growth generates non-deformable cracks and electrode corrosion (degrading the components). A unique method was reported to control the dendrite severity via a Li-Na bimetallic alloy anode and organic additive (1,3-dioxolane additive) in an air battery system consisting of a Na-ion electrolyte. Na dendrite growth can be reduced using surface and additive modifications. In this context, the cation additive should not be condensed; thereby, it forms an electrostatic shield effect on Na deposits. Due to the minor ionic radius, Li+ ions persist with a stronger electrostatic shield effect than Na^+^. As Na^+^ is dominant, the reduced Li content results in serious dendrite growth and volume variation. To compensate for the Li ion loss and to suppress the dendrites, dioxolane additives can be added, which also improves the coulombic efficiency, with reduced volume changes [428]. 

A Na Super Ion Conductor (NASICON) with formula NaMP_3_O_12_ (M = Cd^2+^, Mn^2+^, Ni^2+^, Co^2+^, Zn^2+^, Al^3+^, Ga^3+^, V^5+^, Nb^5+^ and Sb^5+^) is a promising 3D tunnel electrolyte for Na capture and migration. Solid solutions of NaZr_3_P_3_O_12_ and Na_4_Zr_2_Si_3_O_12_ are extensively used in Na batteries. In particular, the rhombohedral phase of NASICON has higher stability than the monoclinic phase. Structural integrity and grain arrangement modifications in NASICON drastically impact ion conduction. 

Zebra batteries with a Na anode and NiCl_2_-FeCl_2_ cathode in a molten salt electrolyte have high energy density (790 Wh kg^−1^) but present safety concerns related to Na-ion batteries. A NiCl_2_ cathode can be conjugated with a Ni_3_S_2_ layered coating to prevent dendrite growth. However, the thickness and particle size of Ni in Ni_3_S_2_ significantly affect the cyclic performance. Thicker layers with larger particle sizes due to agglomeration tend to hinder the active dendrite prevention scenario. In comparison, minuscule particles with larger surface areas provide additional paths to accommodate the assorted volume consequences [429].

**Figure 30:**
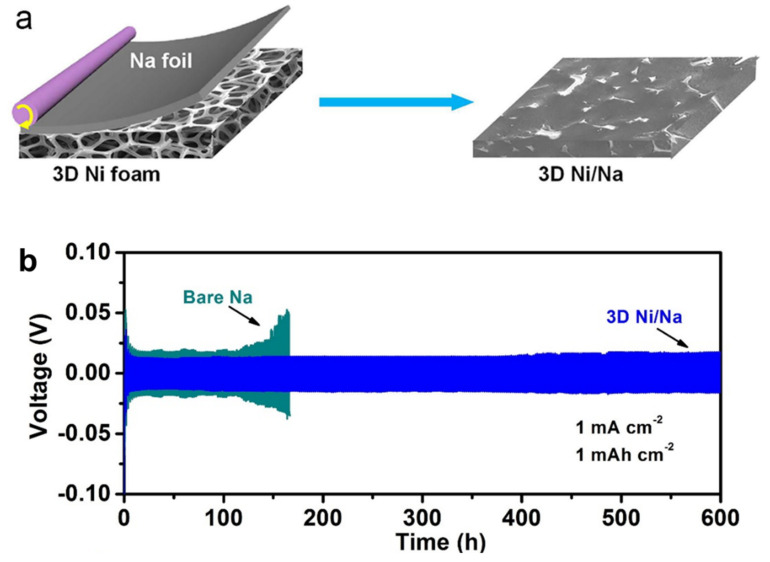
(**a**) Fabrication of 3D Ni/Na anode. (**b**) Voltage profile at 1 mA cm^−2^ for bare Na and Ni/Na (adapted with permission from [434]. Mat Lett., Elsevier, 2020).

Similarly, modified aluminum trichloride (AlCl_3_) with appropriate catholyte extensively pairs with a Na metal anode, producing high reversibility in Na-Al redox hybrid batteries. Essentially, the conversion mechanism of redox couples (AlCl_4_-Al_2_Cl_7_) has to be altered depending on the reaction flow [430]. Modifications of the cathode material speed up the cell reaction, generating high storage capacity. CuSO_4_ combined with carbon nanotubes has been employed as a new conversion-type cathode for NIBs. The high redox potential of the cathode, ~2.7 V (vs. Na/Na^+^), faster redox reactions (i.e., reduction of CuSO_4_ into Cu and Na_2_SO_4_), and the reversal process (oxidation) account for the high coulombic efficiency with dendrite-free structures [431]. The upright needle-like arrangement of MoS_2_/CoS_2_ on a Na anode fabricated using a simple hydrothermal method renders superior capacity (274 mAh g^−1^ at 10 A g^−1^) with a smoothening effect on dendrite edges [432]. Recently, Huanhuan Jia et al. examined various chalcogenides in terms of their structural properties and electrochemical characteristics. After several substitutions, compositional variations, and surface alterations, they found the combination of tin–silica and antimony-based Na as the optimal and best electrode material for Na batteries [433]. Moreover, a 3D Ni-Na substrate has shown higher cyclic efficiency due to the porosity and homogeneous Na deposition accompanied by reduced current density and overpotential [434]. Na metal foil was rolled mechanically into a Ni foam substrate. This 3D structure exhibited a steady cycle for 600 h at 1 mA cm^−2^ (overpotential of ~13 mV), as shown in Figure 30.

The demand for portable electronics such smartphones and computers is increasing dramatically. One research group introduced a new self-backup battery integrated triboelectric nanogenerator for automatic power restoration through battery systems. They employed a spring-like Na–carbon cloth composite and Na-rich vanadium phosphate cathode in a partial-solid-state polyvinylidene fluoride-hexafluoropropylene electrolyte. This combination of electrode–electrolyte showed the preferential infusion of Na into the sodiophilic carbon cloth with symmetric stripping. Thus, the model yielded a good rate capacity of 72.5 mAh g^−1^ at 5 C [435]. Energy storage systems with compact size and shape are needed for portable electronic devices. Integrating multi-shaped structures with various functional properties into a single domain structure can boost the energy stored. In this context, 2D sheets of titania stacked along with rGO and 1D carbon nanotubes emerge as a belt-like structure with appreciable ion storage capacity [253]. 

### 6.3. K-Based Batteries

#### 6.3.1. Altering the Salt and Design Chemistry

K-ion batteries (KIBs) are still under development and require ample research and theoretical models for optimization. Some of the significant drawbacks of KIBs are as follows: (i) the major side reaction between K and electrolyte in ester electrolytes, (ii) the high voltage requirement and poor anti-oxidation at high potentials in ether electrolytes, (iii) cathodic dissolution, (iv) dendrite growth, (v) limited capacity, (vi) poor kinetics and weak dissolution, and (vii) severe volume change. An electrolyte for K-based batteries must have a high dielectric constant, low viscosity, inertness with the cathode, thermal stability, non-toxicity, and be safely operatable at high flashpoints. Pham’s group measured the solvation energy of alkali metal ions in ethylene carbonate. They observed that K^+^ ions display a feeble solvation structure and energy among K, Li, and Na. K^+^ ions have the most negligible solvation energy concerning higher ion mobility and enhanced rate performance [436]. Moreover, highly concentrated EC-based electrolytes pose challenges as the solvation energy is low. Therefore, a less concentrated and less viscous solution must be used in KIBs. The concentration also affects insolubility. KClO_4_ is highly soluble in EC or PC at moderate concentrations (0.1 M), which is much lower than the Li battery counterparts (LiClO_4_ and LiBF_4_) [437]. KIB electrolytes can be stable with no dendrite formation even at low concentrations, saving material costs and space in the battery system.

According to recent research, ethylene carbonate is a frequently used solvent for KIBs. Zhao related the performance of ester and ether electrolytes. Ethylene carbonate in DMC has poor cyclability and rate performance due to the reduction in coulombic efficiency after 70 cycles. This decrease in efficiency occurs because of the enormous decay of DMC even at low potentials, as well as the generation of protrusions from the reduction products. In contrast, EC/PC and EC/DEC exhibit reversible cycles (~200 cycles) with high coulombic efficiency and a stable electrochemical window. Thus, the stability of SEI and dendrite formation are related to the solvents and the reduction products and their strength [436,438]. Some of the recently used electrolytes in K-ion batteries are listed in Table 9.

Ester-based electrolytes have also been employed in KIBs. Researchers investigated a potassium trifluoromethanesulfonimide/triethyl phosphate (KFSI-TEP) electrolyte and affirmed that K^+^ under this electrolyte forms stable SEI layers (stripping and plating) with significantly less volume change, potential hysteresis, and excellent charge efficiency of ~99% after 450 cycles. Compared to EC/DMC/DEC electrolytes, TEP offers better cyclic performance. Another research group reported contradictory results on the framing of electrolytic concentration. They noted that a high concentration of the KSFI-DME electrolyte increased the ionic conduction and stable passivation layers in a graphite anode. Using high concentrations of KFSI-DME, ~99.3% of coulombic efficiency and charge preservation above 80% were achieved beyond 99 cycles. The authors reported that a high concentration of DME-KFSI enabled the restriction of the oxidation reaction, leading to corrosion and dendrite formation [446]. This restriction occurs because DME and K^+^ ions are closely bonded in a highly concentrated electrolyte solution, where the HOMO level will be reduced, mitigating oxidative decomposition reactions. Diethylene glycol dimethyl ether (DEGDME) offers good electrochemical stability at high concentrations while using a Bi-C anode. The increase in the concentration of DEGDME increased the reversible battery capacity and formed a strong SEI. Mai found that the use of high concentrations of ether electrolytes may diminish the growth of dendrites, promoting a stable SEI and effective battery performance. Ether and ester electrolytes significantly improve the battery performance by preventing dendrite growth under the influence of the anode material, potential, additives, and the number of cycles. Wang and co-workers investigated ether and ester electrolytes using a graphite anode. The coulombic efficiency of DME (ether—69.6%) was lower than that of EC/DEC (ester—87.4%). In addition, the interface resistance of DME was lower than that of EC/DEC because of the poor SEI passivation and dendrite growth. Another research group revealed that ether-based electrolytes are superior and offer better electrochemical stability than the ester group [447]. The cyclic voltammetry [448] results showed a higher slope for DME, revealing the higher K^+^ diffusion. In other words, the linear structure of DME leads to a high electron number and reduced Stokes radius in K^+^-dimethyl ether (DME) compared to K^+^-ethylene carbonate (EC). These outcomes reveal the increased thermodynamic stability and capacity of K^+^-DME (ether) over K^+^-EC/DEC (ester). Many controversial research findings indicate that further studies in KIBs are needed as the SEI formation and kinetics of dendrites are still not clear in KIBs. In general, ether-based electrolytes are not suitable for high-voltage usages (>4 V). Nevertheless, future research may present possible solutions from a surface–interface optimization and material (additive) perspective to improve the voltage suitability of ether electrolytes. Modifying the cation solvation structure by electrolyte tuning and adjusting the interfacial kinetics favor higher energy density. The solvation structure depends on the electrolyte composition, salt concentration, additives, binders, capping agents, catalyst, and electrode design. Very recently, an anion-based solvent fabricated through solvent grafting exhibited a high K^+^ transfer rate with a dendrite-free electrolyte [448].

#### 6.3.2. Optimizing Solvent Formulations

Fluorine-based additives are employed in commercial LIBs for enhancing the stability of interfacial films. Difluorophosphate salts are used in K-ion batteries for remodeling the electrolyte chemistry to create a stable SEI framework [449]. Polymer additives and binders are utilized in K-ion batteries to significantly advance the molecular adhesion between the electrode–electrolyte interfaces and increase the rated capacity. PVDF, cellulose, PAN, PP, and PEO are used as reinforcing agents and electrochemical stabilizers. Polyacrylate sodium (PAAN) binder, with appreciable enlargement, mechanical strength, and sturdy molecular adhesion, is a promising candidate for restraining the volume occupancy and repetitive electrolyte degradation [450]. The most crucial issue of the K-ion battery is the flammability due to dendrite growth and buffer volume. Low-cost additives such as ethylene sulfate, propylene sulfate in phosphate, or sulfonyl-based electrolytes contribute to faster insertion–exertion of K^+^ with graphitic anodes. The additives modify the solvation structure of potassium ions and optimize the interface barrier while regulating dendrite puff-out [451]. Dimethyl methylphosphonate, trimethyl phosphate, trimethylsilyl) phosphate, hexamethoxycyclotriphosphazene, isopropyl phenyl diphenyl phosphate, and triphenylphosphate are some of the flame-retardant additives used in alkali Li, which may assist the functionalities of K-based batteries. Congruent studies considering influential parameters, components, and structure–property relations may identify suitable K-ion battery parameters. Bio-derived and recycled carbonaceous materials as cell components may significantly reduce the battery cost. 

#### 6.3.3. Use of Nanomaterials

The electrochemical performance of the K-ion battery has been dramatically improved with the development and use of low-dimensional structures. Nanomaterials shorten the diffusion path of K^+^ ions and improve the poor diffusion kinetics, accompanied by increased cyclic performance and rate capabilities. Nanostructured carbon, hollow carbon, graphene, graphite, CNT, carbon-based selenides, sulfides, oxides, and Prussian blue analogues are some widely used electrode materials in K-ion batteries. Sn-based alloys are used as anodic materials because of their high theoretical capacity but are limited with expanded volume and dendrite growth. Altering the design of Sn alloys with doping and encapsulation tends to suppress the dendrites, accommodating volume expansion. Polyaspartic acid-linked SnS_2_ 2D nanostructures encapsulated into an N-doped hollow carbon network provide an inflated interlamellar gap and accelerate ionic transport channels with autonomous dendrite crackdown [404]. Antimony, bismuth, and P block element-based metal alloys also offer high capacity with a dendrite-free SEI [452]. Microporous scaffolds with conjugated polymers and tunable properties have shown enhanced performance [453]. 

#### 6.3.4. Interfacial Modifications

Carbon-based anodes are primarily employed in K-ion batteries. However, the degradation of the interfacial layer results in poor cyclic stability. An interfacial layer grown ex situ before the cell reaction may contribute to suppressing dendrite formation and lead to a protective, stable SEI. Soaking of a thin potassium plate into the dominated inorganic electrolytes (potassium bis(fluorosulfonyl)imide (KFSI) in 1,2-dimethoxyethane (DME)) enables the instant formation of artificial SEI layers, which supplies and transports K^+^ ions for anodic diffusion. The formed synthetic SEI layers contain a KC8 intercalation compound formed during the potassiation [454]. Dengyun Zhai et al. described studies in which they wrapped a potassium metal anode with well-united carbon nanotubes (CNT). During the cycling process, the electrolyte–anode interphase underwent subsequent reduction, resulting in potassiation of CNTs. The wrapped CNT layer was stable and non-favorable for dendrite growth even at high current densities [455]. Potassium hexafluorophosphate has been utilized in Li-ion batteries as an additive to control dendrite growth, resulting in the formation of a stable PF_6_-LiF-rich passivation barrier due to the electrostatic shield effect of K^+^ ions. Similarly, based on theoretical assumptions, we reveal the idea of incorporating lithium hexafluorophosphate into a K^+^-rich battery, which may develop a strong SEI barrier, resulting in the prevention of dendrite growth [456]. 

Metal–air batteries are sustainable devices for storage due to the eco-friendly cathode, lightweight structures, and non-toxic materials. However, in the potassium-O_2_ battery, dissolved oxygen molecules react with the electrolyte species and consume the active anode material due to the lack of stable, protective layers. Artificially grown Sb-F may react with electrolytes, producing a aK-Sb-F composite layer over the anodic surface, which effectively mitigates the corrosion and dendrite structures [396]. MoS_2_/TeS_2_ with nitrogen doping can be used as a protective layer for anodic networks [457]. 

Core–shell structures with similar adaptation to NIBs have been used in K-based batteries. A Cu_2_O core with a polymer shell as an anode was recently investigated and exhibited high ionic conductance. The shell layers ensure the provision of high current through ultrafast K^+^ adsorption/desorption and assist in blocking dendrites as an artificial layer with enormous volume accommodation. The core (Cu_2_O) serves as a source reservoir for cation transfer and as a framework for stabilizing the structure during repeated cyclic loads. It has been found that an amorphous shell with a crystalline core offers better electrochemical stability. The interface of amorphous and crystalline bodies reduces the barrier surface energy. As a result, SEI deposits adsorb uniformly on the electrode surface, which indirectly narrows the bandgap, leading to rapid K^+^ transport [458]. 

#### 6.3.5. New Methods for K Dendrite Blockage

Solid electrolytes offer better safety than liquid electrolytes. However, the interface reactions are not consistently perfect and require optimization. Commonly, a solid electrode and liquid electrode result in the ignition and leakage of solvents. Alternatively, a semi-liquid anode and solid electrolyte may not cause seepage and provide ample interface contact. A thin layer of liquid Na-K alloy and solid metal (Na or K) occupying a significant fraction was employed as the anode under concrete electrolyte circumstances. The thin layer of liquid at the anode interphase blocked the dendrite growth with sufficient moisture and provided a continuous source for electrochemical reactions. It also acted as a source for surplus Na^+^ or K^+^ ions, freeing up space and weight. Upon higher cohesion and binding between the solid metal and semi-liquid alloy, the semi-liquid alloy exhibited relatively high stability. Liquid alloys replacing solid anodes may lead to better electrochemical kinetics, eliminating dendrites with capacity amplification [459].

**Figure 31:**
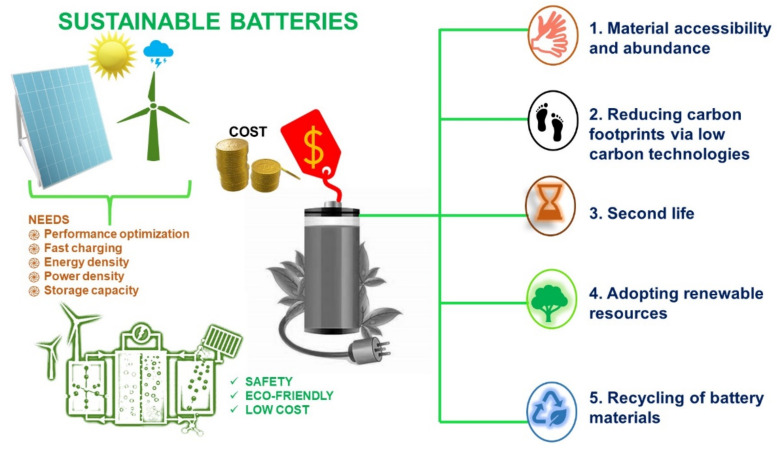
Approach needed for producing sustainable batteries.

**Figure 32:**
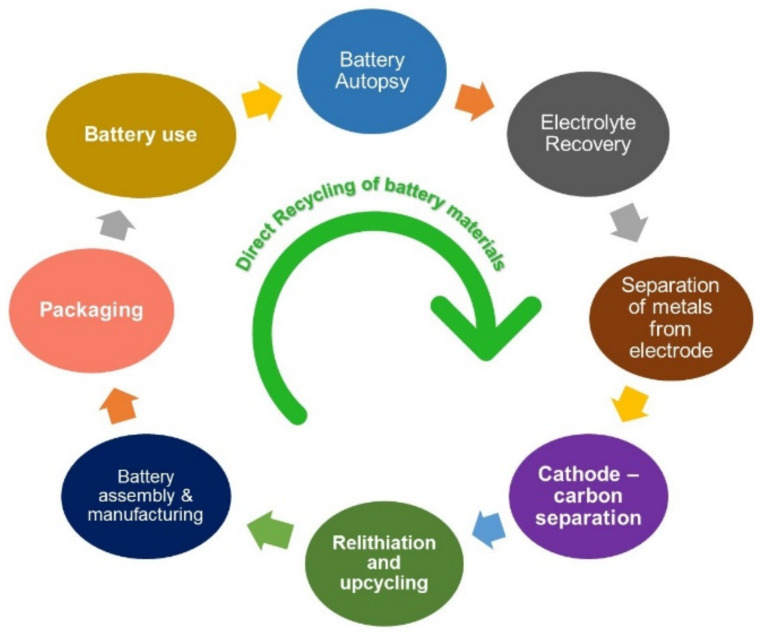
Direct and simple battery recycling flow diagram.

A circular economy approach accompanied by the reuse of materials may endorse the sustainability goals of present-day society. Various biological and available scrap materials can be reused as components in batteries [460]. However, the performance and energy density may fail with sophisticated chemical-based electrodes. There exist various routes to optimize and modify naturally derived materials in order to meet the current demands. Figure 31 illustrates the possible sustainable battery options. Li Tao’s group recycled soybeans into a porous carbonaceous material (hard carbon) and applied it as an anode in a K-ion battery, which showcased high capacity and a long lifetime (900 cycles). The crude carbon anode exhibited an average surface area and high interplanar distance with amorphous phase dominance. For better coulombic efficiency, a tinny film of aluminum oxide was kept on the hard carbon surface. Mxenes and plastic wastes have greater recycling viability and utilization potential for energy storage in the coming years [461,462,463,464,465]. Likewise, battery recycling may improve the secondary life with reduced material requirements in batteries. A basic battery recycling plan is shown in Figure 32.

A liquid anode with solid electrolytes is a fascinating approach to eliminating dendrites. A β-alumina solid electrolyte with a molten liquid potassium anode and carbon-based cathode holds the following advantages: (i) prevention of electrolyte leakage, (ii) increased battery safety with weight reduction, (iii) high space for volume expansion, (iv) dendrite-free cell structure, and (v) high-temperature resistance. A K–sulfur battery with a K molten anode@β-alumina and graphite cathode offers high stability and rate capacity of ~150 °C [466].

### 6.4. Other Batteries (Mg-, Zn-, Ca-, and Al-Based)

**Figure 33:**
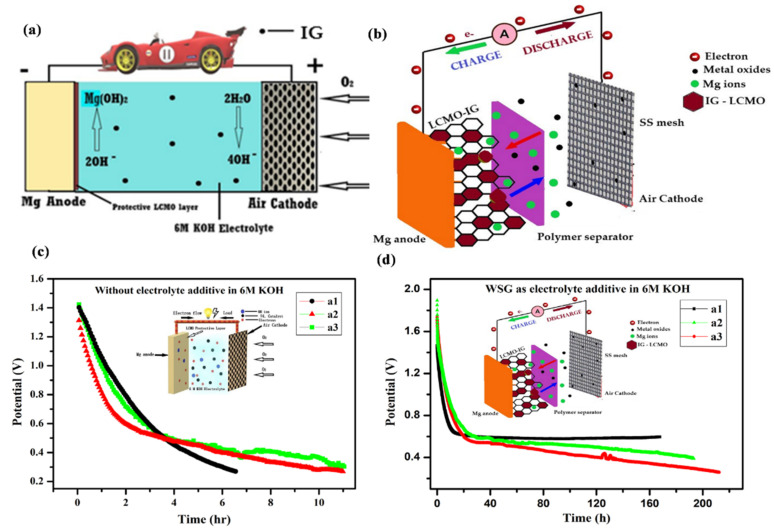
(**a**) Construction of Mg-air battery (**b**) Mechanism behind the operation of the Mg-air battery (**c**,**d**) Discharge performance of lanthanum-based electrodes for Mg–air batteries in 6M KOH and WSG-6M KOH (adapted with permission from [472]. Electrochem Soc., 2020).

Mg, Zn, and Al batteries cannot possess the same electrolytes and mechanisms as LIBs. Mg reacts with simple structured ions (ClO_4_, BF_4_), forming passivation layers on the anode surface [467,468]. Most of the passivation layers formed on the Mg anode remain impermeable to Mg^2+^ ions but degrade gradually after each cycle. Mg(PF_6_)_2_ with Mg/Mo_3_S_4_ has reversible dissolution and stability. The observed ionic conductivity was 28 mS cm^−1^ and operating potential up to >4.0 V vs. Mg/Mg^+^. In contrast, the Aurbach’s group claimed the lethal passivation of the (PF_6_)- anion by demonstrating the high voltage stability (>45 V) of LiPF_6_-DME in comparison with the low voltage stability (<0.5 V) of LiTFSI-DME [469]. The high voltage may be attributed to the formation of a stable protective film on the Mg anode. Aurbach’s group also investigated MgCl_2_ as an electrolyte additive and found that it inhibits passivation layer formation on PF_6_-based electrolytes. Another research group established the deposition of Mg, while Grignard reagents (R-MgX, X = Br, Cl, F, I) were dissolved in aprotic solvents [470,471]. It was observed that the combinations exhibited superior reductive stability. However, the free oxidation of Grignard reagents reduced the anodic performance, creating an unstable SEI. Recently, brindha et al, formulated a lanthanum-based perovskite coating on an anode and graphene as an additive in an aqueous Mg–air battery system, which was accompanied by a stable SEI and high capacity of 1595.3 mAh g^−1^. The high capacity was due to the barrier effect of the perovskite coating and the fast migration of ions through the steady SEI. As shown in Figure 33, the fabricated cell exhibited a discharge capacity between 1000 and 1600 mAh g^−1^ at various concentrations of coated perovskites, with a capacity retention of up to ~96% [472]. Morphological alteration of electrodes influences the metal dendrite formation across the SEI. In an Al–graphite metal battery, the natural Al_2_O_3_ protective layer dissolves easily upon illumination and stripping processes, which leads to glitches involving Al-O defects. Studies suggest that a porous Al anodic structure provides uniform ion flux, supporting the orderly arrangement of grains on the electrode surface. Sheets, prismatic, spherical, and well structures are also employed as battery components for the even distribution of ion flux [473]. 

Fibrous zincophilic carbon structures provide a superior electrochemical window with dendrite-free deposits in zinc–metal batteries [474]. Lamellar, hollow, and mounted structures are also utilized in rechargeable batteries. Mg^2+^, Ca^2+^, and Al^3+^ undergo severe corrosion in an aqueous battery accompanied by hydrogen evolution due to the thermodynamic instability of metals. Moreover, the hydrogen evolution increases the internal pressure of the cell, creating seepage. An insulating oxide film naturally forms on the anode surface, protecting it from corrosion and dendrite growth. The formed layer degrades due to changes in ion flux and mechanical weakness. Current density has a significant influence in controlling dendrite growth. Upon decreasing the current density, the needle-shaped dendrite transforms into a smooth globular shape, thus lowering the growth [475]. Other factors such as field control, ion flux regulation, mechanical shielding, and crystal orientation also play a vital role in dendrite suppression. A Ti_3_C_2_/Mxene composite has been identified as a universal anode material in Li, Na, K, and Ca batteries through first-principle density calculations. Li^+^ ions were able to form more stable SEI layers than K^+^, Na^+^, and K^+^, because of the effective ionic radius, which accounts for the ion interaction and layer coverage. The higher the ionic radius, the lesser the step coverage in the SEI with increased ionic interactions [19,476,477].

## 7. Emerging Concepts

In the 20th century, with technological advancements, there was an increased demand for durable, first-rate, defect-free products. Optimizing many battery components with suitable surface species, potential, thermal condition, and power point is burdensome. Meanwhile, the design of high-power batteries requires tremendous aerial capacity. Framing such batteries necessitates new equipment with less time utilization. In this context, 3D-printed electrodes provide high production levels of batteries with flexibility and low cost. Highly deformable functional electrodes such as N-doped carbon, Mxenes, CNT, r-GO, and polymer membranes have been fabricated using micro 3D printing [478,479]. Micro-lattice distortion, grain boundary defects, surface mismatch, porosity, and layer thickness can be adjusted using micro-printing technology, reducing the likelihood of dendrite growth due to defects and surface roughness [480]. The primary control parameters of micro-printing include an air gap between layers, extrusion temperature, thickness, material fill density, number of layers, print speed, and heat treatment temperature. Depending on the cell components, properties, and structure, the parameters mentioned above must be framed. Additive SEI layer deposition using printing technology ensures a highly precise ~μm-scale deposit. In the future, 4D printing models with successive changes in structure and shape with time, temperature, and environment may emerge in the field of battery design [481,482,483]. 

The construction of advanced energy storage devices relies not only on micro–nano structural electrode design and SEI formulations but also on device configuration, battery assembly, packaging, and device engineering. To meet the existing high-power demand, conjugated battery and supercapacitor electrodes with dual functionality seem to be a promising option. Battery supercapacitor hybrid devices work to unify high-energy-density battery electrodes and high-power-density supercapacitor electrodes, thus contributing a double benefit in a single device. Emerging hybrid device materials are Mxenes, MoS_2_, LaMnO_3_, Nb_2_O_5_, Ni, Co, Sb, Ru, and Bi-based compounds [484]. Simultaneously, photo batteries have attracted considerable research attention due to their environmental benignity and free-resource operation at low costs. In recent years, metal–organic frameworks have been preferred over other materials, as MOFs hold multiple characteristics such as synergism, catalysis, host–guest, and electrochemical stabilities according to appropriate material selection [485].

Hybrid battery–fuel cell technology has emerged recently because of the increasing greenhouse gas and carbon emissions. The majority of heavy automobiles entail high-temperature stability and high power. Present-day vehicles employ proton exchange membrane fuel cells, with increasing costs due to the Pt catalyst [486]. Alternate stimuli (nanocarbon, Mo, Ru) are adopted to address the cost issue. However, the sluggish reaction kinetics drastically reduce the output power. Battery electrochemistry and salt interaction are now tuned for supporting moderate energy density. However, the battery cannot power an entire vehicle alone because of its inadequate energy density. Combining batteries and fuel cells will be the most realistic solution. Discussion of the chemicals used, reactions, and optimization of the fuel cell are beyond the scope of this article. Recent studies suggest the inbuilt battery as auxiliary support to the main fuel cell power supply in parallel hybrid connections using a brushless high-power motor, mechanical transmitter, and gearbox. The produced electrical energy from the fuel cells and battery can be converted to automatic motor and drive stroke systems [487]. By this concept, we can use modern batteries with typical energy densities in high-power applications without interphase tuning or electrode design modifications. More in-depth research on design and control strategies for hybrid battery–fuel cell technology with breakthroughs may increase its commerciality in the future. 

## 8. Computational Designs and Simulation

**Figure 34:**
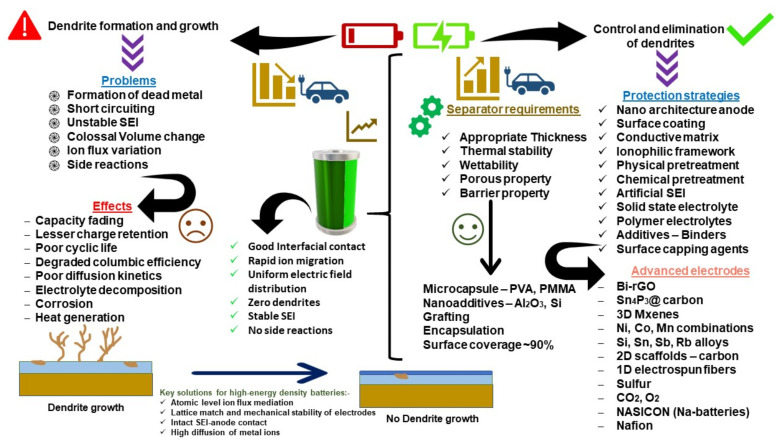
Outline of effects and control strategies for dendrites.

Highly reliable modeling frameworks depend on first-principle methods to determine the advanced material characteristics, fabrication steps, and post-cell assembly features using quantum computational mechanics and statistical equations [349,488,489,490,491,492,493,494]. Considering all the aspects of excellent electrode materials, such as affordability, security, space, distribution kinetics, endurance, and relevant components, it is challenging to explore such materials due to the stringent laboratory requirements. On the contrary, the computational simulations could be performing depending on the use of ab initio estimates on substances by adopting fewer electrochemical parameters to fix the lattice mismatch and crystal orientation problems in order to address the concerns discussed so far [491,495,496,497]. Moreover, we are in the preliminary phase of analyzing next-generation storage devices; therefore, the implementation of a secure database in order to devise guidelines for facilitating an expert search for battery materials is imperative. The aforementioned task would be crucial in addressing the concerns described here. The overall drawbacks of dendrite growth and possible solutions are depicted in Figure 34.

## 9. Conclusions and Perspectives

This review address the urgent need to expand the possibilities beyond Li ions to meet the energy demands engendered by gasoline. With alkali metal anodes, battery systems can achieve high energy densities closer to those of gasoline. In detail, we address the significant problems associated with metal batteries, focusing on theoretical growth models and experimental assessments. Conventional lithium-ion batteries with graphite anodes have reached their energy density and capacity limits (372 mAh g^−1^). Now, the use of Li metal anodes may enable high energy density. This is because the metal-ion conductive structure as a stable “host” and adequate surface protection addresses the many-sided obstacles in batteries, which is a vital step forward compared with earlier host notions. Additionally, the electrochemical performance and design principle highlight the effectiveness of developing safe and stable metal anodes. Despite efforts in Li metal anode development, its practical application remains limited due to the irregular Li metal reactivity and significant dimensional changes in the anode during the cyclic charge–discharge process, leading to challenges such as breakage of the SEI layer and dendrite deposition. The dendrites form uneven metal-ion flux, triggering thermal runway, and the stripping process aggravates dead metals, reducing the active metal surface for electrochemical reactions. The use of 2D and 3D rigid frameworks as anodic substrates is a significant option for controlling dendrites. Dendrites can be also controlled through decreasing the current density, stress relaxation, and applying an external pressure and temperature. Solid-state electrolytes and stable separators can also reduce the growth of dendrites. Though colossal efforts have been taken to mitigate dendrite growth, the loss of coulombic efficiency, volume variations, side reactions, and safety issues remain ubiquitous. The durability and SEI composition play an indispensable role in controlling dendrite generation. Regulating the SEI composition through surface coatings, additives, capping agents, and artificial layers may protect the anode surface in the long term. Adhesion and self-diffusion are other aspects that need further research efforts for optimization. Anode-less batteries, photo batteries, dual salt electrolytes, 3D framework electrodes, liquid anodes, flexible electrodes, solid-state devices, and nanocoatings are new strategies for improving battery efficiency. Research in Na and K batteries with sulfur, CO_2_ cathodes based on solid-state esters, or ether electrolytes with nano-additives (Mxenes, Carbon) and chalcogenides may facilitate novel findings. The adaptation of high-energy-density batteries should consider the following: (i) homogenizing metal-ion flux, (ii) reducing volume changes, (iii) barrier deposition to eliminate dendrites, and (iv) compact, well-built battery assembly and packaging.

The suppression and elimination of dendrites rely on various parameters. The interface energy between the substrate and metal atoms is most prominent, which can be engineered through mechanical dynamics and force organization such as diverting the electric field by applied Lorentz force or magnetic control, improving stress–strain relaxation by nanostructural designs, and lessening the ion diffusion barriers through higher activation energy. Design strategies aid in improving battery performance. Prismatic and pouch cell setups have more advantages in comparison to conventional cylindrical cell designs. The prismatic cell has better space utilization and flexibility. However, pouch and prismatic designs are expensive, less temperature-efficient, and may have a shorter lifecycle than cylindrical designs. Moreover, more scientific research works are needed for their commercial adaptability and performance optimization. Another critical factor is morphology, which holds the potential to regulate dendrite growth and suppression. Research on hollow, layered, prism, tetra-pods, sheets, and 0D structures may significantly boost future electrochemical optimization. In this context, the in situ assessment of battery electrodes, SEI, and dendrite growth via various material characterization techniques such as XPS, EELS, SAED, MFTIRS, AT-FTIR, Raman, XRD, SEM, neutron scattering, cryo-electron spectroscopy, and various other postmortem studies may lead to real-time data retrieval and fast-track processing of control strategies with higher accuracy. However, a few precautions, such as maintaining proper pressure, temperature, electromagnetic ray protection, cell sealing, and electrode–collector spacing, should be considered before experimenting with in operando characterization tools for real-time battery devices. Understanding the possible outcomes of current devices and integrating versatile instruments can also help to develop exceptional tools for interfacial studies of different electrochemical cells, such as Fe-S, Si-air, Na-S, K-air, and Zn-air batteries.

While discussing the safety and thermal management of Li-ion battery technology, external thermal management is undoubtedly a potential solution to circumvent thermal runaway, especially with liquid electrolytes (LEs). However, internal heat generation may still exist, leading to loss of energy and efficiency, and this must be reduced. Most generated heat in a cell is due to the elevated internal resistance, charge transfer resistance, and mass transfer resistance in solid-state batteries (SSB). However, heat generated due to SEI fracture and dendrite growth leading to thermal runaway is minimal in SSB. Solid-state batteries have fewer issues with SEI and dendrites, assuming the avoidance of gel or liquid electrolytes to wet the interfaces. However, Li-ion diffusion between the electrodes is the same regardless of the electrolyte used. Thus, external thermal control will assist in the dissipation of heat, maintaining the cells at low temperatures and guaranteeing safety.

Intelligent technologies are available for dendrite apprehension, battery locking during explosions, flame-retardant casing, novel battery assembly, and engineering. Further explorations and designs would facilitate safety with economic aspects. However, the scaling up of laboratory research to practical implementations in batteries is yet to be achieved. More attention is required to solve the critical issues associated with form factor, cell design (pouch, prismatic, or cylindrical), and large-scale development. Na and K batteries have strong potential to replace Li cells, yet their practical usage and energy density need to be addressed. Synergic strategies with commercial utility may encourage present-day explorations. We anticipate more groundbreaking advances in the field of metal–alkali batteries in the coming years.

## Figures and Tables

**Table 1:** Electrochemical characteristics and properties of precious battery metals.

	Parameters	Li	Na	Mg	Al	K	Ca	Zn
Chemical Property	Atomic No	3	11	12	13	19	20	30
	Atomic mass (g·mol^−1^)	6.941	22.98	24.3	26.98	39.09	40.08	65.38
	Electronic configuration	[He]2s^1^	[Ne]3s^1^	[Ne]3s^2^	[Ne]3s^2^3p^1^	[Ar]4s^1^	[Ar]4s^2^	[Ar]3d^10^4s^2^
	Metallic radius (pm)	152	186	160	143	220	197	134
	Ionic radius (pm)	76 (Li^+^)	102 (Na^+^)	72 (Mg^2+^)	53 (Al^3+^)	138 (K^+^)	100 (Ca^2+^)	74 (Zn^2+^)
	Covalent radius (pm)	133	155	139	126	203	171	118
	Van der Waals radius (pm)	182	227	173	184	280	231	139
	Ionization energy (KJ/mol)	520	496.9	738.1	577.9	418.7	590.2	908
	Pauling electronegativity	0.98	0.93	1.31	1.61	0.82	1	1.65
	Standard potential E° (V)	−3.04	−2.71	−2.38	−1.66	−2.92	−2.76	−0.76
	No. of isotopes	2	13	3	9	3	5	5
Physical Property	Melting point (°C)	180	98	650	660	63.5	842	420
	Boiling point (°C)	1330	883	1091	2470	759	1484	907
	Density (g/cm^3^)	0.535	0.968	1.731	2.701	0.862	1.554	7.133
	Earth crust abundance (%)	0.002	2.6	2.5	8.1	2.1	4.1	0.005
	Color	Silvery white	Silvery white	Gray–white	Silvery gray	Silvery white	Gray–yellow tint	Silvery–blue tint
Mechanical Property	Tensile strength (Mpa)	1.5	--	175–250	90	--	45	37
	Modulus of elasticity (Gpa)	4.6	10	65–100	70	29.6–38.1	18	96.5
	Mohs hardness	0.6	0.5	2.5	2.75	0.4	1.75	2.5
Electrochemical Property	Voltage vs. SHE (V)	Li - 3.04	Na - 2.71	Mg - 2.38	Al - 1.66	K - 2.92	Ca - 2.76	Zn - 0.76
	Theoretical capacity (mAh g^−1^)	3860	1166	903	8045	684		820
	Theoretical gravimetric energy density of M–O_2_ batteries (Wh kg^−1^)	3458	1605	3925	4312	935	2643	552
	Theoretical volumetric energy density of M–O_2_ batteries (Wh L^−1^)	7983	4492	4042	8056	2001	8829	3071
	Theoretical gravimetric energy density of	2615	1273	1684	1319	915	--	1850
	M–S batteries (Wh kg^−1^)							
	Theoretical volumetric energy density of	4289	2362	3221	2981	1589	--	3628
	M–S batteries (Wh L^−1^)							
	Desolvation energy in PC (kJ mol^−1^)	215.8	158.2	148.2	182.6	119.4	--	200
	Desolvation energy in EC (kJ mol^−1^)	208.9	151.3	202.4	--	112.8	--	--

**Table 2:** Specific capacity, advantages, and limitations of common anodes in LIBs.

Anode	Specific Capacity (mAh g^−1^) [108,109,110,111,112,113,114,115,116,117,118,119,120,121,122,123,124,125,126,127,128]	Advantages	Challenges
Insertion Compounds			
Graphite	100–400	• Small volume change in layered structure	• More side reactions
Hard carbon	200–600	• Decent operating potential	• Less coulombic and cell efficiency
CNTs	1116	• High performance	• High cost
Graphene	780/1116	• Safety and low cost	• Low reversible capacity
LiTi_2_O_4_	161	• Experiences two phase reactions • Good cyclability	• Difficult to synthesize • Preparation of mixed valence is not easy
LiTi_4_O_5_	175	• Extreme safety	• Very low capacitance
Li_4_Ti_5_O_12_	175	• 3 Li could be reversibly inserted • No SEI formation • Flat operating potential ~1.55 V	• Inherent electrical conductivity limits Li diffusion
LiCrTiO_4_	157	• Unit cell volume variation is observed at low voltage	• Electrochemical stability is inferior
TiNbO_7_	280	• No SEI formation • High reversible capacity • Eco-friendliness	• Moderate capacity fading upon cycling
SrLi_2_Ti_6_O_16_	262	• High diffusion coefficient • Excellent high drain performance	--
LiTi_2_(PO_4_)_3_	137	• Excellent cyclic performance	• Lack of electrical conductivity so carbon coating is necessary
TiO_2_	330	• High power capability	• Low energy density
TiO_2_-anatase	413	• Stable cyclability	• Unit cell volume variation • High insertion potential
TiO_2_-rutile	150	• Good stability	• Inferior electrochemical activity towards Li
Alloys and Materials			
Silicon	4212	• Strong bonding, stable SEI	• High irreversibility of charge • Colossal volume expansion
Germanium	1624	• Lower working potential	• Cost of material is high
Tin	993	• High power capability	• Huge volume variation
Antimony	660	• High-rate performance	• High cost and less abundance
Metal Oxides			
Tin oxide	790	• High storage capacity	• Suffers from volume changes
SiO	1600	• Good temperature stability	• Needs additives or alloy elements for high energy density
Fe_2_O_3_	1008	• Low cost • Eco-friendly	• Inherent electrical conductivity
CuO	674	• Good cyclability	• Volume changes, high voltage hysteresis
MnO	756	• Lower redox potential	• Inherent electrical conductivity
Mn_2_O_3_	1018	• Lower operating potential	• Huge ICL, high voltage hysteresis
CoO/Co_3_O_4_	715	• Favorable electrochemical properties	• Toxic and high-cost
Metal Sulfides			
FeS	610	• Very flat operating potential	• Low electrochemical stability
MoS_2_	167	• Very small ICL	• Inferior operating potential to TiS_2_

**Table 3:** Types of protrusions in metal anodes.

Type	Features	Width (μm)	Height (μm)	Aspect Ratio	Reference
Whiskers	Minimally branched and kinks surrounded by excess electrolyte	0.1–5	10–100	100	[175]
Dendrites	Branched fractal structures with pores	1–20	100–600	10	[176]
Globules	Interconnected globules nucleated on impurity sites	20–150	20–150	1–2	[177]
Trees	Narrow stem and branched top	10–500	100–500	1–3	[178]
Cracks	Developed through grains and structural instability in inorganic solid electrolyte	1–5	--	5	[179]
Moss	Pebble-shaped interconnected object with gaps and pores	10–50	--	1	[180]
Needle	Spiky, thorny thin structures with small gaps	0.5–10	5–200	1–2	[176]

**Table 4:** Common in situ analytical tools for SEI assessment.

SEI Components	Electrode	Electrolyte	In Situ	Ref.
LiF	Graphene-Li	LiPF_6_/EC:DMC	XRD	[222]
LiF	Glassy carbon/Li	LiPF_6_/EC:DMC	EELS	[223]
LiF	Cu/Li	LiPF_6_/EC:DMC	SERS	[224]
LiF	MoS_2_/Ti–Li	LiPF_6_/EC:DEC	SAED	[225]
LiEDC	Si/Li	LiPF_6_/EC:DEC	SFG-VS	[226]
LiEDC	Graphite/Li	LiClO_4_/EC:THF	SFG-VS	[227]
Li_x_PF	Graphite/Li	LiPF_6_/EC:DEC	FT-IR	[228]
Li_2_CO_3_	Carbon/Li F	LiPF_6_/EC:DMC	FT-IR	[199]
Li_2_CO_3_	Carbon/Li	LiClO_4_/EC:DMC	FT-IR	[229]
Li_2_CO_3_	Cu/Li	LiPF_6_/EC:DMC	SERS	[224]
Li_2_CO_3_	Si/Li	LiPF_6_/EC:DMC	FT-IR	[230]
Li_2_CO_3_	Graphite/Li	LiPF_6_/EC:DEC	DRIFT	[231]
Li_2_CO_3_	CVD (artificial SEI)	LiNi_0.6_Co_0.1_Mn_0.3_O_2_	DRIFT	[232]
ROCO_2_Li	Sn/Li	LiPF_6_/EC:DMC	MFT-IRS	[233]
LiOH	Glassy carbon	LiPF_6_/EC:DMC	EELS	[223]
Li_2_O	Li/ITO	PEO-LiN(CF_3_SO_2_)_2_	Ellipsometry	[234]
Li_3_N	Li	LiPON	XPS	[235]
ROCO_2_Li	Graphite/Li	LiPF_6_/EC:DMC	FT-IR	[236]
ROCO_2_Li	Graphite/Li	LiClO_4_/EC:DMC	ATR-FT-IR	[229]
PEO	C-Coated ZnFe_2_O_4_/Li	LiPF_6_/EC:DMC	Raman	[237]

**Table 5:** Recent electrolytes, cathodes, and capacities observed in Li-based batteries.

Recent Cathodes	Electrolyte	Attained Voltage (V)	Capacity (mAh g^−1^)	Ref.
K-doped LiMn_2_O_4−y_S_y_	1 M LiPF_6_	3.01–4.5 V	116 mAh/g	[268]
Layered Li(Ni,Mn)O_2_-coated LiCoO_2_	LiPF_6_ in ethylene carbonate	4.47 V	112 mAh g^−1^ at 10 C	[269]
Biomass-carbon@FeS_2_	1 M lithium bis(trifluoromethanesulfonyl)-imide (LiTFSI) in a mixed solvent of 1,3-dioxolane and 1,2-dimethoxyethane	3.06 V	850 mAh g^−1^ after 80 cycles at 0.5 C	[270]
Nitrogen–carbon-doped V_2_O_5_	LiPF_6_ in ethylene carbonate (EC)	3.9 V	440 mAh g^−1^	[271]
Li[Li_0.2_Ni_0.13-x + y/3_Co_0.13-x + y/3_Mn_0.54-x + y/3_]Al_x_Zr_y_O_2_	Standard electrolyte	4.4 V	245.5 mAh g^−1^ at 25 mA g^−1^	[272]
Li_1.2_Mn_0.6_Ni_0.2_O_2_	LiFP_6_ electrolyte in EC	2.0–4.8 V	266 mAh g^−1^	[273]
LiFeSO_4_F	LiFP_6_ electrolyte in EC	3.9 V	60 mA h g^−1^ even at 5 C	[274]
Poly-(1,4-anthraquinone)/carbon nanotube	Standard electrolyte	3.8	233 mAh g^−1^	[275]
LiVPO_4_F/C LiNi_0.5_Mn_1.5_O_4_ LiVPO_4_F/graphene	1.3 mol L1 LiPF_6_ LiPF_6_ 1 mol L1 LiPF_6_ in EC, EMC, DMC	4.2 V 3.6–4.2 V 3.1 V	116.5 mA h g^−1^ 118.1 mAh g^−1^ 168 mAh g^−1^	[75,276,277,278,279,280]
Bismuth oxyfluoride @ CMK-3 nanocomposite	LiPF_6_ (1 M) in dimethyl carbonate	4.0 V	148 mA h g^−1^ after 40 cycles	[281]
High-entropy ceramics		4.2 V	307 mAh g^−1^	[282]
Li_2_(Ir_0.1_Mn_0.9_)O_3_	1 M LiPF_6_ dissolved in ethylenecarbonate and dimethyl carbonate		192 mA h·g^−1^	[283]
LiNi_0.91_Co_0.07_Y_0.02_O_2_	1 M LiPF_6_	3.6–4.2 V	225 mAh g^−1^	[284]
Li_1+x_Mn_2-x_O_4_	LiPF_6_	3 V	300 mAh g^−1^	[285]
LiMn_1.8_Ti_0.2_O_4_	1.2 mol dm^−3^ LiPF_6_ in ethylene carbonate	2.0–4.6 V	215 mA h g^−1^	[286]
LiNi_0.4_V_0.1_Mn_1.5_O_4_	LiPF_6_	3.5 V	99.5 mAh g^−1^	[287]
Polyphenyl film-coated LiNi_0.5_Mn_1.5_O_4_	LiPF_6_	3–4 V	136.7 mAh g^−1^	[276]
Mg-doped Li_1.5_[Mn_0.75_Ni_0.25_]O_2+_*_δ_*	1 M LiPF_6_	3. 5 V	248.6 (20 mA g^−1^)	[288]
Li_1.16_(Ni_0.18_Co_0.10_Mn_0.52_Fe_0.02_)O_2_	1 M LiPF_6_ in 1:1 wt% EC:DMC	3.8 V	>100 mA h g^−1^	[282]
NaCoHCF	molten salt	3–3.8 V	90 mAh g^−1^ at 20 C	[289]
Li_1.12_Na_0.08_Ni_0.2_Mn_0.6_O_1.95_F_0.05_	I M LiPF_6_	3.6 V	167 mAh g^−1^ at 5 C	[290]
LiNi_0.6_Mn_0.2_Co_0.2_O_2_	1.0 M LiPF_6_ in EC/EMC	3.0–4.6 V	180 mAh g^−1^	[291]

**Table 6:** Common additives and their functionalities.

Efficient Additives	Purpose
Cs^+^	Dendrite growth tip softens and becomes dome-shaped rather than needle-shaped (before additive)
CsPF_6_	Diminishes mossy protrusions through electrostatic shield mechanism
RuF	Works even at low concentrations by actively lowering the electric field
AlCl_3_	Forms nanosized Al-hydroxide layer covering the anode surface, improves storage capacity
LiF	Increases Li transport with trace-controlled water molecules
Vinylene carbonate	Readily breaks the P-F bonds in LiPO_2_F_2_, improves ion migration
SO_2_Cl_2_	Increases ion migration
Propylene carbonate	Accelerates Li reactivity by ion diffusion
Fluro ethylene carbonate	Forms LiF-rich, stable SEI
Dimethylsulfate	Sulfur layer passivation reduces dendrite growth
*N,N*-dimethylethanolamine (DMEA)	Organic moieties forming stable SEI
Tetraethylorthosilicate in Li-O_2_ battery	Protects from anodic corrosion by forming stable film over anode
SiCl_4_ in Li-S battery	Increases coulombic efficiency

**Table 7:** Possible SEI components in Li-based electrolytes.

Electrolyte	SEI Components
LiBF_4_ in propylene carbonate	LiF, Li_2_CO_3_, LiOH, carbonate, hydrocarbons
LiBF_4_ in r-BL	LiF, LiOH
LiBF_4_ in THF	LiF, hydrocarbons
LiClO_4_ in PC	LiF, LiOH, Li_2_O, LiOCO_2_R
LiAsF_6_ in DMC:EC	ROCO_2_Li, Li_2_CO_3_
LiPF_6_ in VC	Li_3_N, LiNO_2_, ROCO_2_Li (CH_2_CH_2_O), C-F, LiF
LiNO_3_ in EC	LiN_a_O_b_
LiNO_3_ in DME	Li_2_S2O_3_, LiN_x_O_y_, Li_2_S_2_
Li_2_S_6_ in DME/DOL	Li_2_S_2_, Li_2_S
LiTFSI in DME	Li_2_S_2_O_3_, Li_3_N, Li_2_S
LiTFSI in DOL	Li_2_NSO_2_CF_3_, LiF, Li_2_S_2_O_4_, Li_2_S

**Table 8:** Na-ion battery materials and performance characteristics.

Refs. [405,406,407,408,409,410,411,412,413,414,415,416,417,418,419,420,421,422,423,424,425,426,427]		
1. Anode Materials		
Carbon-Based		
Compound	Specific Capacity (mAh g^−1^)	Operating Voltage and Performance Characteristics
Hard carbon	~300	Closed nanopore structures, 40% irreversible capacity
Biomass-derived hard carbon	430	Capacity fading of 2.5% after 200 cycles
Aromatic structure-derived carbon	321	Strong pyrolization involved, high thermal stability
Petroleum coke	80–100	Less stable SEI
Electrospun carbon fibers	233	Capacity fading of 2.3% after 200 cycles
Lignin derived electrospun fibers	293	Less than 10% of capacity fading
Polyacrylonitrile fibers–carbon	140	Current density of 500 mA g^−1^
Cellulose-derived carbon fibers	255	Current density of 40 mA g^−1^
N-doped carbon fibers	134	Current density of 200 mA g^−1^ Uniform nitrogenation leads to improved stability
Nanocarbon spheres	~240	>400 cycles at 5 C
Nanocelular foam	152	>1600 cycles, less than 10% capacity fading
Expanded graphite	280	Current density 20 mA g^−1^
Carbon, CaC_2_-doped Mxene	430–582	>50 cycles at 30 mA g^−1^
Metal Alloys		
Na-Sn	776	>100 cycles, free volume and elongated contacts
Sn-Sb-Carbon	400	>80 cycles at 30 °C
Cu-Sn	400	Nanosized SEI, 100 cycles
Cu-Sn-TiC	150–180	>100 cycles at 100 mA g^−1^
Sn-Sb	600	160 cycles, formation of intermetallides
Sb-C	610	Sb ~ 30 nm in size, >300 cycles
Sb-rGO	400	Current density 20 mA/g, >30 cycles
SiC-Sb-Carbon	440–500	100 cycles, current density 100 mA g^−1^
Pb-C	464	>50 cycles, high capacity retention
Oxide Materials		
SnO	550	Flower-like morphology of SnO favored high stability
SnO_2_–carbon	500	>50 cycles, less capacity fading
TiO_2_	335	3.5–4 V, >100 cycles
SnO_2_-Fe_2_O_3_	300	Current density 25 mA g^−1^
Na_2_Ti_3_O_7_	93	Current density of 5 C after 50 cycles
NaVO_2_	120	~1.6 V
Na–vanadium-based oxides, NaFeTiO_4_	150–300	0.8–2.6 V
Other Anodes		
Sn_4_P_3_	700	Current density of 50 mA g^−1^
SnSe	700	>50 cycles at 50 mA g^−1^
MoSe_2_	350	High charge retention
NASICON, sodium vanadium complexes	67–112	1000 cycles at 10 C
2. Cathode Materials		
Na_x_MnO_2_	308	Structural changes lead to capacity fading
Na_x_CoO_2_	107	~3.6 V
NaFeO_2_	100	~3.3 V, degradation after 3.8 V
NaCrO_2,_ Na_x_VO_2_	115	Ability for sodium reversibility
Na_2/3_[Ni_1/3_Mn_2/3_]O_2_	173	3.3–4.5 V
Na_2/3_[Fe_1/3_Mn_2/3_]O_2_	190	3. 4 V
Na_0.6_Li_0.6_[Mn_0.72_Ni_0.18_Co_0.10_]O_2_	200	2–4 V at 20 mA g^−1^
Phosphate-Based Materials		
NaFePO_4_	90	Current density of 90 mA/g, ~3.3 V
Na_2_FeP2O_7_	97	>150 cycles, ~3.2 V
Na_2_MnP_2_O_7_	78–70	3.6 V
Na_3_MnPO_4_CO_3_	125	2–4 V, thermal stability
Na_4_Co_3_(PO_4_)_2_P_2_O_7_	95	Low volume expansion
Na_3_V_2_(PO_4_)_3_	100	~3.3 V
NaVPO_4_F	97–110	2–3 V
Other Cathodes		
NaV_6_O_15_	80–140	3–3.5 V
NaFeF_3_	237	2–3.3 V
Chalcogenides	300–600	2–3.4 V
Prussian blue analogues	80	3.5–4 V

**Table 9:** Electrolytes used in K-ion batteries and their internal coulombic efficiency (ICE).

Electrolyte	Electrode	Current Density (mA g^−1^)	ICE (%)	Ref.
KPF_6_-EC/PC	Graphite	20	66.5	[439]
KPF_6_-EC/DEC	Bi-rGO	50	47	[440]
KFSI-EC/DEC	Sn-Carbon fiber	50	64.17	[441]
KFSI-DME	K-Cu	0.05	99	[442]
KTFSI	Bi@C	10	90	[443]
KTFSI-DOL/DME	PAQS-K	20	90.1	[444]
KPF_6_-EC/EMC	3D SnSb	500	58.5	[445]

## Data Availability

Not applicable.
